# Interlayer Interactions as Design Tool for Large-Pore COFs

**DOI:** 10.1021/jacs.1c06518

**Published:** 2021-09-08

**Authors:** Sebastian T. Emmerling, Robin Schuldt, Sebastian Bette, Liang Yao, Robert E. Dinnebier, Johannes Kästner, Bettina V. Lotsch

**Affiliations:** † Nanochemistry Department, Max Planck Institute for Solid State Research, Heisenbergstraße 1, 70569 Stuttgart, Germany; ‡ Department of Chemistry, University of Munich (LMU), Butenandtstraße 5−13, 81377 Munich, Germany; § Institute for Theoretical Chemistry, University of Stuttgart, Pfaffenwaldring 55, 70569 Stuttgart, Germany; ∥ Institute for Inorganic Chemistry, University of Stuttgart, Pfaffenwaldring 55, 70569 Stuttgart, Germany; ⊥ E-conversion and Center for Nanoscience, Lichtenbergstraße 4a, 85748 Garching, Germany

## Abstract

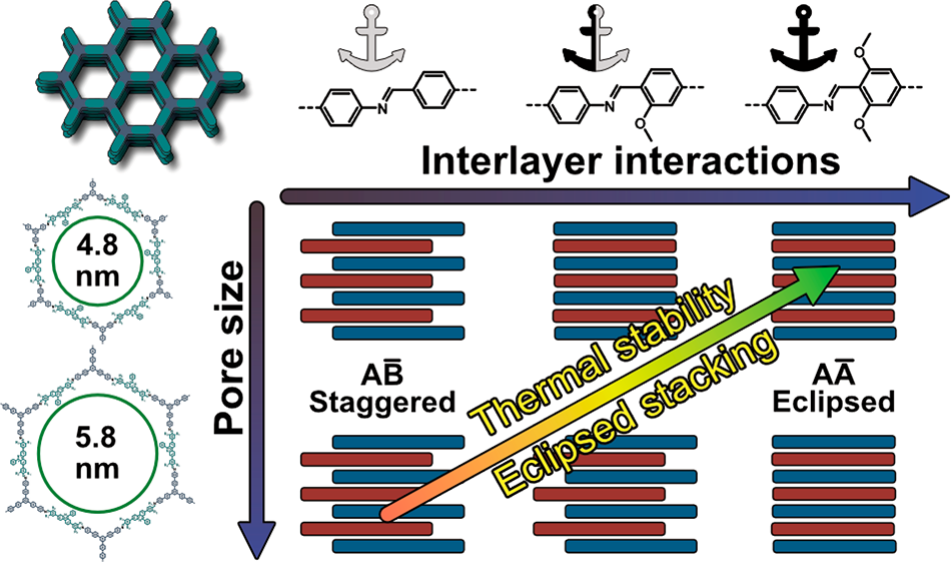

Covalent organic frameworks (COFs) with a pore size beyond 5 nm are still rarely seen in this emerging field. Besides obvious complications such as the elaborated synthesis of large linkers with sufficient solubility, more subtle challenges regarding large-pore COF synthesis, including pore occlusion and collapse, prevail. Here we present two isoreticular series of large-pore imine COFs with pore sizes up to 5.8 nm and correlate the interlayer interactions with the structure and thermal behavior of the COFs. By adjusting interlayer interactions through the incorporation of methoxy groups acting as pore-directing “anchors”, different stacking modes can be accessed, resulting in modified stacking polytypes and, hence, effective pore sizes. A strong correlation between stacking energy toward highly ordered, nearly eclipsed structures, higher structural integrity during thermal stress, and a novel, thermally induced phase transition of stacking modes in COFs was found, which sheds light on viable design strategies for increased structural control and stability in large-pore COFs.

## Introduction

Covalent organic frameworks (COFs) are highly stable, permanently porous and crystalline materials assembled from organic building blocks to form defined periodic networks.^1^ Due to their structural and chemical diversity, COFs have been attracting great interest for various applications such as catalysis,^2−5^ gas separation and storage,^6−8^ water harvesting,^9,10^ energy storage,^11−13^ and chemical sensing.^14−16^ To design and optimize COFs for applications, materials with high crystallinity and accessible surface areas, as well as chemical and thermal stability, are targeted.^17^ In particular, well-defined structures with accessible docking sites and large pore sizes become increasingly important to tailor COFs for distinct applications. While micro- and small mesoporous COFs are widespread today, large-pore COFs with pore diameters exceeding 5 nm are still rare.^18−23^ With a large open pore structure, such COFs could host bulky guest molecules such as biomolecules^24−26^ or allow sufficient diffusion of substrates and products in heterogeneous catalysis, making them ideal candidates to further widen the scope of applications for COFs.

In 2014, Fang et al.^20^ designed the first polyimide-based COF with a pore size exceeding 5.0 nm, which could absorb the large dye molecule rhodamine B in its channels. Recently Zhao et al.^23^ synthesized an ester-linked COF spanning 10 phenylene units at one edge. While the polyimide COF crystallizes—as all other examples known to date—in a slipped configuration, slightly reducing the apparent pore size, the ester-linked COF showed a significantly smaller pore size in dry conditions than anticipated. However, low crystallinity due to the high flexibility of the structure precluded a definite structure analysis. Controlling the synthesis and structure of large-pore COFs to maximize their pore size seems to impose unique problems compared to COFs with smaller pores. Besides synthetic challenges such as reduced linker solubility with increasing length, the observed effect of layer slipping and potential pore collapse upon removal of guests have to be considered.^27^ For mesoporous systems such as COFs, large interfacial energies are expected due to their high surface areas, leading to a natural tendency to minimize their free energy by the closure of the energetically unfavorable pores.^28,29^ For COFs this tendency could be a lateral slipping of the layers to decrease pore size and surface area or ultimately a pore collapse, while both are counterbalanced by the strong interlayer stacking interactions and rigid linkers, keeping the porous structure intact. The so far generally observed slipping of layers within large-pore COFs may be the result of an imbalance between increasing pore aperture and relatively larger free volume, while the same “wall-thickness” and therefore similar interlayer interactions are maintained. The total free energy of the system may then become unfavorable, and the system is forced to minimize its free energy by pore collapse or layer slipping instead of maintaining nearly eclipsed structures. We thus hypothesize that increasing the interlayer interactions might help to anchor the layers in nearly eclipsed stacking modes and prevent slipping or even pore collapse. Considering these observations, an in-depth understanding of the factors affecting pore structure and structural stability is necessary to successfully obtain large-pore COFs without sacrificing crystallinity and porosity.

In recent years a great deal of attention has been devoted to the task to increase crystallinity and surface area of COFs, e.g., by modulating reversibility during the formation process or facilitating beneficial stacking interactions, both of which can be instrumental in the design of large-pore COFs.^30−32^ In addition, an increasing repertoire of new linkages or postmodification of linkages was developed to extend the portfolio of synthetic tools to create chemically stable networks that withstand harsh chemical conditions.^23,33−36^ However, in terms of thermal stability Evans et al.^37,38^ recently showed that TGA analysis is not sufficient to assess thermal stability of the frameworks. They found that the structural integrity and crystallinity of the networks could be compromised by buckling of the layers under thermal stress at significantly lower temperatures than the TGA measurements suggest. Given the high flexibility of larger COF structures,^23^ thermal stress can have an even bigger impact on their structural integrity.

Herein, we reveal the structure-directing influence of interlayer interactions in two isoreticular series of 2D large-pore COFs. We pay specific attention to the effect of interlayer interactions on structure and pore size and investigate the structural integrity of large-pore COFs under thermal stress. Methoxy groups are introduced into the COF structures in meta position to the imine bond to alter and modulate the interlayer interactions by reducing the inherent dipole moment of the imine linkage^39^ and by adding favorable interlayer hydrogen bonding.^40^ By varying the amount of methoxy functionalization and the pore size, a series of six isoreticular, hexagonal COFs with a maximum pore size of 5.8 nm are prepared. Recursive X-ray powder diffraction (XRPD) simulations and refinements, sorption analysis, and density functional theory (DFT) calculations show that increasing the pore size leads to a tendency toward staggered layer arrangements, which can be reverted into eclipsed arrangements by increasing the stacking interaction and anchoring the layers in nearly eclipsed stacking. *In situ* XRPD and correlating *ex situ* sorption analysis establish a close connection between interlayer interactions and thermal stability and reveal a novel thermally induced phase transition from eclipsed to staggered conformation at temperatures as low as 120 °C. Our observations demonstrate that modulating interlayer interactions is a viable tool to influence COF stacking and maximize thermal stability and pore sizes in large-pore COF systems.

## Results and Discussion

### Synthesis and Structural Analysis of Phenylphenanthridine COFs

**Scheme 1:**
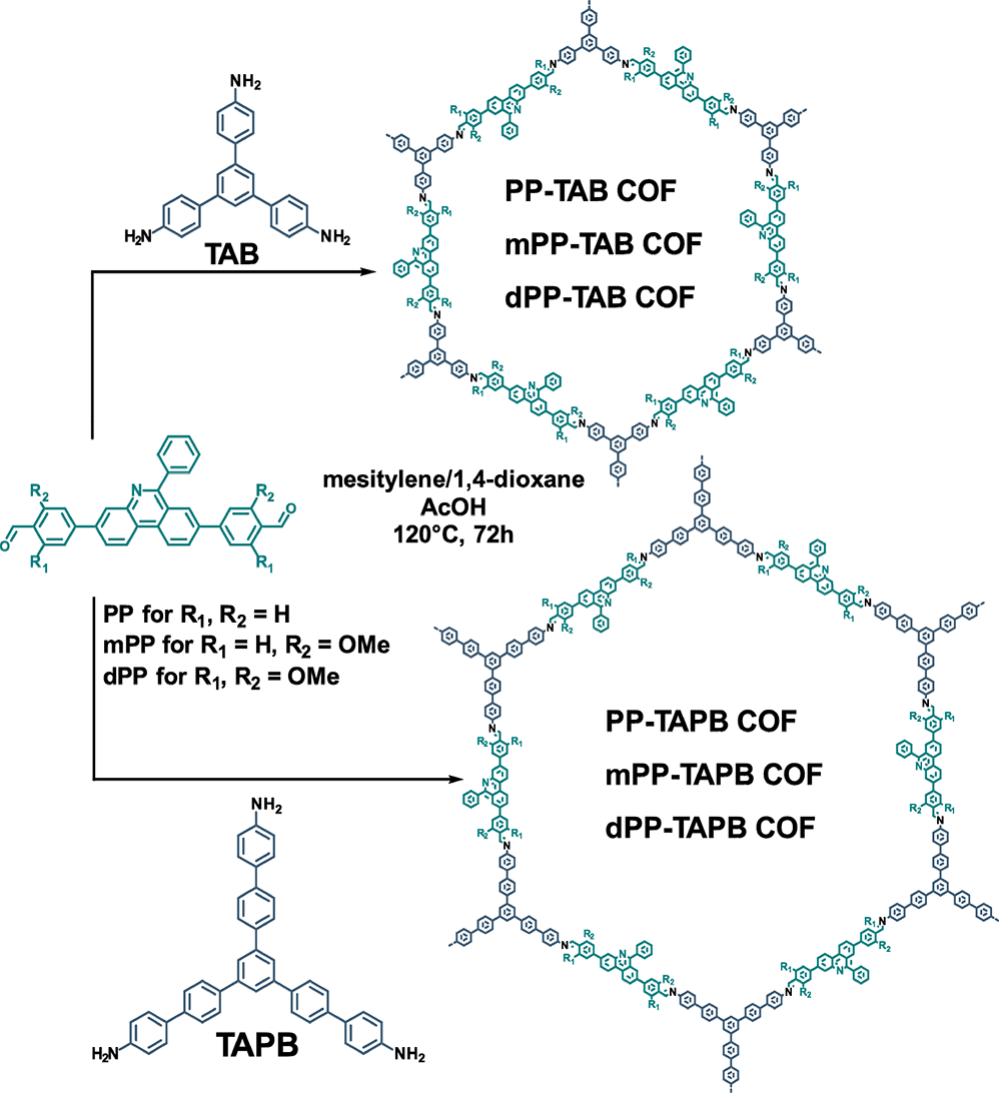
Synthesis of PP-TAB, mPP-TAB, dPP-TAB, PP-TAPB, mPP-TAPB, and dPP-TAPB COFs to Obtain a Set of Six Isoreticular COFs with Varying Pore Sizes and Interlayer Interactions

To study the influence of interlayer interactions on the structure, crystallinity, porosity, and thermal stability of large-pore COFs, we selected three phenylphenanthridine-based building blocks, 4,4′-(6-phenylphenanthridine-3,8-diyl)dibenzaldehyde (PP), 4,4′-(6-phenylphenanthridine-3,8-diyl)bis(2-methoxybenzaldehyde) (mPP), and 4,4′-(6-phenylphenanthridine-3,8-diyl)bis(2,6-dimethoxybenzaldeyde) (dPP), in combination with 5′-(4-aminophenyl)-[1,1′:3′,1″-terphenyl]-4,4″-diamine (TAB) or 1,3,5-tris[4-amino(1,1-biphenyl-4-yl)]benzene (TAPB). By the combination of these building blocks, six isoreticular COFs, PP-TAB, mPP-TAB, dPP-TAB, PP-TAPB, mPP-TAPB, and dPP- TAPB, with varying amounts of methoxy groups—zero to two regarding each imine bond—and differing pore sizes were obtained (Scheme 1). The descriptors “m” and “d” denote mono- and dimethoxy-functionalized linear linkers.

The successful conversion of the building blocks into imine-linked COFs was established by Fourier transform infrared analysis (FT-IR), ^13^C cross-polarization magic angle spinning (CP-MAS) NMR spectroscopy, nitrogen sorption analysis at 77 K, and XRPD. FT-IR reveals the full consumption of starting materials by the disappearance of typical amine and aldehyde stretching bands at around 3300 and 1670 cm^–1^, respectively. Further, the appearance of a new imine-stretching band around 1620 cm^–1^ confirms the successful formation of imine linkages for all six COFs (Figures S1–S6). ^13^C CP-MAS NMR spectra show the typical imine signal at around 160 ppm and reveal that no residual aldehydes are left for all six COFs (Figure S33). The signals of PP-TAB, PP-TAPB, and mPP-TAPB display significant broadening and overlapping, indicating higher disorder, compared to the spectra of mPP-TAB, dPP-TAB, and dPP-TAPB, which show sharper and more defined signals.

### Structure Analysis

**Figure 1:**
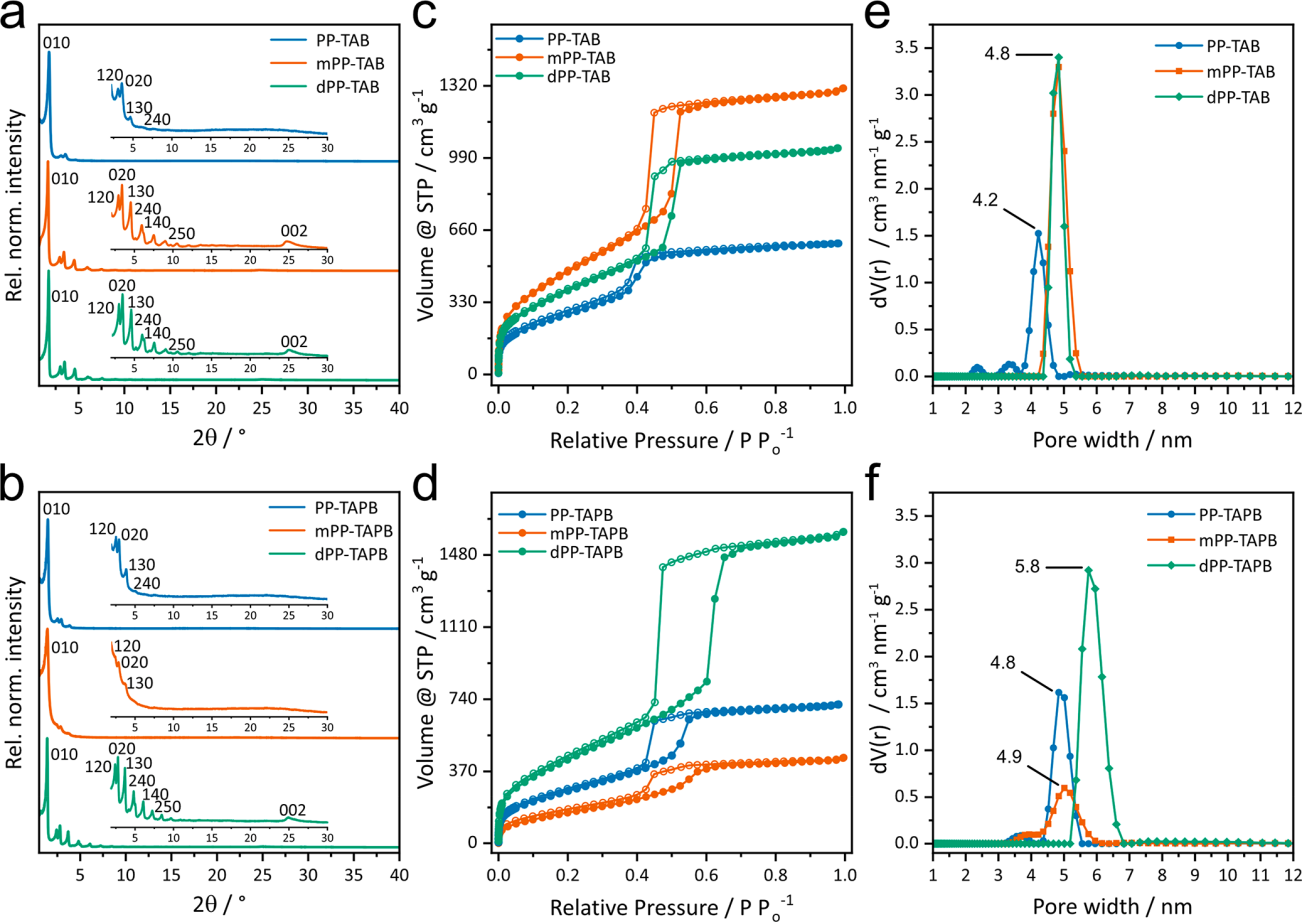
XRPD patterns (Cu-K_α1_) (a, b), nitrogen sorption isotherms at 77 K (c, d), and calculated pore size distributions (e, f) of PP-TAB, mPP-TAB, dPP-TAB, PP-TAPB, mPP-TAPB, and dPP-TAPB COFs.

**Figure 2:**
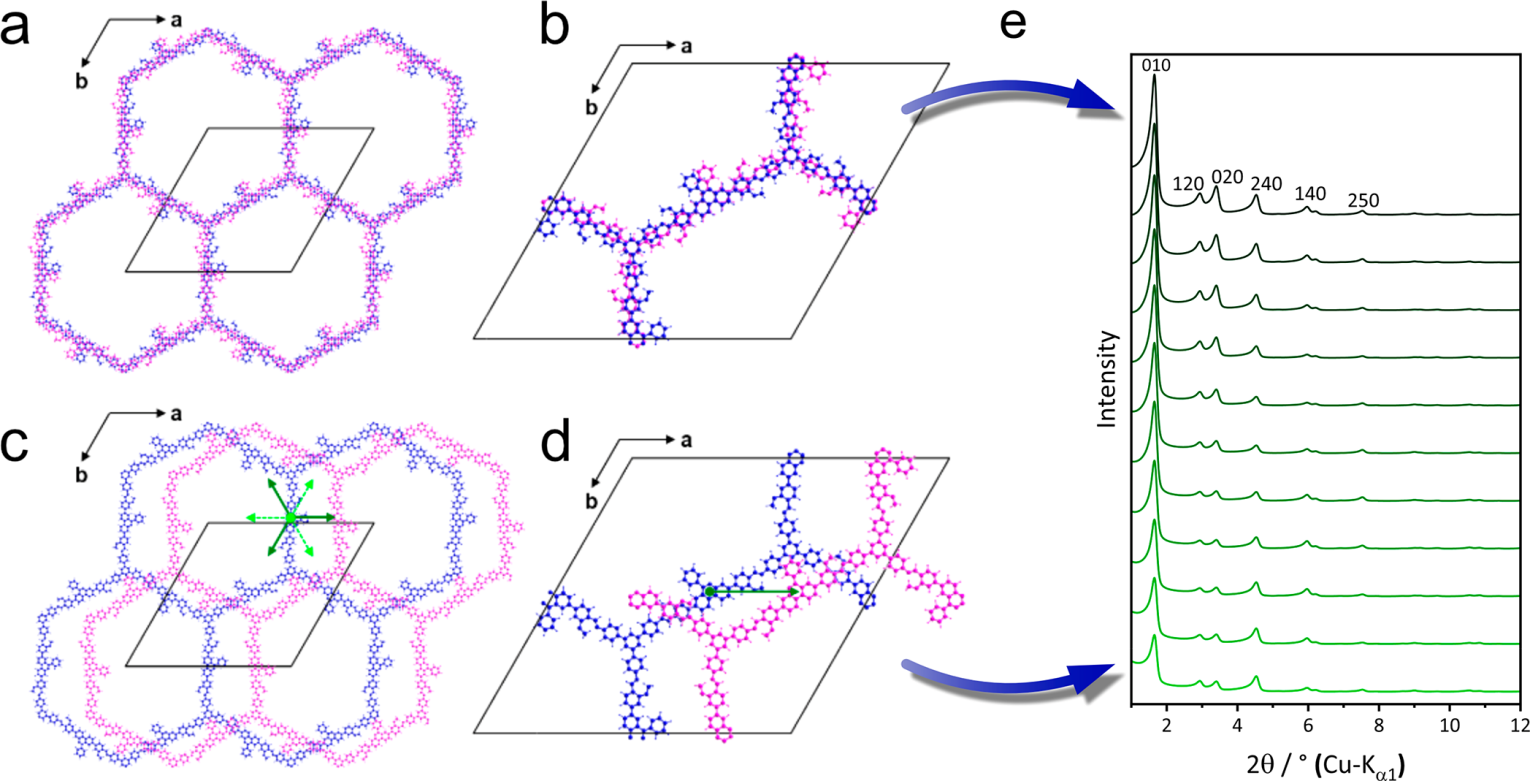
Possible stacking orders in mPP-TAB: (a) overview and (b) detailed view of a nearly eclipsed stacking of A- (magenta) and A̅-type stacked layers; (c) overview and (d) detailed view of a staggered stacking of A- (magenta) and B̅-type stacked layers. The possible stacking vectors for a staggered stacking are indicated by green and light green arrows. The constitution of the A̅-type layer is identical to the A-type layer, but the A̅-type layer is flipped vertically by 180° around the 110 vector axis. (e) Corresponding simulated XRPD patterns of mPP-TAB on moving from an eclipsed (top) to a staggered (bottom) conformation. Note the decreasing intensity of the reflections.

XRPD measurements show that all six COFs of the series are highly crystalline (Figure 1a and b). However, it is noteworthy that mPP-TAB and dPP-TAB of the smaller isoreticular series as well as dPP-TAPB of the larger isoreticular series display a seemingly higher crystallinity, supported by the large number of reflections in the XRPD patterns of up to 13, which is unusual for COFs. In comparison, the other COFs show less reflections, and especially in the case of mPP-TAPB, substantial peak broadening is observed. In addition, a distinct 00l stacking reflection is absent for the latter ones. These noticeable differences in the apparent crystallinity in the isoreticular series can be attributed to inherent differences in stacking order between these COFs. The stacking orders can generally be described as an alternating AA̅-type stacking, where the A- (Figure 2a, b, blue) and A̅-type layers (Figure 2a, b, magenta) exhibit identical constitution, but the A̅-type layer is flipped vertically by 180° around the 110 vector axis with respect to the A-type layer (Figure 2b). These layers can either occupy an almost eclipsed AA̅-type stacking, with a slight random stacking disorder,^41^ or a staggered AB̅-type stacking, where layer B̅ is shifted by a certain stacking vector, indicated by green and light green arrows in Figure 2c and d.

To gain further insights into the different effects of structure and stacking for this isoreticular set of COFs, we performed systematic DIFFaX-like simulations of the XRPD patterns for mPP-TAB as an exemplary COF,^42,43^ using different stacking and faulting scenarios (Figure 2e and Figure S13). This simulation shows the transition of a nearly eclipsed AA̅-type structure into an ordered, staggered AB̅-type stacking as the most likely scenario. The shift within the *ab*-plane by applying the stacking vector was incrementally increased to 12 Å. This leads to a decrease of the peak intensities of the 010, 1̅20, 020, and 1̅30 reflections, as observed in the measured pattern with low apparent crystallinity. To evaluate this proposed structure model, we performed Rietveld refinements on the collected powder patterns. The layers were modeled as flat layers without any torsions, as including this would have led to an overextension of the parameter space. Symmetry-adapted spherical harmonics were applied to the peak widths in order to compensate the peak broadening caused by intra- and interlayer disorder.

The refinement of the XRPD patterns collected for mPP-TAB, dPP-TAB, and dPP-TAPB (Figures S9, S11, and S12, respectively) with the nearly eclipsed AA̅-type stacked model led to a good fit of the measured XRPD pattern and to a satisfying agreement factor (*R*_wp_ = 4.58%, 3.83%, and 3.73%, respectively). For PP-TAB, PP-TAPB, and mPP-TAPB (Figures S7, S8, and S10, respectively), which exhibit a staggered AB̅-type stacking, a 12-layer supercell had to be used for refinement to ensure a satisfying fit (*R*_wp_ = 1.44%, 2.23%, and 2.56%, respectively). Within the refined supercell all layers show a staggered arrangement with a stacking vector of around 12 Å. The results, summarized in Table 1, also show reduced *a* and *b* lattice parameters of the COFs in staggered stacking compared to the isoreticular, eclipsed COFs, which indicates additional distortion by layer buckling and twisting, introduced by the additional degrees of freedom in the staggered arrangement.

**Table 1:** Comparison of Crystal Structure Features and Pore Size Distribution of Investigated COFs^a^

COF	*a* and *b*/Å	stacking type	layer buckling	*S*_BET_/m^2^ g^–1^	pore size/nm
PP-TAB	56.62(9)	staggered AB̅	yes	1063	4.2
mPP-TAB	58.83(4)	nearly eclipsed AA̅	no	1823	4.8
dPP-TAB	58.49(7)	nearly eclipsed AA̅	no	1467	4.8
PP-TAPB	69.29(6)	staggered AB̅	yes	1032	4.8
mPP-TAPB	69.97(4)	staggered AB̅	yes	631	4.9
dPP-TAPB	73.17(6)	nearly eclipsed AA̅	no	1670	5.8

a All patterns were refined in *P*1 symmetry, due to stacking faults present. Lattice parameters were constrained to pseudo-hexagonal values, *a* = *b*, α = β = 90°, and γ = 120 °C.

Scanning electron microscopy (SEM) and transmission electron microscopy (TEM) imaging were performed for all samples to gain further insights into the morphology of the COF particles (Figures S56–S67). SEM images show a cauliflower-like morphology for AB̅-type stacked PP-TAB, PP-TAPB, and mPP-TAPB due to intergrown, curved sheets forming smooth spheres. AA̅-type stacked mPP-TAB, dPP-TAB, and dPP-TAPB show a comparatively rugged morphology exposing crystal facets, which points to the growth of ordered, plate-like crystallites.

TEM images show high crystallinity for all samples, with domain sizes ranging from 20 to 300 nm. The domains seem to be generally larger for AA̅-type stacked mPP-TAB, dPP-TAB, and dPP-TAPB COFs. This can be rationalized by their increased stacking interactions favoring larger domains.

### Computational Structure Investigations

To gain additional insights into the structural arrangement of the nearly eclipsed COFs, we performed a computational study to acquire an in-depth understanding of the stacking of phenylphenanthridine units, the role of the methoxy groups, and the imine configurations. We first analyzed the interaction of two isolated molecular building units. We performed DFT calculations using TURBOMOLE^44^ on the PBE-D3/def2-TZVPP-level^45−47^ and geometry optimizations using the DL-find^48^ interfaced via CHEMSHELL^49^ for the combination of molecules as well as for their isolated counterparts (for details see SI S10).

**Figure 3:**
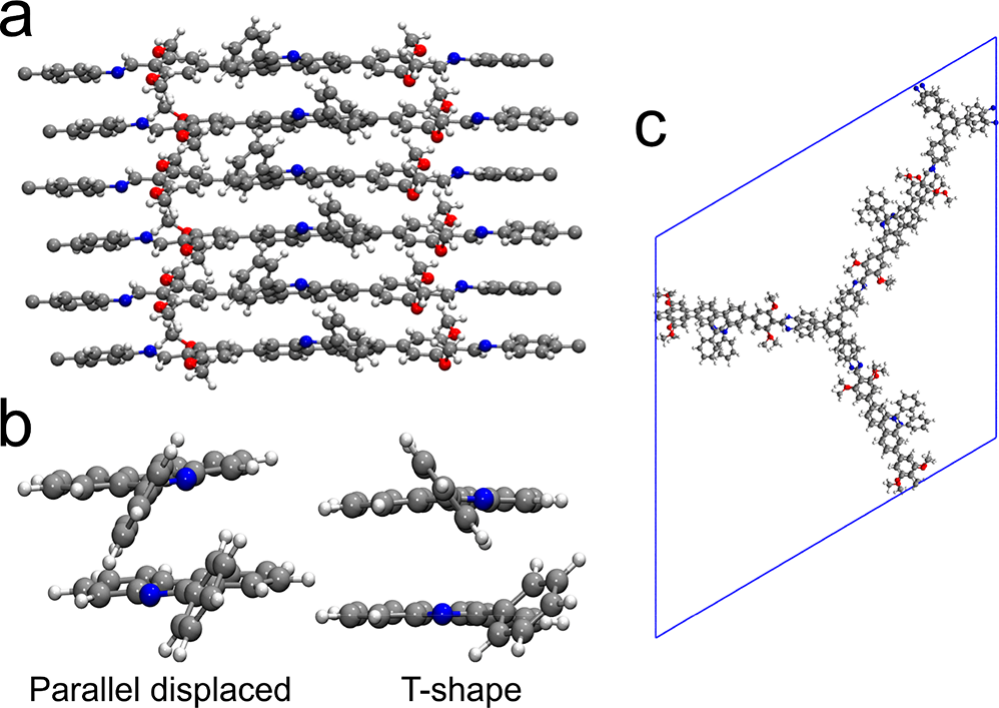
(a) Exemplary periodic combinations of blocks modeling a multilayer dPP-TAB COF pore wall. (b) Parallel-displaced and T-shape stacking arrangement of phenylphenanthridine units. (c) Final unit cell of dPP-TAB after geometry optimization with GPW-DFT.

In a second step, we investigated different arrangements of building units by constructing cut-out blocks of the COF structure to model single pore walls (Figure 3a). All our solid-state density functional calculations were performed using the quick step (QS) Gaussian and plane waves (GPW) approach as implemented in CP2K^50^ with periodic boundary conditions, the PBE-D3^46,47^ functional with GTH-pseudopotentials,^51^ and a TZV2P-GTH^52^ basis set.

For the structures that we identified as the most promising, we then performed a complete unit-cell treatment, leading to an optimal arrangement of these large-pore COFs (Figure 3c).

To investigate the stacking behavior of the phenylphenanthridine units, all four possible stacking variants (Scheme S5) were initially optimized on a nonperiodic molecular level (Table S4). Further optimization in a periodic combination of blocks modeled the restricted in-plane movement of a multilayer COF pore wall.

The results of the nonperiodic model and the comparison of relative energies of isolated components of the COF pore wall simulation (Table S9) find the s10 variant to be the optimal stacking configuration of the phenylphenanthridine units. Assuming a rotation of the protruding phenyl groups, parallel-displaced or T-stacked configurations (Figure 3b), which are both commonly found as stacking motifs in proteins,^53,54^ are possible and, due to their similar energies, likely to coexist in the structure.

For judging the influence of methoxy groups on the imine linkage stacking and orientation, we again initially optimized a nonperiodic model to gain insight into the overall properties stemming from the components. The *in vacuo* analysis suggests the alternating arrangement of imine linkers as most probable (Table S5, Figure S46).

To gain insight in the underlying electrostatic interactions, molecular electrostatic potential maps were calculated for all isolated components. These maps explain the alternating arrangement by the possibility to arrange differently charged areas in a more favorable manner to minimize repulsion (Figure S48).

We could also observe that the difference in interaction energy between the two orientations is reduced by added methoxy groups. Furthermore, we performed calculations with periodic combinations of blocks to model a multilayer COF pore wall. The interaction energies show, in analogy to the isolated model, alternatingly oriented imine groups to be energetically favorable, as they minimize repulsion between the layers (Figure S49).

However, for the periodic case that restricts the molecules in their in-plane movement, the difference between alternating and parallel orientations is smaller (Δ*E* = −12.7 kcal/mol) and becomes even smaller when methoxy groups are added to the linking site (Δ*E* = −1.13 kcal/mol).

This structural effect results from the reduction of the inherent dipole moment of the imine linker by the adjacent methoxy groups,^39^ which “softens” the repulsion between the imine groups, especially in a parallel orientation. This is seen in both models and also in the periodic simulation of multilayer COF pore walls, taking different phenylphenanthridine orientations into account (Figure S50 and Tables S6–S8). Furthermore, we observed that structures with the phenylphenanthridine oriented on different sides become significantly less favorable when methoxy groups are added (Table S7), which is not observed in the methoxy-free case (lowest Δ*E* = 13.01 kcal/mol). As a result, the amount of possible orientations can be expected to be halved in the methoxy case, which indicates an increase in structural order.

In building blocks with only one methoxy group we found that same-side, parallel-oriented methoxy groups, as those shown in Figure S51, are always energetically favorable compared to alternatingly oriented ones (Table S8). We conclude that the methoxy groups add stability due to their steric interactions with each other, which is in most cases also indicated by their apparent out-of-plane orientation (Figure S49).

This behavior was further studied by a complete unit cell treatment of the dPP-TAB COF. Taking into account the influence of the dispersion interactions, we performed cell optimizations of the whole unit cell for methoxy groups ordered in parallel and antiparallel arrangements (Tables S10–S12). It was observed that systematic out-of-plane orientations of the methoxy groups are energetically favorable for every utilized method but even more so the systematic parallel arrangement of the groups (Table S14 and Figure S53). With these results on the configuration the complete unit cell of eclipsed stacked dPP-TAB and PP-TAB with parallel and alternating imine linkers, as well as s10 and s11 phenylphenanthridine units, was optimized using the periodic GFN-xTB implementation in CP2K for a broad analysis as well as GPW-DFT calculations for the most probable structures (Table S13).

For dPP-TAB, the obtained values for the lattice parameters (*a* = *b* = 58.198 Å, *c* = 7.25 Å for alternating imines and s10, unit cell displayed in Figure 3c) are in good agreement with the ones refined from the powder patterns. Calculating PP-TAB as a nearly eclipsed structure results in strikingly similar values for lattice parameters to dPP-TAB (Table S13). These differ by almost 2 Å from the smaller lattice parameters found experimentally, probably due to a lack of buckling of the structure in the DFT calculations. However, if the eclipsed stacking is abandoned by shifting layers toward the actually staggered AB̅ stacking, increased buckling is observed (Figure S55), and the lattice parameters *a* and *b* are reduced to 56.709 Å, matching the values found experimentally.

### Porosity Investigations

The porosity and Brunauer–Emmett–Teller surface area (*S*_BET_) of the COFs were characterized by measuring nitrogen sorption isotherms at 77 K to further confirm our structural models via their pore sizes. *S*_BET_ of all the COFs was determined in a suitable pressure region (*P*/*P*_0_ = 0.1–0.3), and pore size distributions (PSDs) were calculated from the adsorption branch by quenched solid density functional theory (QSDFT) based on the carbon model for cylindrical pores and are summarized in Table 1.^55,56^ All isoreticular COFs are porous with typical type IV isotherms^57^ and show, except for PP-TAB, a distinct hysteresis (Figure 1c and d). The calculated *S*_BET_ values for the COFs show a spread ranging from moderately porous (631 m^2^ g^–1^ for mPP-TAPB) to highly porous (1823 m^2^ g^–1^ for mPP-TAB). The COFs crystallizing in a staggered conformation show overall significantly lower *S*_BET_ values compared to the nearly eclipsed staggered COFs. This can be rationalized by an increase in porosity by both well-defined layer stacking^32^ and a decrease in effective pore size through the staggered arrangement.

Calculated PSDs show a pore size of 4.8 nm for methoxy-containing mPP-TAB and dPP-TAB of the smaller set of isoreticular COFs (Figure 1e). This finding is in good agreement with our structure model assuming a nearly eclipsed AA̅-type stacking. However, the remaining, methoxy-free COF of this set, PP-TAB, shows a smaller pore size of 4.2 nm, corresponding to a reduced pore size, in line with our structure model of a staggered AB̅-type stacking. In the series of larger isoreticular COFs, dPP-TAPB shows a large pore size of 5.8 nm, while for PP-TAPB and mPP-TAPB a smaller pore size of 4.8 and 4.9 nm is found, respectively (Figure 1f). Also in this series, the observed pore sizes correspond well with our structure models of a nearly eclipsed AA̅-type stacking for dPP-TAPB and a staggered AB̅-type stacking for PP-TAPB and mPP-TAPB.

For that, we crosschecked the obtained experimental results and our interpretation by using an additional geometric pore analysis. Here we determined the pore diameter by calculating the biggest sphere able to fit through the spanned pore arrangement, by constructing a pore system using the experimentally obtained structures for both COF systems using the PP- and dPP-COFs, respectively (for details see SI S9). The obtained results summarized in Table S3 perfectly follow the trend of the obtained values from the adsorption measurements showing the increase in pore size from 4.05 nm for PP-TAB to 5.90 nm for dPP-TAPB and, thus, further support our interpretation of favored stacking motifs.

Overall, the structure refinements and the observed PSDs as well as the complementary calculations regarding the stacking interactions between the layers confirm the existence of significantly different stacking behaviors for the isoreticular, large-pore COF series. A clear trend between favorable interlayer interactions and increasing pore size due to specific stacking behavior of the COFs is evident. While PP-based COFs with the least favorable stacking interactions prefer a staggered AB̅-type stacking motif, resulting in reduced permanent pore size, the dPP-based COFs, experiencing the most favorable stacking interactions through the methoxy “anchors”, crystallize in a nearly eclipsed AA̅-type layer stacking. The intermediate mPP-based COF pair switches its stacking mode from eclipsed AA̅-type for TAB toward staggered AB̅-type for TAPB with increasing linker length, illustrating the tendency toward staggered conformations as a function of increasing node-to-node distance. While staggered, offset stacking conformations are usually designed by steric tuning,^58−61^ the few large-pore COFs previously reported also show a tendency toward this.^18−23^ Our results show that this stacking tendency of large-pore COFs is closely linked to the interlayer interactions. By prompting layer shifts and therefore reducing the effective pore size and layer overlap, targeted properties such as porosity and charge transfer can be negatively affected.^32^ However, upon introducing additional interlayer interactions, the stacking behavior can be judiciously altered and effective pore sizes can be maximized.

**Figure 4:**
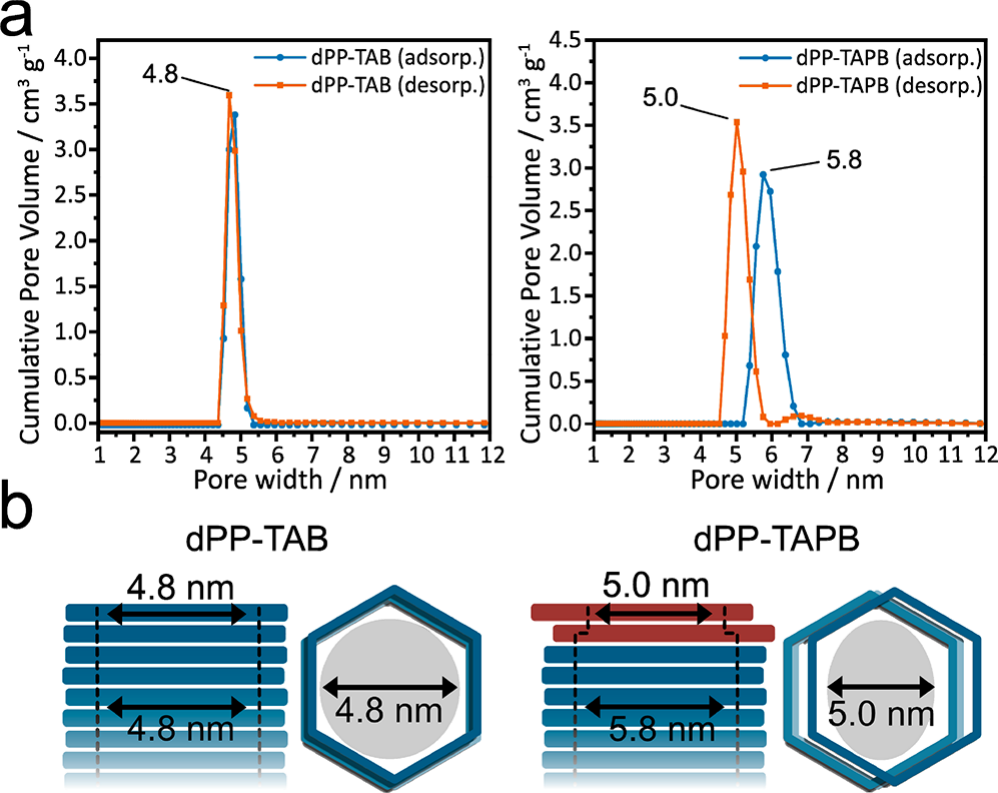
(a) Calculated PSDs of dPP-TAB (left) and dPP-TAPB (right) for the adsorption and desorption branches, calculated with quenched solid density functional theory (QSDFT) based on the carbon model for cylindrical pores.^55,56^ (b) Schematic representation of neckless open-pore system of dPP-TAB (left) and occurrence of a pore neck by an offset of the outer layers for dPP-TAPB.

Additionally, dPP-TAPB exhibits an adsorption behavior that so far has no precedence in COFs. It shows a very broad sorption hysteresis (Figure 1d), which can be ascribed to a pore-blocking phenomenon as typically found for ink-bottle-shaped pores.^62,63^ Compared to dPP-TAB, dPP-TAPB has a disparate PSD for the adsorption and desorption branch of the isotherm (Figure 4a). While the adsorption branch shows the expected delayed pore condensation for the major pore size of 5.8 nm, in good agreement with the structural model, the desorption branch signals that evaporation of the capillary condensate is dominated by a neck size of 5.0 nm diameter. However, the complete lack of pores with 5.0 nm diameter in the PSD of the adsorption branch indicates a very thin neck size, which is not recognized as individual pores. The tendency of the other isoreticular COF systems with weaker interlayer interactions to form staggered structures with pore sizes of around 5 nm thus suggests a potential offset of just one or very few “labile” outer layers into a staggered arrangement, creating this smaller neck size (Figure 4b).

### Thermal Stability and Phase Transition of Large Pore Phenylphenanthridine COFs

Observing different stacking modes for otherwise isoreticular and chemically extremely similar COF systems, we further investigated how these different stacking modes and interlayer interactions influence the thermal behavior of large-pore COFs. As previously shown by Evans et al.,^37,64^ COFs can undergo structural change and loss of crystallinity well below the degradation temperature measured by thermogravimetric analysis (TGA). An increasing buckling of the COF layers at higher temperatures was found, leading to amorphization of the network. In addition, a trend of lower thermal stability with increasing pore size and pore functionalization was identified. It was postulated that larger and more mobile pendant groups, similar to the methoxy groups used here, can already cause significant disruption of interlayer interactions in 2D COF sheets as thermal energy is added, thus resulting in lower thermal stability. Taking into account that thermal stability can become a limiting factor in the application of COFs,^65,66^ insights into this effect, especially at large pore sizes, is a key prerequisite.

### Thermal Behavior

**Figure 5:**
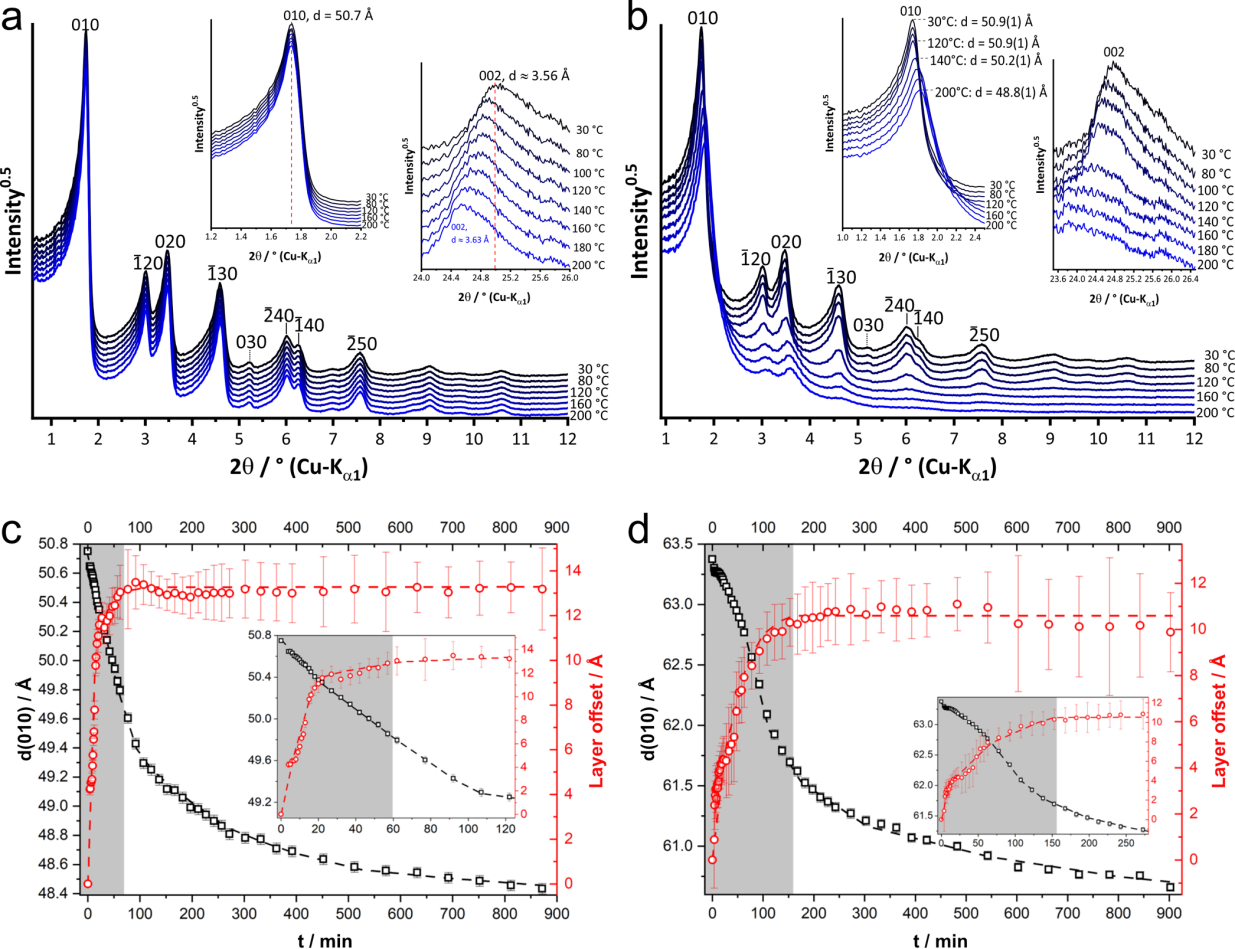
(a) Temperature-dependent *in situ* XRPD patterns of dPP-TAB including selected reflection indices as a close-up of the low 2θ region, close-up of the 010 reflections, and close-up of the 002 reflection. (b) Temperature-dependent *in situ* XRPD patterns of mPP-TAB including selected reflection indices as a close-up of the low 2θ region, close-up of the 010 reflections, and close-up of the 002 reflection. Evolution of the 010 lattice plane distance (black squares) and of the mean layer offset (red cycles) while holding (c) mPP-TAB at 140 °C and (d) dPP-TAPB at 120 °C. The insets show the initial evolution of the 010 lattice plane distance and the mean layer offset. The gray-shaded areas indicate the period in which the mean layer offset significantly increases.

In TGA measurements, all COFs in our series show a typical, high thermal stability of up to 400 °C before any mass loss is detected (Figure S7). The XRPD patterns for the *in situ* heating experiments were consecutively collected at 30, 80, 100, 120, 140, 160, 180, and 200 °C (Figure 5a and b and Figures S14–S17), with a delay period of 4 h prior to each measurement in order to ensure equilibration. The final patterns at 200 °C for all COFs were further refined according to the models described above (Figures S7–S12).

For the staggered AB̅-type stacked COFs, PP-TAB, PP-TAPB, and mPP-TAPB, a minor reduction in crystallinity and peak broadening as well as a slight shift of the *hk*0 reflections was observed (Figures S14–S16). These observations stem from additional layer buckling at higher temperatures as previously described by Evans et al.^37,38^ This additional layer buckling is irreversible and remains upon cooling of the samples.

dPP-TAB COF shows almost no change in its reflections upon heating to 200 °C except for a slight shift of the 002 stacking reflection toward lower diffraction angles (Figure 4a). This shift can be ascribed to a small thermal expansion between the layers of 0.08 Å, which was also found to be reversible upon cooling.

mPP-TAB and dPP-TAPB show the most significant changes upon heating (Figure 4b and Figure S17). Up to a transition temperature of 140 °C for mPP-TAB and 120 °C for dPP-TAPB, both COFs still behave comparable to the temperature-stable dPP-TAB. A slight shift of the 002 stacking reflection indicates thermal expansion, while no buckling of the layers is evident. At transition temperature, the basal 002 reflections show a substantial broadening and completely merge with the background at higher temperatures. Simultaneously, an initial shift of the *hk*0 reflections and their broadening take place. Continuing stepwise further up to 200 °C, the final shift of the 010 reflections corresponds to a reduction of the *a* and *b* lattice parameters by 2.1 and 3.3 Å, respectively. As for PP-TAB, PP-TAPB, and mPP-TAPB, this shift is a result of temperature-induced layer buckling. However, the observed peak broadening that ultimately results in the disappearance of higher angle *hk*0 reflections strongly resembles our simulations for the transition of a nearly eclipsed stacked AA̅-type structure to an ordered, staggered AB̅-type stacking (Figure 2e). The results of the Rietveld refinement of the final ht-XRPD patterns at 200 °C are summarized in Table S2. They show for ht-mPP-TAB and ht-dPP-TAPB, which have visible similarity to the patterns of staggered PP-TAB and PP-TAPB, a staggered AB̅-type stacking mode with satisfying agreement factors (*R*_wp_ = 4.39% and 3.09%, respectively).

To confirm this observation of a temperature-induced phase transition from an eclipsed toward a staggered type stacking for mPP-TAB and dPP-TAPB, corresponding sorption experiments were performed. For this purpose, the samples were consecutively activated under vacuum at the given temperatures for 4 h before measurement. For mPP-TAPB no major changes below the transition temperature of 140 °C occur, in analogy to the XRPD heating experiments (Figure S29). Reaching the transition temperature, the previously steep uptake at around *P*/*P*_0_ = 0.5 resembling the capillary condensation in 4.8 nm wide pores begins to flatten out. With increasing temperature, the initially broad hysteresis then closes. During the stepwise transition, the *S*_BET_ almost halves from 1823 m^2^ g^–1^ at room temperature to 930 m^2^ g^–1^ after activation at 200 °C. The PSD shows a related, harsh drop in the cumulative pore volume of 4.8 nm and the appearance of a new pore size at 4.0 nm at 140 °C (Figure S29). With higher temperatures, all pores at 4.8 nm disappear after being transformed into the smaller pores correlating to a staggered AB̅-type stacking of ht-mPP-TAB. Similar behavior is observed for dPP-TAPB, with a decrease of the *S*_BET_ to 920 m^2^ g^–1^ and the disappearance of pores with a 5.8 nm diameter accompanied by a rise of 4.8 nm pores at 120 °C and above (Figure S30). The *S*_BET_ and PSD of the ht-phases are in good accordance with the values found for isoreticular, already at room temperature staggered, PP-TAB and PP-TAPB COFs.

While the structural change is irreversible and permanent upon cooling, exposing the COFs to solvent followed by supercritical (sc)CO_2_ activation could recover the initial high crystallinity, *S*_BET_, and PSD in both cases (Figures S31 and S32). As shown by Sick et al.,^67^ this process can even at low temperatures sufficiently recover interlayer correlation, indicating the near-eclipsed stacking of mPP-TAB and dPP-TAPB is preferred in solution.

While phase changes by interlayer shifting under solvent influences^68,69^ or by removal of structurally incorporated solvent^70^ are known, our heating experiments show a remarkable, temperature-induced phase transition for COFs due to layer mobility, which was to the best of our knowledge never observed before. The structural transition of mPP-TAB and dPP-TAPB from eclipsed into staggered already occurs at rather mild temperatures of 140 and 120 °C, respectively, and is reversible upon contact with solvent. The findings show again correlation between interlayer interactions and pore size, as for the room-temperature isoreticular series. While the relatively smaller-sized pores of dPP-TAB with strong interlayer interactions are unaffected up to 200 °C, the isoreticular mPP-TAB shows structural integrity only up to 140 °C, when layer mobility gets sufficient to transform it into a staggered structure. With the increased pore size of dPP-TAPB, compared to dPP-TAB, the interlayer interactions are again not strong enough to prevent layer displacement above 120 °C. In our case, the increased interlayer interactions of pendant methoxy groups, anchoring the layers in nearly eclipsed stacking, do not follow the general negative influence on thermal stability of other pore functionalization found by Evans et al.^37,64^ and, therefore, offers new insights into the thermal stability of COFs.

### Kinetic Study of Phase Transition

To monitor the development of the observed phase change over time, mPP-TAB and dPP-TAPB were heated above the transition temperature, to 140 and 120 °C, respectively, and the evolution of the diffraction patterns was monitored while holding the samples at constant temperatures. As an example, the XRPD patterns of mPP-TAB are shown in Figure S18. Several minutes after heating at the transition temperature, a decrease in intensity of the 010, 1̅20, 020, and 1̅30 reflections, indicating a shift of the layers toward a staggered AB̅-type stacking, can be observed (Figure S18a). In addition, the 010 reflections shift to higher 2θ angles (Figure S18b), which is attributed to the development of a buckling of the COF layers. These processes continue, even after more than 2 h.

In order to gain further insights into the mechanism of shifting and curving of the layers, the patterns were analyzed by fully weighted Rietveld refinements,^71^ using the previously applied two-layer unit cells with a planar COF conformation. The almost eclipsed AA̅-type stacked structure was used for the patterns collected at 30 °C. For the refinement of the patterns collected at higher temperatures, the space group symmetry was lowered to *P*1 and the upper layer was allowed to shift freely within the *ab*-plane. The absolute value of the shifting vector of the upper lattice plane was used as a measure of transition from nearly eclipsed toward staggered AB̅-type stacking. The *a* and *b* lattice parameters were constrained and refined as well in order to get a measure on the development of the COF-layer buckling. Symmetry-adapted spherical harmonics were applied to the peak widths in order to compensate the peak broadening caused by intra- and interlayer disorder. All refinements were carried out in a serial way, which means that for the refinement of a pattern collected at a time *t*_*i*_ the refined parameters of the preceding pattern collected at *t*_*i*–1_ were used. The parameters were released iteratively. At first, the lattice parameter was refined, and then the layer shift and finally the peak profile parameters were released. The resulting evolution of the mean layer offset and the curving of the COF layers of mPP-TAB and dPP-TAPB are given in Figure 5c and d.

For both mPP-TAB (Figure 5c) and dPP-TAPB (d) the curving and the shifting of the COF layers start immediately. The absence of an induction period in the evolution of the layer offset and the shrinking of the *a* and *b* lattice parameters indicate that buckling of the layers does not require any initial shift and shifting of the layers does not require any initial buckling. The development of the layer offset (Figure 5, gray-shaded areas) is always faster than curving of the layers. As a consequence, both processes seem to proceed independently of each other. However, the lack of temperature-induced layer curving in dPP-TAB even at 200 °C, which also does not show any signs of layer shifting, indicates that a slight initial layer shift might be necessary for the occurrence of layer buckling under thermal stress. In mPP-TAB the shifting and curving of the layers occur faster than in dPP-TAPB, which is attributed to the higher transition temperature. During the development of the COF-layer offset in mPP-TAB, the layer shifts by 13 ± 2 Å, whereas in the case of dPP-TAPB the layer shifts by 11 ± 2 Å. The layer shift found is in good agreement with the staggered structure model obtained for ht-mPP-TAB and ht-dPP-TAPB at 200 °C.

### Computational Investigations of Stacking Potential Energy Landscapes

**Figure 6:**
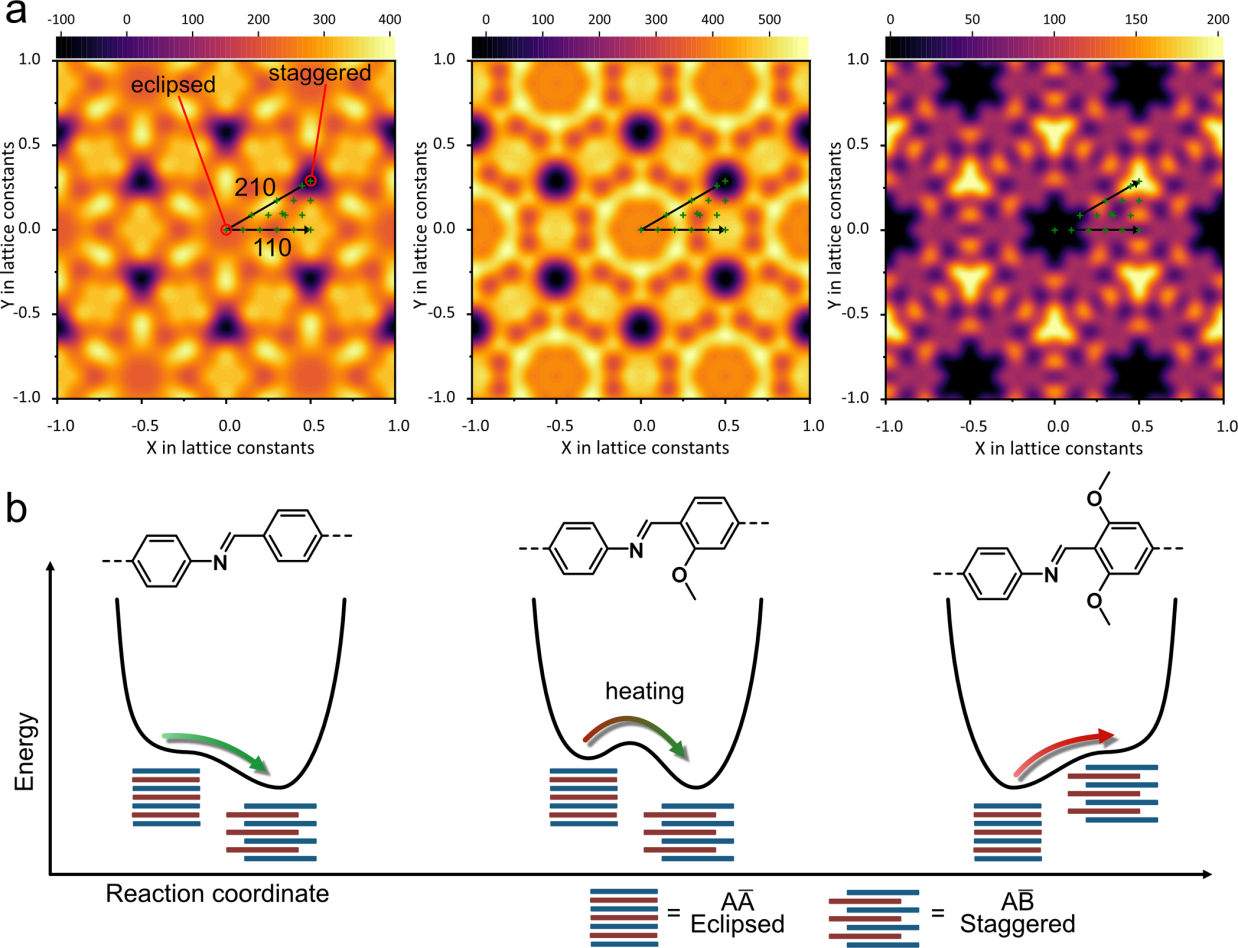
(a) Potential energy landscapes assuming a shift of the A̅-type layer in comparison to the A-type layer by a certain vector (Figure 2c) of PP-TAB, mPP-TAB, and dPP-TAB (left to right); green crosses show surface data points used for interpolation, black arrows indicate the respective direction in space, and total eclipsed and staggered positions are marked in the left graph. (b) Schematic potential energy changes as a function of the stacking mode of PP-TAB, mPP-TAB, and dPP-TAB.

To gain further insights into the origin of the phase transition, displacement potential energy landscapes for PP-TAB, mPP-TAB, and dPP-TAB were calculated (Figure 6a). Investigations were performed for the relative displacement of two layers per unit cell constructed by using the knowledge of the structural analysis, to resemble the most stable molecular arrangements for eclipsed stacking. We created the initial structure by arranging both layers with overlapping centers of mass as the displacement origin. Starting from this arrangement we displaced the upper layer along the marked points in Figure 6a, sampling the asymmetric unit of a *D*_6_-symmetric 2D unit cell, i.e., one triangle necessary to create the full hexagonal unit cell as shown in (Figure S54). For every displacement we performed geometry and cell optimizations using GFN-xTB^72^ as implemented in CP2K, as it showed qualitatively similar behavior when compared with single-point energies for the optimized cells using GPW-DFT (Figure S52). The obtained energy values were then interpolated by a plane-wave expansion with *D*_6_ symmetry. The obtained potential energy landscapes show the distribution in the potential energy landscapes assuming a shift of the A̅-type layer in comparison to the A-type layer by a certain vector as shown in Figure 2c, with the totally eclipsed stacking as the origin in the center of the landscapes at (0,0).

For dPP-TAB, with the strongest interlayer interactions, only a small, clearly favorable energy window corresponding to a nearly eclipsed stacking is found.^31^ PP-TAB COF shows the energetic minima consistent with staggered stacking. The mPP-TAB displays a similar landscape to PP-TAB with the energetic minima predicting staggered configurations. However, in this case a small energy barrier between the nearly eclipsed and staggered configurations was found. These observations are rationalized in a simplified qualitative scheme (Figure 6b). PP-TAB, with the least pronounced interlayer interactions, shows a single clear minimum for a staggered conformation, while dPP-TAB, with the strongest interlayer interactions, shows a minimum for the nearly eclipsed arrangement. The experimentally observed stacking arrangements match these findings. However, mPP-TAB displays two significant minima, a local one for the eclipsed structure and a global one for the staggered structure with a small, yet significant energy barrier dividing both. While mPP-TAB crystallizes with nearly eclipsed stacking when synthesized at a temperature of 120 °C, just below the transition temperature and in the presence of a solvent, heating can provide the necessary activation energy to transform it into the more stable staggered arrangement. Of note, the reversibility after exposure to solvent indicates that in the solvated form the nearly eclipsed configuration is favored and only becomes metastable after solvent removal.

## Conclusion

We successfully developed six novel large-pore COFs with a maximum pore size of 5.8 nm, which is among the largest pore sizes reported for COFs to date. More importantly, methoxy groups in phenylphenanthridine building blocks were identified to act as anchors that eliminate the tendency toward slipped layer stacking in large-pore COFs, which typically leads to notoriously reduced effective pore sizes and surface areas. XRPD results together with theoretical modeling indicate mPP-TAB, dPP-TAB, and dPP-TAPB to adopt a nearly eclipsed stacking mode with maximized pore sizes, attributed to enhanced interlayer interaction in these COFs, mediated by the methoxy groups. While increasing interlayer interactions positively influence the thermal stability of the COFs, we further demonstrate, for the first time, a temperature-induced phase transition to the staggered stacking polytype, enabled by high layer mobility even at temperatures as low as 120 °C. The slow transition could be readily followed by *in situ* XRPD measurements, shedding light on the two independent processes involved, layer shifting and buckling. Altered porosity and crystallinity for the high-temperature phases are persistent but were easily reversed by solvent exposure, suggesting solvent-induced changes of the stacking mode to be an additional degree of freedom in influencing the layer stacking besides the observed thermally induced phase transitions. Considering the fact that the synthesis of large-pore COFs with >5 nm pore size remains a challenging task in the COF chemistry, we anticipate that the findings presented herein will aid the rational design of COFs with targeted pore size, layer registry, and thermal stability for specific applications.

## Supporting Information Available

Description of materials and methods, synthesis of monomers and COFs, FT-IR spectra, XRPD patterns and Rietveld refinements, temperature-dependent XRPD plots, nitrogen gas sorption isotherms, BET plots, pore size distribution plots, liquid and solid state NMR spectra, TGA analysis, technical details of geometry-based pore size calculation and theoretical investigation, SEM and TEM images (PDF)
