# Supplementary material for: Interlayer Interactions as Design Tool for Large-Pore COFs
Source: J Am Chem Soc. 2021 Sep 8;143(38):15711–22. doi: 10.1021/jacs.1c06518 (PMC8485322; doi:10.1021/jacs.1c06518)
Supplement: Supplementary file 1 — ja1c06518_si_001.pdf [file ja1c06518_si_001.pdf]

Supporting Information

Interlayer Interactions as Design Tool for Large-Pore COFs

Sebastian T. Emmerling, Robin Schuldt, Sebastian Bette, Liang Yao, Robert E. Dinnebier, Johannes Kästner, and Bettina V. Lotsch*

Table of Contents

Experimental Procedures 2

S1 Materials and Methods 2

Results and Discussion 3

S2 Experimental Section 3

S3 FT-IR spectroscopy 6

S4 XRPD measurements and refinements 9

S5 Gas Sorption Experiments 19

S6 Solid State NMR 27

S7 Thermogravimetric Analysis 28

S8 Liquid State NMR 29

S9. Geometry-Based Pore Size Calculation 35

S10. Technical Details for the Theoretical Investigations 37

S11 SEM and TEM 54

References 58

Experimental Procedures

S1 Materials and Methods

Chemicals. All starting materials and reagents were obtained from commercial suppliers and used without further purification. All solvents, unless otherwise specified, were obtained from Acros Organics, and used without further purification. 1,3,5-Tris[4-amino(1,1-biphenyl-4-yl)]benzene was prepared according to a literature procedure.[1]

X-Ray Powder Diffraction. XRPD patterns were collected at 30 °C, 80 C, 100 °C, 120 °C, 140 °C, 160 °C, 180 °C, and 200 °C on a laboratory powder diffractometer in Debye-Scherrer geometry (Stadi P-diffractometer (Stoe), Cu-$K_{\alpha 1}$ radiation from primary Ge(111)-Johann-type monochromator, triple array of Mythen 1 K detectors (Dectris)). The sample was sealed in a 1.0 mm diameter borosilicate glass capillary (Hilgenberg glass no. 0140), which were spun during the measurements. Heating was performed using a capillary heater Oxford Cryostream 700 (Oxford Cryosystems), applying a delay period of 4 h prior to each measurement in order to ensure equilibration. Each powder pattern was collected in a 2θ range from 0 ° to 110 ° with a total scan time of 3 hours. The program TOPAS 6.0 was used for the data analyses. All details on the refinements are described below.

The XRPD measurements of the heating kinetics were performed at constant temperatures using the same device. After reaching a constant temperature, powder patterns were collected in a 2θ range from 0 ° to 110 ° with a total scan time of one minute. During the first 10 minutes, powder patterns were collected every minute, during the subsequent 10 minutes every two minutes, during the subsequent 40 minutes every 5 minutes, during the subsequent 210 minutes every 15 minutes, during the subsequent 120 minutes every 30 minutes, and during the subsequent 600 minutes every 60 minutes.

FT-IR Spectroscopy. Infrared spectra were measured in attenuated total reflection (ATR) geometry on a PerkinElmer UATR Two equipped with a diamond crystal. All spectra were background corrected.

Liquid State Nuclear Magnetic Resonance. All liquid state nuclear magnetic resonance (NMR) measurements were performed on a JEOL ECZ 400S 400 MHz spectrometer (magnetic field 9.4 T). ^{1}H, ^{13}C and ^{15}N measurements were performed in 5 mm NMR tubes using deuterium field lock. NMR spectra were internally calibrated to the corresponding solvent signal.[2] Abbreviations for multiplicities: s (singlet), bs (broad singlet), d (dublet), t (triplet), q (quartet), hept (heptet), m (multiplet).

Solid State Nuclear Magnetic Resonance. Solid-state NMR (ssNMR) was recorded on a Bruker Avance III 400 MHz spectrometer (magnetic field 9.4 T). Samples were packed in 4 mm ZrO_2 rotors, which were spun at 12-14 kHz in a Bruker WVT BL4 double resonance MAS probe. Chemical shift was referenced relative to tetramethylsilane (^{13}C). A standard cross-polarization sequence with a 2 ms ramped contact pulse was used for ^{13}C and a total of 4096-8192 scans were routinely accumulated. All spectra were background corrected.

Sorption Measurements. Sorption measurements were performed on a Quantachrome Instruments Autosorb iQ 3 with nitrogen at 77 K. The pore size distribution (PSD) was determined from nitrogen adsorption isotherms using the QSDFT (cylindrical pores, adsorption branch) or QSDFT (cylindrical pores, equilibrium) kernel in carbon for nitrogen at 77 K implemented in the ASiQwin software v 3.01. Samples were activated under high vacuum at room temperature for 12 h before measurement unless stated otherwise.

Mass Spectrometry. Electrospray ionization (ESI) mass spectrometry was performed on a Thermo Finnigan LTQ FT in the positive and negative mode. Samples were dissolved in an acetonitrile/water mixture.

Thermal Analysis. Thermogravimetric analysis was performed on a *NETZSCH STA 449 F3 Jupiter*. Measurements were carried out with 5 mg of sample in an Al_2O_3 crucibles under Helium protective gas flow in a temperature range between 40 to 1000 °C and a heating rate of 10 K/min.

Scanning Electron Microscopy. SEM SE (secondaryelectron) detector images were obtained on a Zeiss Merlin.

Transmission Electron Microscopy. TEM was performed with a Philips CM30 ST at 300kV (LaB6cathode). Samples were prepared dry onto a holey carbon/copper grid.

Results and Discussion

S2 Experimental Section

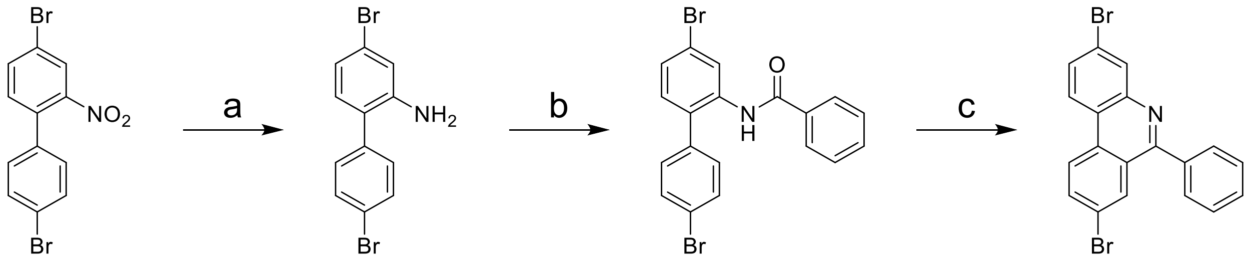

1 **2** **3** **4**

Scheme S1. Synthesis route to the phenylphenanthridine precurser for the COF building block.

Synthesis of 4,4'-dibromo-[1,1'-biphenyl]-2-amine (2). 4,4'-dibromo-[1,1'-biphenyl]-2-amine (**2**) was synthesized according to a modified literature procedure.[3] Aqueous HCl (25 mL, 37%) was added to a solution of **1** (5.28 g, 14.8 mmol) in 50 mL ethanol. Tin powder (3.52 g, 29.6 mmol) was added to the stirring solution portionwise and the mixture was heated to reflux overnight. After cooling, the mixture was poured into ice water (300 mL) under stirring, the precipitate was collected by filtration and washed with water. The washed precipitate was dried under vacuum to give the product **2** that was used for the next step without further purification (4.50 g, 13.8 mmol, 93%). ^{1}H NMR (400 MHz, DMSO-d_6) δ 7.62 (d, J = 8.2 Hz, 2H), 7.34 (d, J = 8.2 Hz, 2H), 6.95 (d, J = 1.6 Hz, 2H), 6.89 (d, J = 8.1 Hz, 1H), 6.75 (dd, J = 8.1, 2.0 Hz, 1H), 5.15 (s, 2H). ^{13}C NMR (101 MHz, DMSO-d_6) δ 147.0, 137.7, 131.7, 131.7, 130.8, 123.5, 121.5, 120.3, 118.9, 117.1. HRMS (ESI) exact mass calculated for [M+H]+ ($C_{12}H_9Br_2N$) requires m/z 326.90813, found m/z 326.9069.

Synthesis of *N*-(4,4'-dibromo-[1,1'-biphenyl]-2-yl)benzamide (3). *N*-(4,4'-dibromo-[1,1'-biphenyl]-2-yl)benzamide (**3**) was synthesized according to a modified literature procedure.[3] Under argon **2** (2.00 g, 6.14 mmol) and triethylamine (1.00 mL, 7.37 mml) were dissolved in dry CH_2Cl_2 (30 mL) and benzoyl chloride (0.79 mL, 6.75 mmol) was added dropwise. The reaction mixture was stirred at room temperature overnight, then ethanol (5 mL) was added. After 1 h, the solution was poured into water (50 mL) and extracted with CH_2Cl_2. The combined organic layers were dried over anhydrous Na_2SO_4 and evaporated under reduced pressure. The resulting crude product was purified by recrystallization from CH_2Cl_2/*n*-hexane to obtain **3** (2.26 g, 5.24 mmol, 85%). ^{1}H NMR (400 MHz, DMSO-d_6) δ 10.01 (s, 1H), 7.79 (s, 1H), 7.75 (dd, J = 8.2, 1.5 Hz, 2H), 7.62 – 7.52 (m, 4H), 7.48 (t, J = 7.4 Hz, 2H), 7.38 (d, J = 8.5 Hz, 2H), 7.35 (d, J = 8.3 Hz, 1H). ^{13}C NMR (101 MHz, DMSO-d_6) δ 165.8, 137.4, 136.5, 136.0, 134.1, 131.9, 131.7, 131.3, 130.6, 130.5, 129.5, 128.4, 127.5, 121.0, 120.4. HRMS (ESI) exact mass calculated for [M+H]+ ($C_{19}H_{13}Br_2NO$) requires m/z 430.93434, found m/z 430.9318.

Synthesis of 3,8-dibromo-6-phenylphenanthridine (4). 3,8-dibromo-6-phenylphen-anthridine (**4**) was synthesized according to a modified literature method.[4] Under argon a Biotage microwave vial was charged with **3** (1.00 g, 2.34 mmol), 2-chloropyridine (0.27 mL, 2.81 mmol) and dry CH_2Cl_2 (10 mL). Trifluormethansulfonic anhydride (0.43 mL, 2.57 mmol) was added dropwise at 0°C. After 5 minutes, the solution was warmed to room temperature, the vial was capped and under microwave irradiation, the solution was heated to 140 °C for 30 minutes. After the solution was cooled to room temperature, triethylamine (0.65 mL, 4.68 mmol) was added dropwise to neutralize the trifluormethanesulfonate salts. All volatiles were removed under reduced pressure and the residue was purified by flash column chromatography (SiO_2, *n*-hexane/ CH_2Cl_2) to obtain **4** (0.90 g, 2.17 mmol, 93%). ^{1}H NMR (400 MHz, $CDCl_3$) δ 8.47 (d, J = 8.9 Hz, 1H), 8.40 (d, J = 2.1 Hz, 1H), 8.38 (d, J = 8.8 Hz, 1H), 8.24 (d, J = 2.1 Hz, 1H), 7.93 (dd, J = 8.8, 2.0 Hz, 1H), 7.76 (dd, J = 8.8, 2.1 Hz, 1H), 7.70 (dd, J = 7.3, 2.1 Hz, 1H), 7.63 – 7.52 (m, 3H). ^{13}C NMR (101 MHz, $CDCl_3$) δ 161.3, 144.7, 138.8, 134.4, 133.0, 132.0, 131.4, 130.7, 129.8, 129.4, 128.9, 126.6, 124.1, 123.4, 123.1, 122.1, 121.8. HRMS (ESI) exact mass calculated for [M+H]+ ($C_{19}H_{11}Br_2N$) requires m/z 412.92378, found m/z 412.9230.

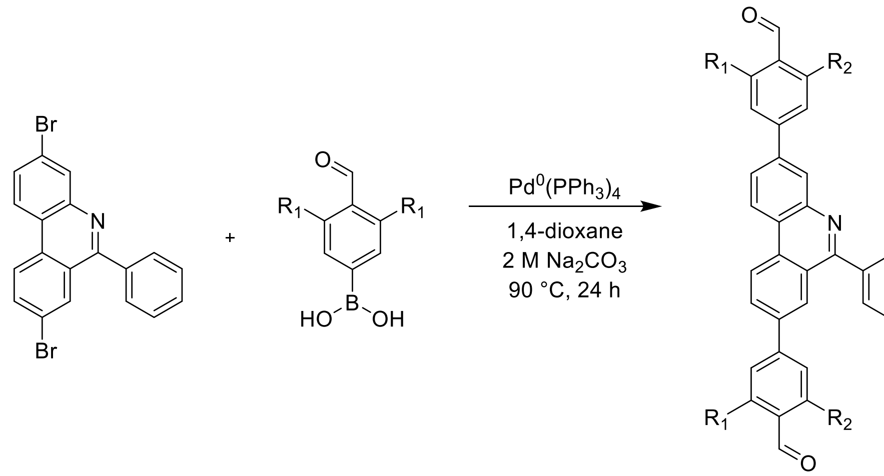

R_1, R_2 = H for **PP**
R_1 = OMe, R_2 = H for m**PP**
R_1, R_2 = OMe for **dPP**

Scheme 2. Synthesis of phenylphenanthridine based COF building blocks **PP**, **mPP**, and **dPP** starting from precurser **4**.

Synthesis of 4,4'-(6-phenylphenanthridine-3,8-diyl)dibenzaldehyde (PP). Under argon, **4** (0.50 g, 1.21 mmol), 4-formylphenylboronic acid (0.73 g, 4.84 mmol) and Tetrakis(triphenylphosphin)palladium(0) (0.07 g, 0.06 mmol) were dissolved in 1,4-dioxane (15 mL) and 2 M aqueous Na_2CO_3 (3.6 mL) was added. After degassing the solution with argon for 30 minutes the mixture was stirred for 24 h at 90 °C. After cooling to room temperature, water was added and the mixture extracted with $CHCl_3$. The combined organic layers were dried over anhydrous Na_2SO_4 and evaporated under reduced pressure. The solid residue was purified by flash column chromatography (SiO_2, *n*-hexane/$CHCl_3$) to obtain **PP** (0.42 g, 0.90 mmol, 74%).
^1H NMR (400 MHz, $CDCl_3$) δ 10.09 (d, *J* = 9.8 Hz, 2H), 8.84 (d, *J* = 8.6 Hz, 1H), 8.74 (d, *J* = 8.6 Hz, 1H), 8.59 (s, 1H), 8.41 (d, *J* = 1.8 Hz, 1H), 8.18 (dd, *J* = 8.6, 1.8 Hz, 1H), 8.06 – 7.96 (m, 7H), 7.81 (t, *J* = 7.7 Hz, 4H), 7.66 – 7.55 (m, 3H). ^{13}C NMR (101 MHz, $CDCl_3$) δ 192.0, 191.8, 162.2, 146.1, 146.0, 140.5, 139.0, 135.8, 135.8, 133.2, 130.6, 130.2, 129.9, 129.5, 128.9, 128.1, 128.0, 127.8, 126.4, 125.8, 123.5, 123.4, 123.2. IR v_{max}/cm^{-1} 3062, 2818, 2730, 1698, 1602, 1554, 1474, 1367, 1309, 1212, 1171, 966, 807, 703, 517. HRMS (ESI) exact mass calculated for [M+H]+ ($C_{33}H_{21}NO_2$) requires m/z 464.16058, found m/z 463.1568.

Synthesis of 4,4'-(6-phenylphenanthridine-3,8-diyl)bis(2-methoxybenzaldehyde) (mPP). Under argon, **4** (0.50 g, 1.21 mmol), 4-formyl-3-methoxyphenylboronic acid (0.76 g, 4.24 mmol) and Tetrakis(triphenylphosphin)palladium(0) (0.10 g, 0.09 mmol) were dissolved in 1,4-dioxane (15 mL) and 2 M aqueous Na_2CO_3 (3.6 mL) was added. After degassing the solution with argon for 30 minutes the mixture was stirred for 24 h at 90 °C. After cooling to room temperature, water was added and the mixture extracted with $CHCl_3$. The combined organic layers were dried over anhydrous Na_2SO_4 and evaporated under reduced pressure. The solid residue was purified by flash column chromatography (SiO_2, *n*-hexane/$CHCl_3$) to obtain **mPP** (0.40 g, 0.79 mmol, 63%). ^1H NMR (400 MHz, $CDCl_3$) δ 10.51 (s, 1H), 10.50 (s, 1H), 8.83 (d, *J* = 8.6 Hz, 1H), 8.73 (d, *J* = 8.5 Hz, 1H), 8.39 (s, 1H), 8.19 (d, *J* = 8.4 Hz, 1H), 8.04 (d, *J* = 8.2 Hz, 1H), 7.96 (d, *J* = 8.0 Hz, 1H), 7.93 (d, *J* = 8.0 Hz, 1H), 7.87 – 7.82 (m, 2H), 7.70 – 7.61 (m, 3H), 7.54 (d, *J* = 7.7 Hz, 1H), 7.50 – 7.42 (m, 2H), 7.28 (d, *J* = 8.1 Hz, 1H), 7.20 (s, 1H), 4.05 (s, 3H), 4.00 (s, 3H) ppm. ^{13}C NMR (101 MHz, $CDCl_3$) δ 189.6, 189.4, 162.4, 162.3, 147.6, 133.2, 132.3, 132.2, 132.1, 132.1, 129.5, 129.4, 128.9, 128.7, 128.6, 128.0, 128.0, 126.7, 125.6, 124.3, 123.5, 123.1, 120.1, 119.9, 110.7, 56.0, 55.9 ppm. IR v_{max}/cm^{-1} 3363, 3060, 2874, 1676, 1601, 1571, 1470, 1404, 1185, 1116, 1029, 854, 796, 697, 539. HRMS (ESI) exact mass calculated for [M+H]+ ($C_{35}H_{25}NO_4$) requires m/z 523.17836, found m/z 523.1772.

Synthesis of 4,4'-(6-phenylphenanthridine-3,8-diyl)bis(2,6-dimethoxybenzaldeyde) (dPP). Under argon, **4** (0.50 g, 1.21 mmol), 4-formyl-3,5-dimethoxyphenylboronic acid (0.89 g, 4.24 mmol) and Tetrakis(triphenylphosphin)palladium(0) (0.14 g, 0.12 mmol) were dissolved in 1,4-dioxane (15 mL) and 2 M aqueous Na_2CO_3 (6 mL) was added. After degassing the solution with argon for 30 minutes the mixture was stirred for 24 h at 90 °C. After cooling to room temperature, water was added and the mixture extracted with $CHCl_3$. The combined organic layers were dried over anhydrous Na_2SO_4 and evaporated under reduced pressure. The solid residue was purified by flash column chromatography (SiO_2, *n*-hexane/$CHCl_3$) to obtain **dPP** (0.46 g, 0.79 mmol, 66%). ^1H NMR (400 MHz, $CDCl_3$) δ 10.55 (s, 1H), 10.53 (s, 1H), 8.82 (d, *J* = 8.7 Hz, 1H), 8.73 (d, *J* = 8.6 Hz, 1H), 8.59 (s, 1H), 8.36 (d, *J* = 1.7 Hz, 1H), 8.14 (dd, *J* = 8.5, 1.7 Hz, 1H), 8.00 (dd, *J* = 8.5, 1.8 Hz, 1H), 7.83 (dd, *J* = 7.5, 1.6 Hz, 1H), 7.60 (d, *J* = 7.3 Hz, 3H), 7.00 (s, 2H), 6.79 (s, 2H), 4.01 (s, 6H), 3.96 (s, 6H) ppm. ^{13}C NMR (101 MHz, $CDCl_3$) δ 189.1, 189.0, 162.7, 162.7, 162.3, 147.9, 147.8, 141.0, 139.7, 133.3, 132.3, 132.2, 132.1, 132.0, 130.0, 129.9, 129.5, 128.8, 128.7, 128.6, 127.8, 126.2, 125.7, 123.6, 123.4, 123.1, 113.6, 103.3, 103.2, 56.4, 56.3 ppm. IR v_{max}/cm^{-1} 3356, 3066,

2941, 2848, 2783, 1674, 1599, 1568, 1455, 1399, 1242, 1178, 1125, 927, 807, 707, 576, 509. HRMS (ESI) exact mass calculated for [M+H]+ ($C_{37}H_{29}NO_6$) requires m/z 583.19949, found m/z 583.1984.

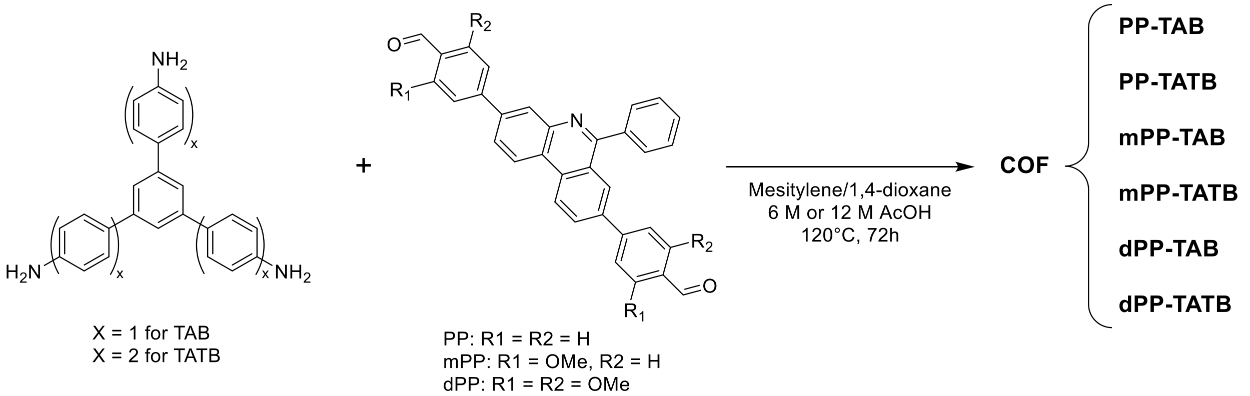

Scheme 3. Synthesis of phenylphenanthridine COFs.

General synthesis of phenylphenanthridine COFs

Into a 5 mL *Biotage* microwave vial, the amine (1.0 eq) and aldehyde (1.5 eq). A mesitlyene/1,4-dioxane mixture was added, followed by 6M or 12M AcOH. The vial was capped and placed in an aluminum heating block that was preheated to 120°C. Under stirring at 500 rpm the mixture was kept at 120°C for 72h, then it was allowed to cool to room temperature. The solid was filtered off, washed with MeOH and then subjected to Soxhlet extraction with MeOH for 16h. The MeOH soaked solid was then activated by scCO2 drying and further under high vacuum for 24h to obtain the COF.

Table S1: Synthesis of phenylphenanthridine COFs

COF	Aldehyde	Amine	Mesitylene/1,4-dioxane, (v/v) / ml	Catalyst	Yield
PP-TAB	PP (20.0 mg, 0.045 mmol)	TAB (10.8 mg, 0.030 mmol)	1/1	6M AcOH (50 µl)	21.1 mg (75 %)
PP-TAPB	PP (30.0 mg, 0.065 mmol)	TAPB (25.0 mg, 0.043 mmol)	1.5/1.5	6M AcOH (75 µl)	32.0 mg (60 %)
mPP-TAB	mPP (23.6 mg, 0.045 mmol)	TAB (10.8 mg, 0.030 mmol)	1.4/0.6	6M AcOH (50 µl)	22.9 mg (71 %)
mPP-TAPB	mPP (23.6 mg, 0.045 mmol)	TAPB (18.7 mg, 0.030 mmol)	1/1	6M AcOH (50 µl)	27.3 mg (69 %)
dPP-TAB	dPP (26.3 mg, 0.045 mmol)	TAB (10.8 mg, 0.030 mmol)	0.7/0.3	12M AcOH (15 µl)	26.6 mg (76 %)
dPP-TAPB	dPP (40.1 mg, 0.069 mmol)	TAPB (26.6 mg, 0.046 mmol)	1.5/0.5	6M AcOH (50 µl)	43.3 mg (67 %)

S3 FT-IR spectroscopy

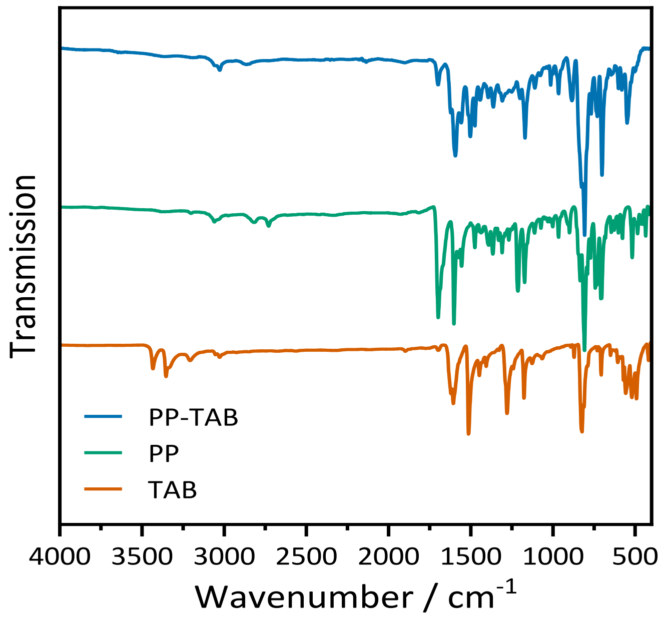

Figure S1. FT-IR comparison of **PP-TAB** and the starting materials **PP** and **TAB**.

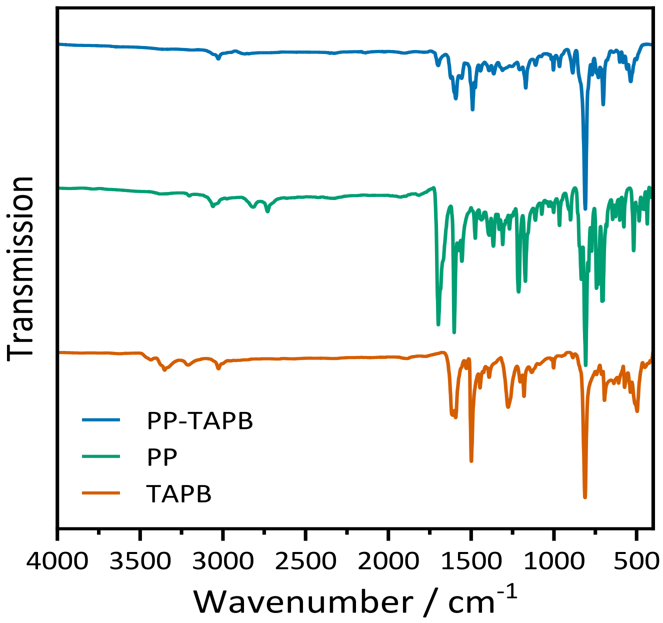

Figure S2. FT-IR comparison of **PP-TAPB** and the starting materials **PP** and **TAPB**.

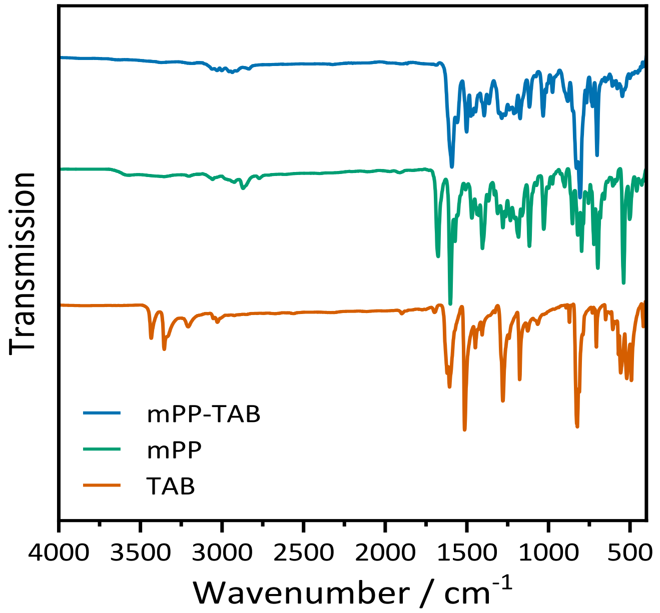

Figure S3. FT-IR comparison of **mPP-TAB** and the starting materials **mPP** and **TAB**.

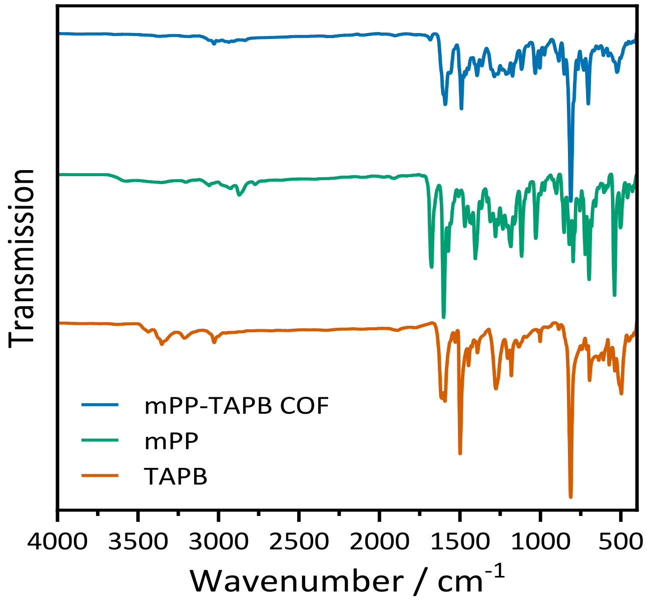

Figure S4. FT-IR comparison of **mPP-TAPB** and the starting materials **mPP** and **TAPB**.

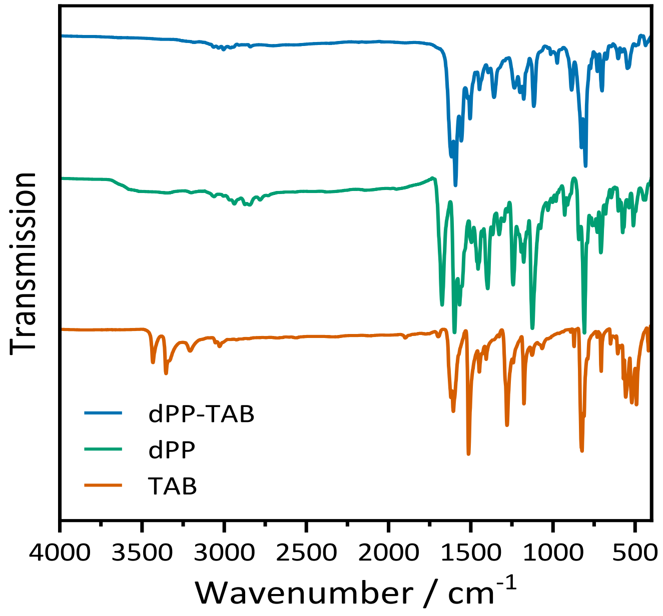

Figure S5. FT-IR comparison of **dPP-TAB** and the starting materials **dPP** and **TAB**.

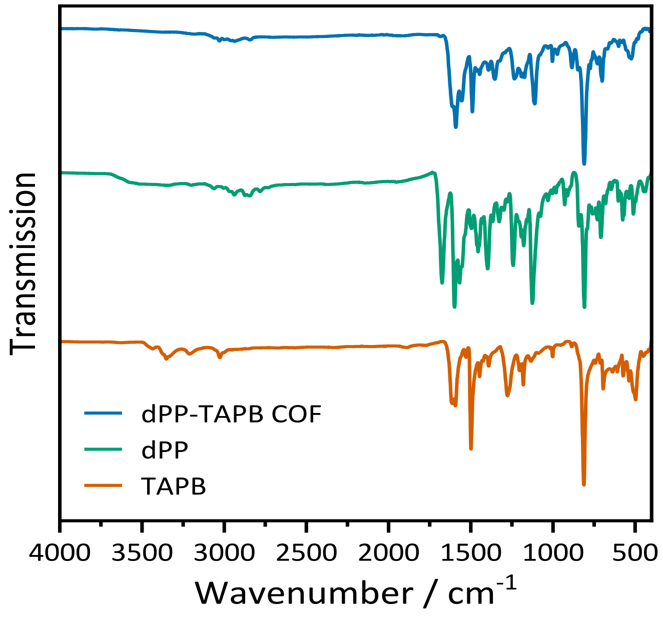

Figure S6. FT-IR comparison of **dPP-TAB** and the starting materials **dPP** and **TAPB**.

S4 XRPD measurements and refinements

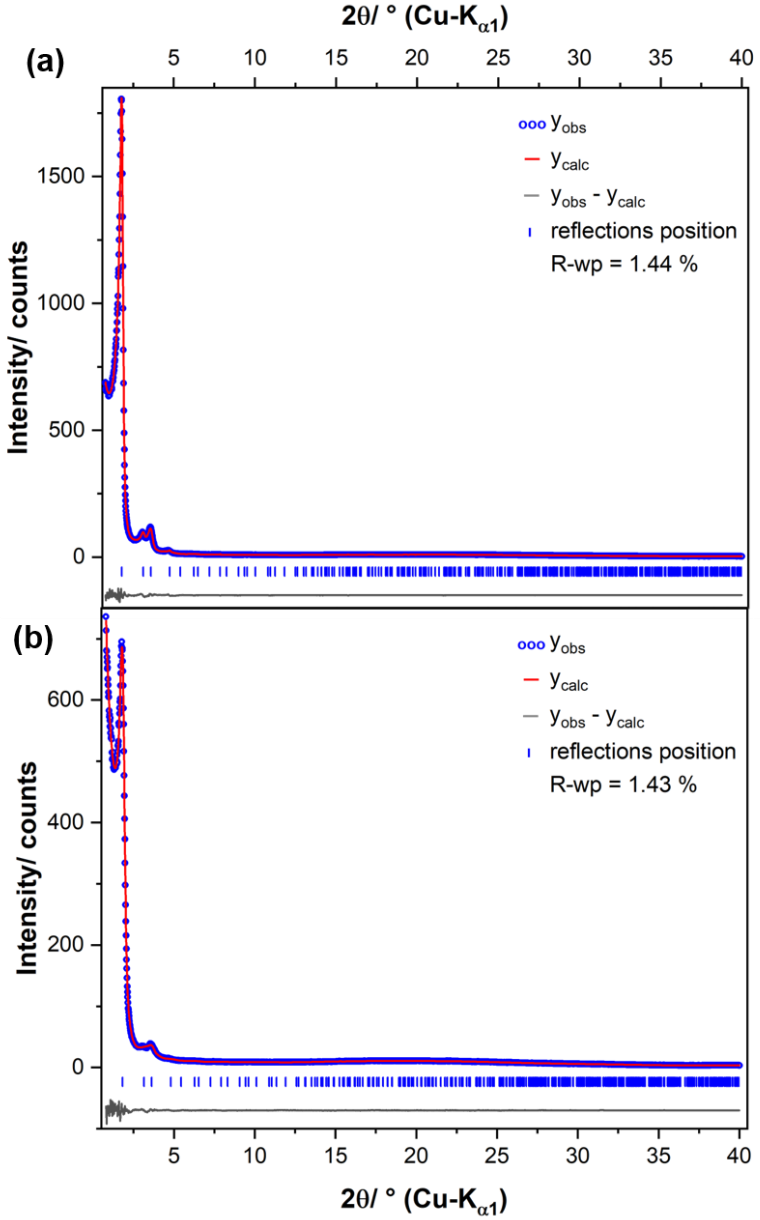

Figure S7. Graphical results of the final Rietveld refinements of the patterns of **PP-TAB.**(a) at 30 °C using a 12 layer supercell model with a disordered A$\overline{B}$-type staggered stacking and (b) at 200 °C using a 12 layer supercell model with a disordered A$\overline{B}$-type staggered stacking with a considerably larger layer offset.

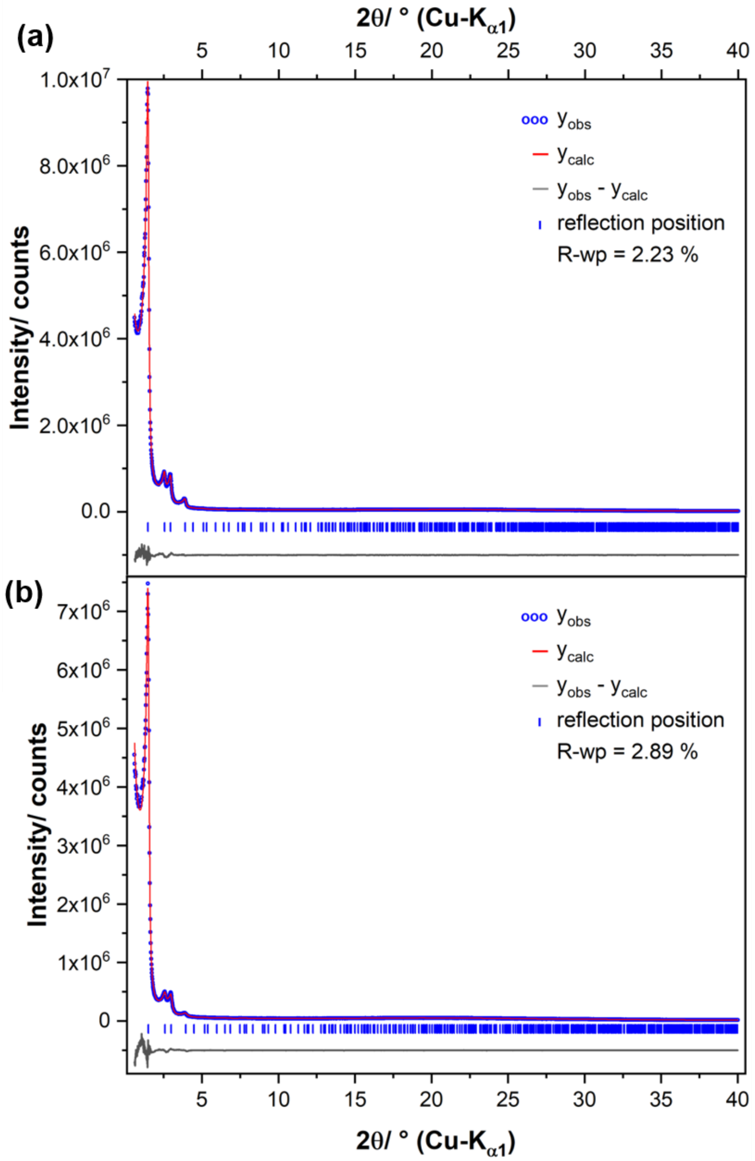

Figure S8. Graphical results of the final Rietveld refinements of the patterns of **PP-TATB** (a) at 30 °C using a 12 layer supercell model with a disordered A$\overline{B}$-type staggered and (b) at 200 °C using a 12 layer supercell model with a disordered A$\overline{B}$-type staggered stacking with a considerably larger layer offset.

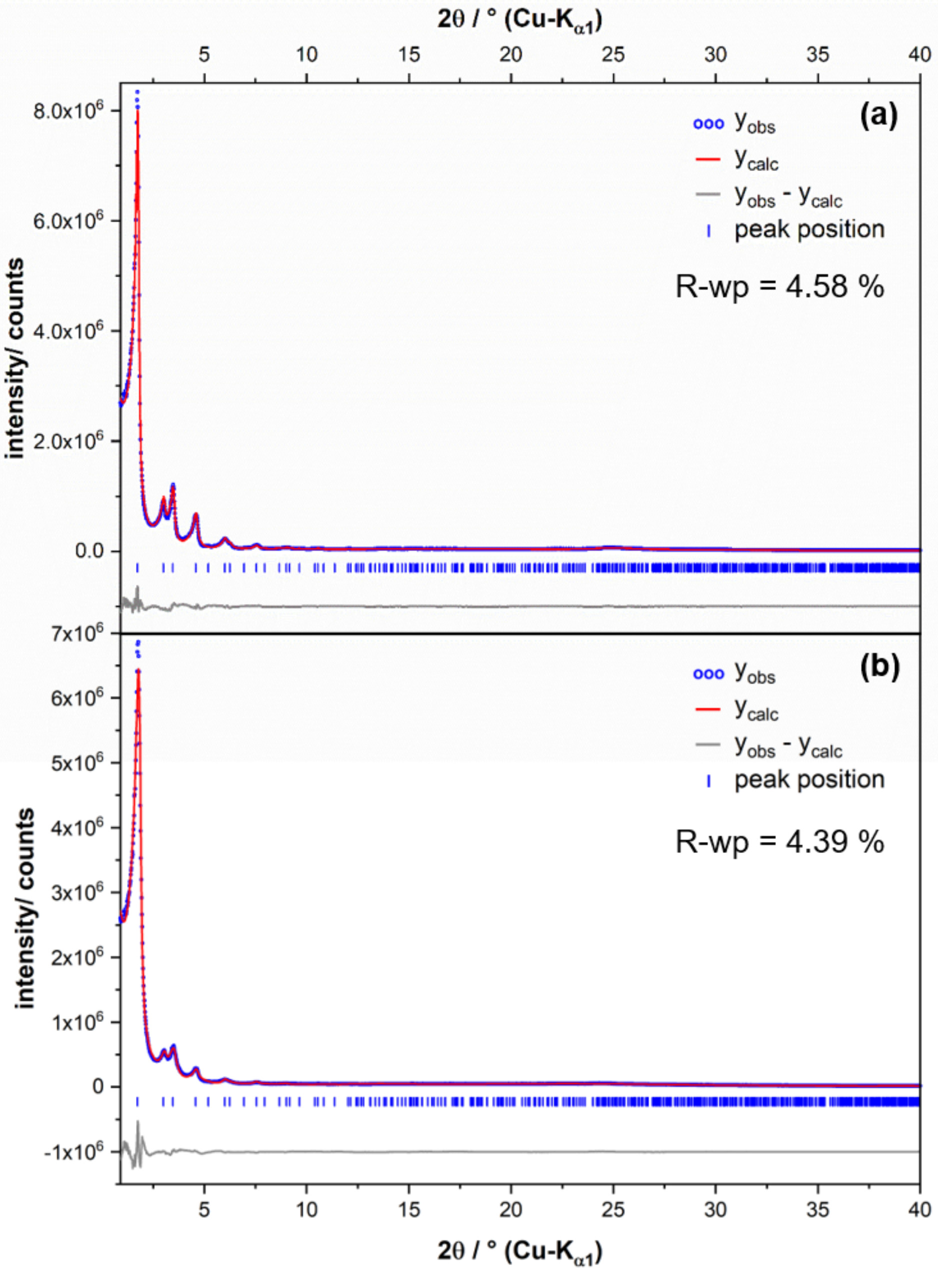

Figure S9. Graphical results of the final Rietveld refinements of the patterns of **mPP-TAB** (a) at 30 °C using an ordered A$\bar{A}$-type eclipsed stacking and (b) at 140 °C using a 4 layer supercell and a slightly disordered A$\bar{B}$-type staggered stacking.

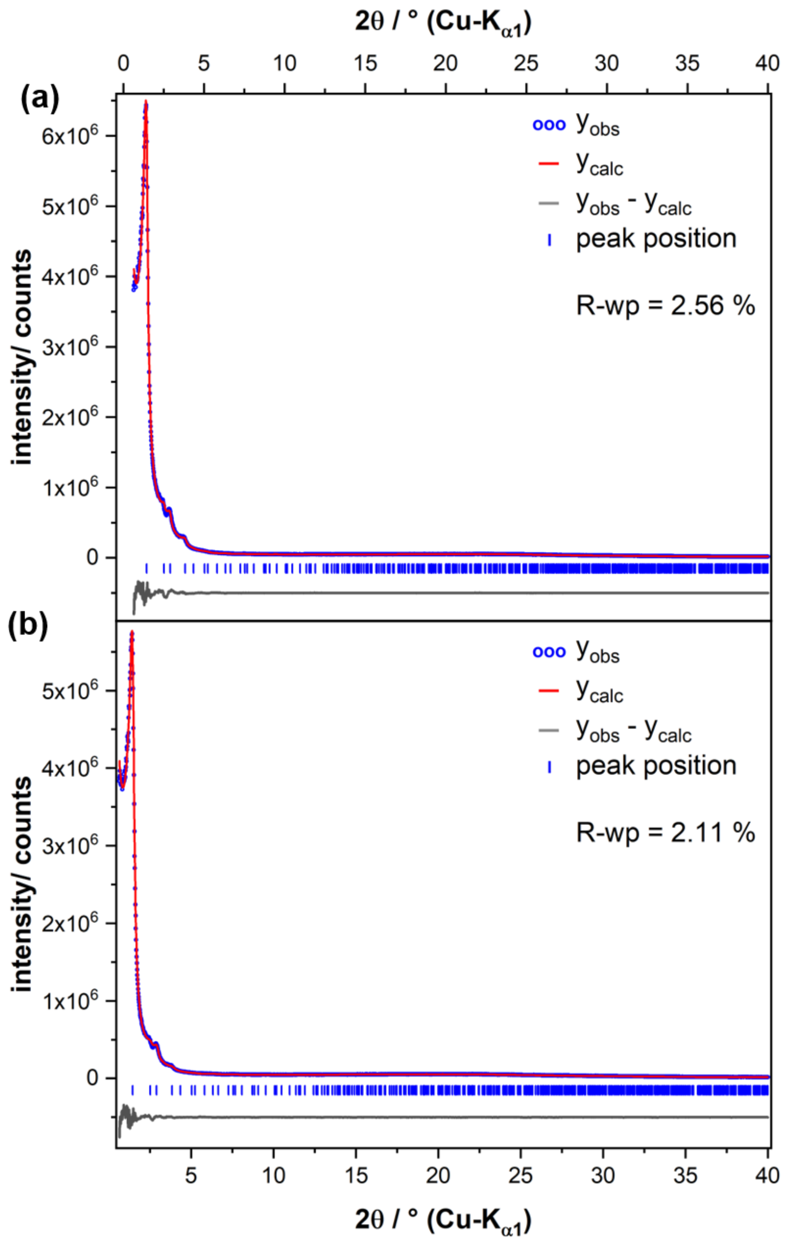

Figure S10. Graphical results of the final Rietveld refinements of the patterns of **mPP-TATB** (a) at 30 °C using a 12 layer supercell model with a disordered A$\overline{B}$-type staggered stacking and (b) at 200 °C using a 12 layer supercell model with a disordered A$\overline{B}$-type staggered stacking larger layer offset.

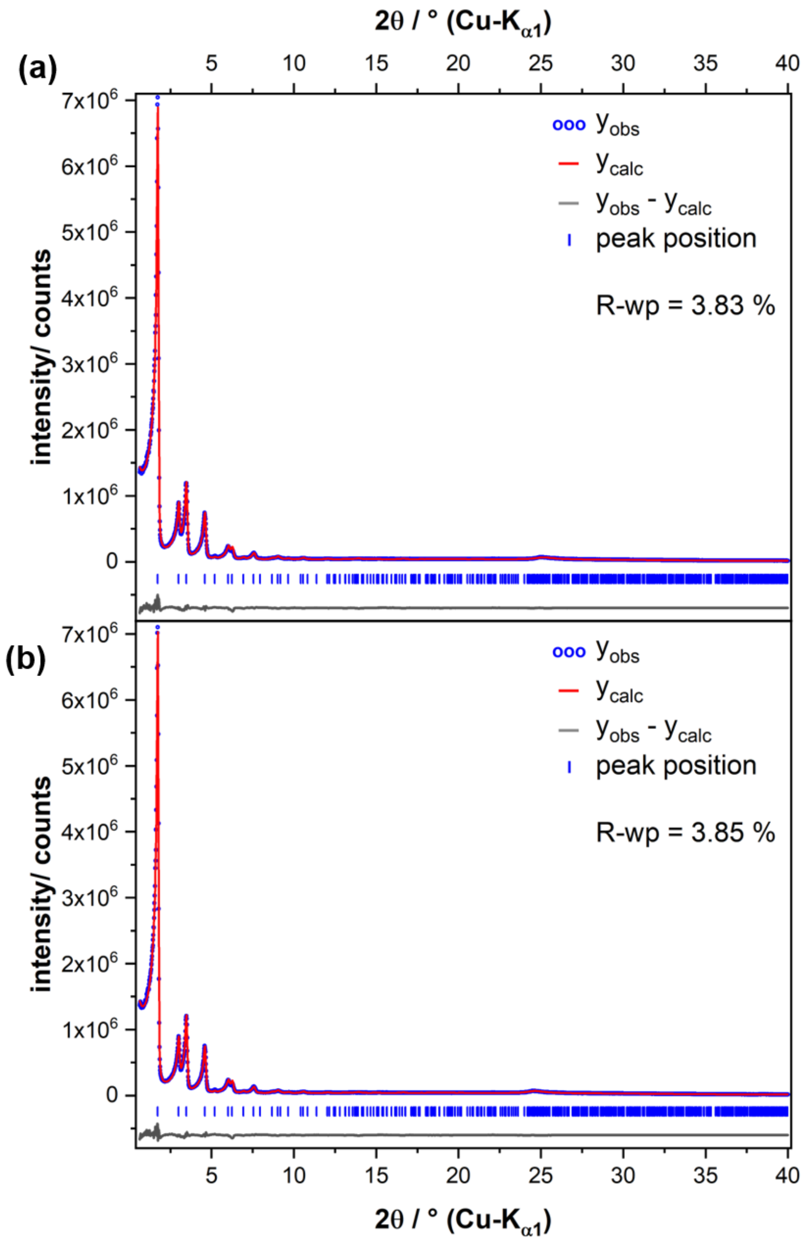

Figure S11. Graphical results of the final Rietveld refinements of the patterns of **dPP-TAB** (a) at 30 °C using a 2-layer model with an ordered A$\bar{A}$-type nearly eclipsed stacking and (b) at 200 °C using a 2-layer model with an ordered A$\bar{A}$-type nearly eclipsed stacking.

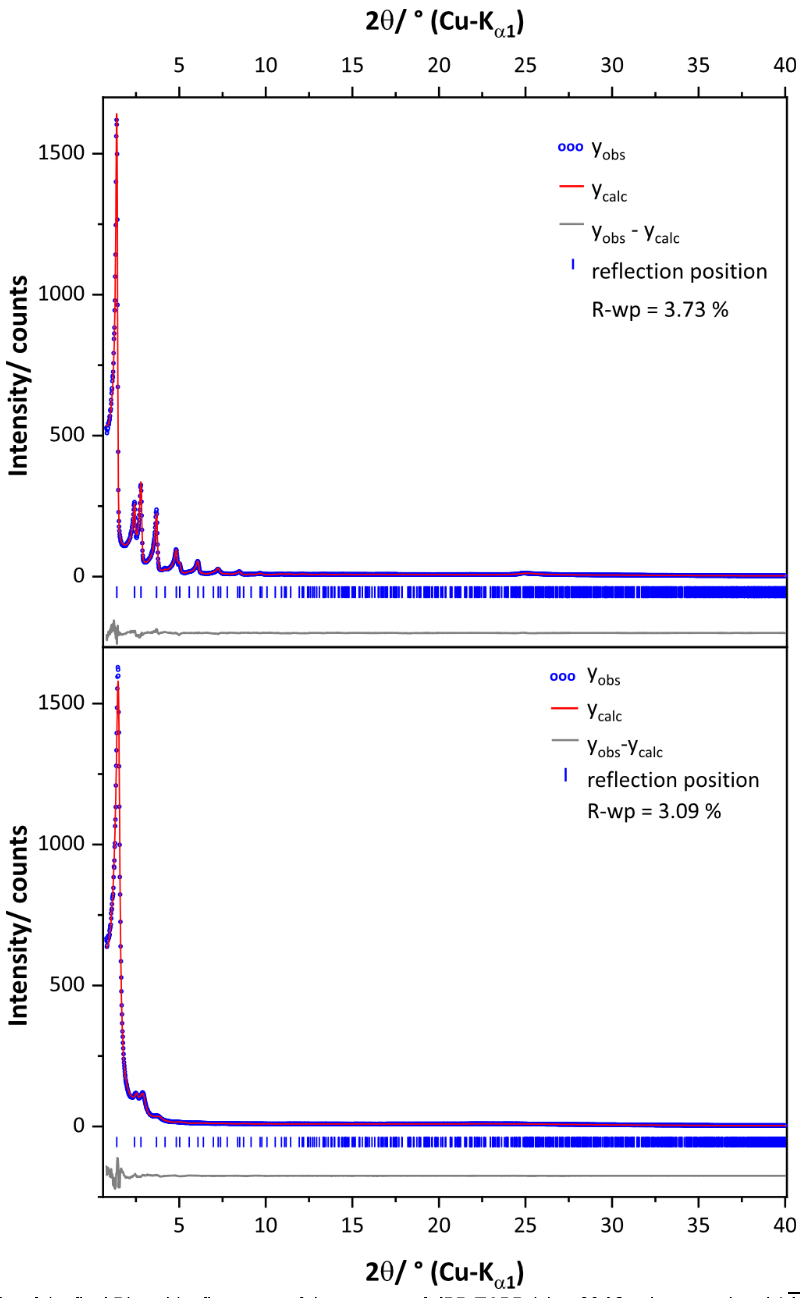

Figure S12. Graphical results of the final Rietveld refinements of the patterns of **dPP-TAPB** (a) at 30 °C using an ordered A$\overline{A}$-type eclipsed stacking and (b) at 140 °C using a 4 layer supercell and a slightly disordered A$\overline{B}$-type staggered stacking.

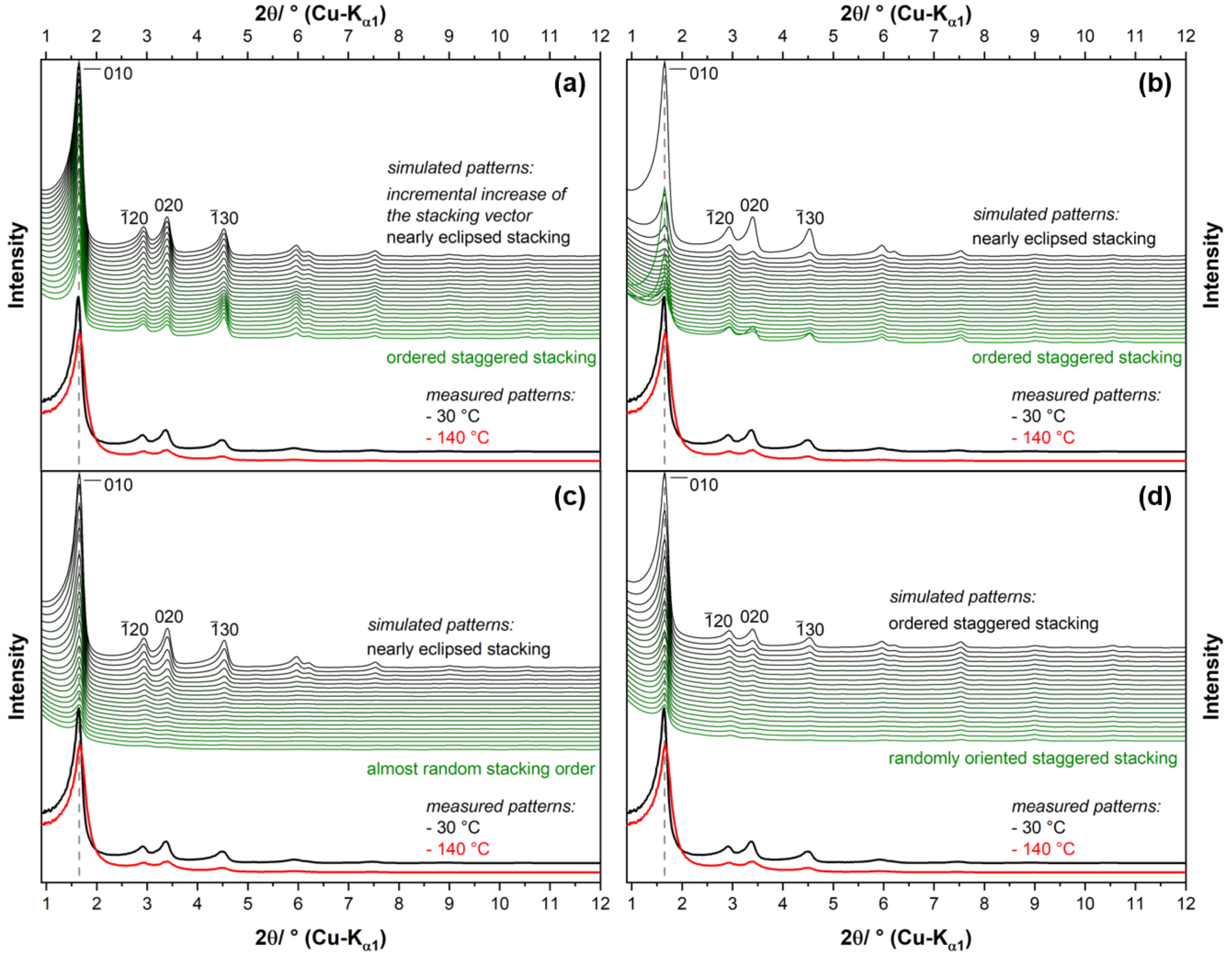

Figure S13. Comparison of simulated and measured diffraction patterns of **mPP-TAB**. (a) incrementally increasing the lengths of the stacking vector starting at 0 Å, i.e. $A\bar{A}$-type eclipsed stacking (b) using a faulting scenario describing the transition from ordered $A\bar{A}$-type eclipsed stacking to ordered staggered stacking via randomly ordered mixed states, (c) using a faulting scenario describing the transition from ordered $A\bar{A}$-type eclipsed stacking to a random stacking order and (d) using a faulting scenario describing the transition from ordered $A\bar{B}$-type staggered stacking to a randomly oriented staggered stacking order.

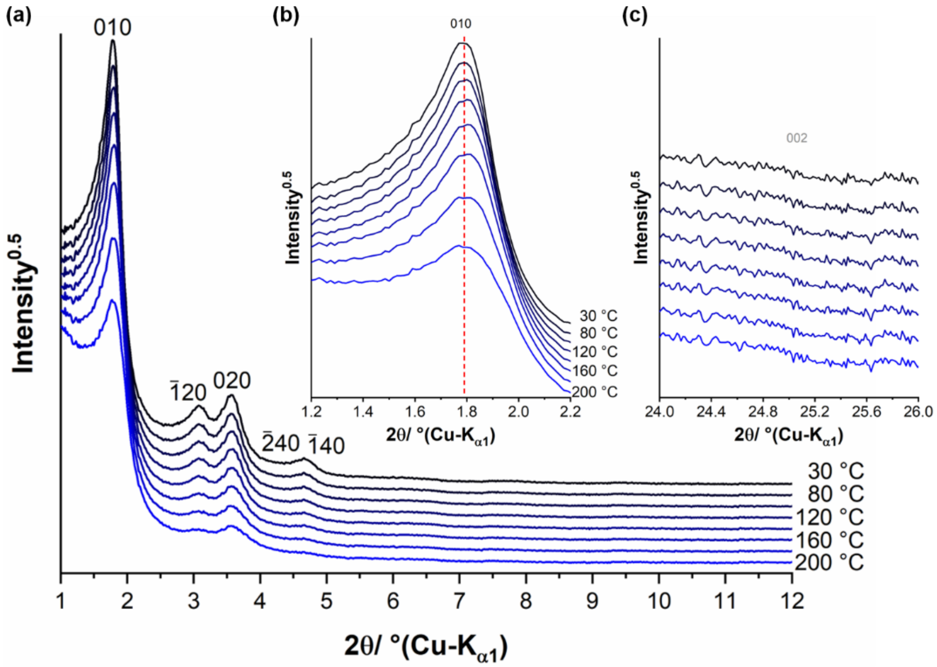

Figure S14. Temperature dependent *in situ* XRPD patterns of **PP-TAB** including selected reflection indices, (a) close-up of the low 2θ region, (b) close-up of the 010 reflections, (c) close-up of the 002 reflection.

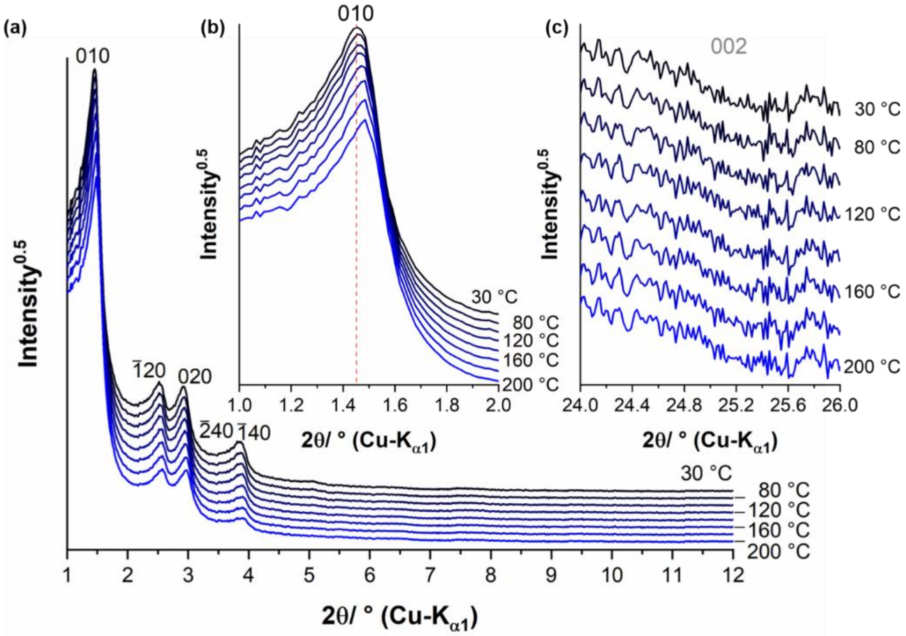

Figure S15. Temperature dependent *in situ* XRPD patterns of **PP-TATB** including selected reflection indices, (a) close-up of the low 2θ region, (b) close-up of the 010 reflections, (c) close-up of the 002 reflection.

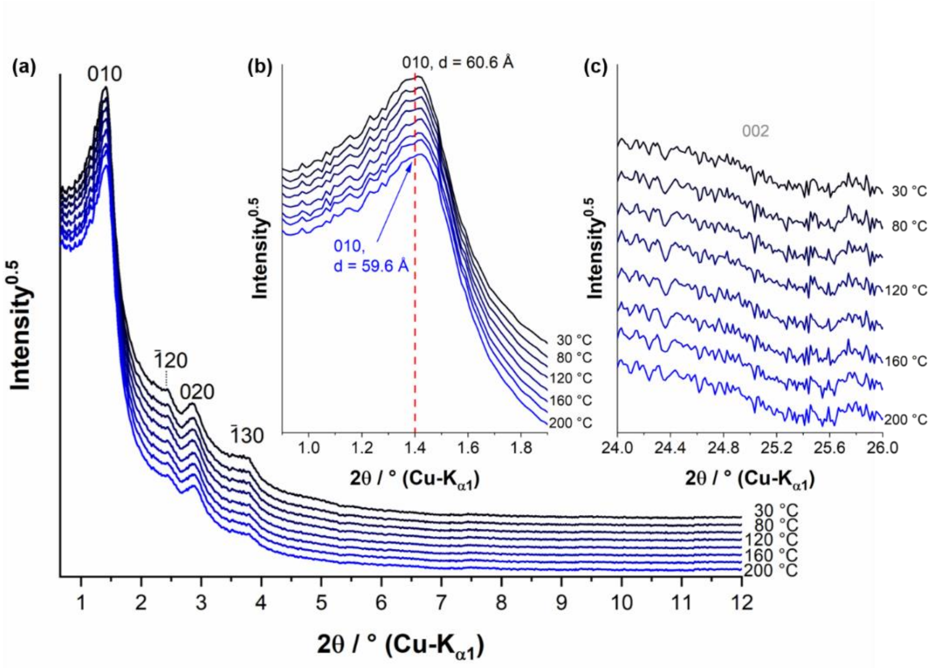

Figure S16. Temperature dependent *in situ* XRPD patterns of **mPP-TATB** including selected reflection indices, (a) close-up of the low 2θ region, (b) close-up of the 010 reflections, (c) close-up of the 002 reflection.

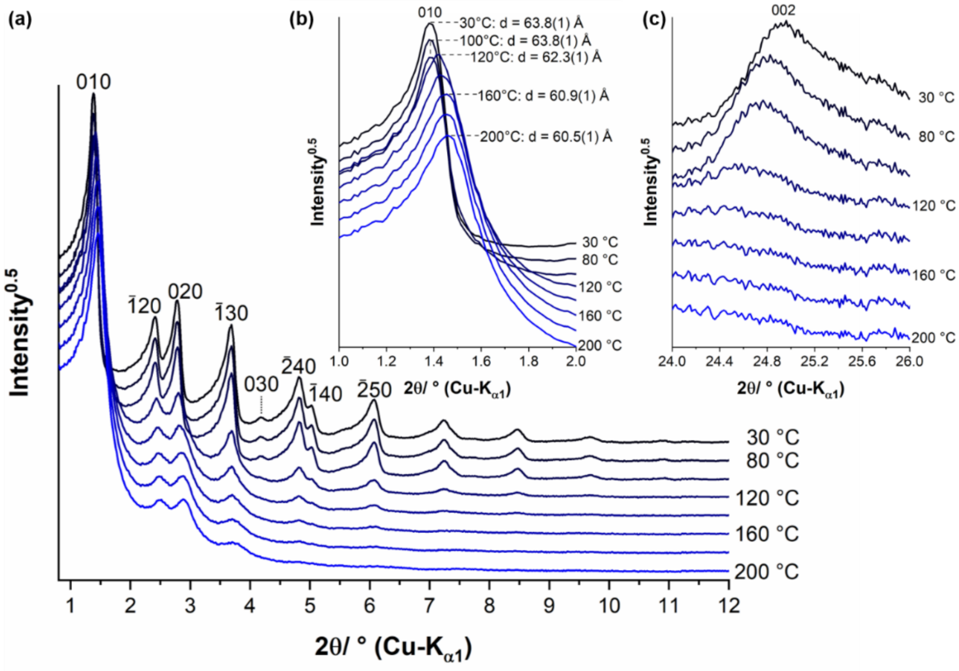

Figure S17. Temperature dependent *in situ* XRPD patterns of **dPP-TATB** including selected reflection indices, (a) close-up of the low 2θ region, (b) close-up of the 010 reflections, (c) close-up of the 002 reflection.

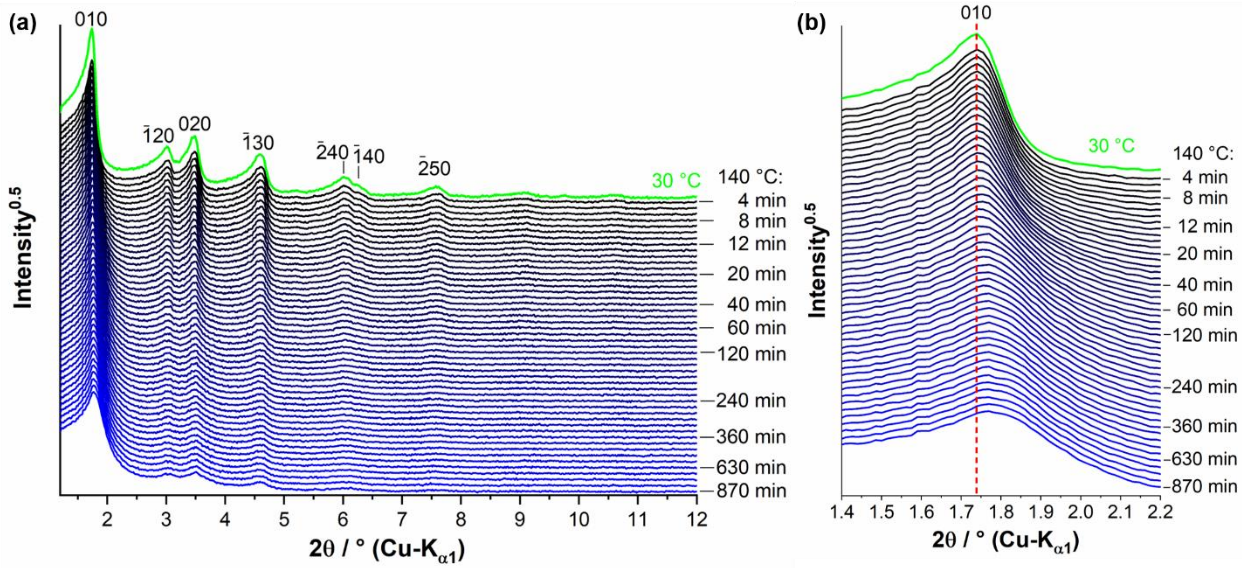

Figure S18. XRPD patterns of **mPP-TAB** collected at 30 °C (green pattern) and collected while holding the sample at 140 °C (black and blue patterns) for ca. 900 min (≡ 15 h), (a) excerpt of the low 2θ region, (b) excerpt showing the 010 reflection.

Table S2**.** Comparison of crystal structure features at room temperature and heating-induced structural changes of a series of investigated COFs.

**COF**	***a* and *b* / Å**		**thermal behavior**		
	**RT**	**200 °C**	**layer shifting**	**transition temperature**	**layer curving**
PP-TAB	56.62(9)	56.17(9)	slight	-	slight increase
mPP-TAB	58.46(4)	55.03(3)	strong	(120–140) °C	strong increase
dPP-TAB	58.49(7)	58.53(5)	no	-	no
PP-TAPB	69.29(6)	68.20(0)	slight	-	medium increase
mPP-TAPB	69.97(4)	68.81(2)	slight	-	medium increase
dPP-TAPB	73.17(6)	70.04(8)	strong	(100–120) °C	strong increase

S5 Gas Sorption Experiments

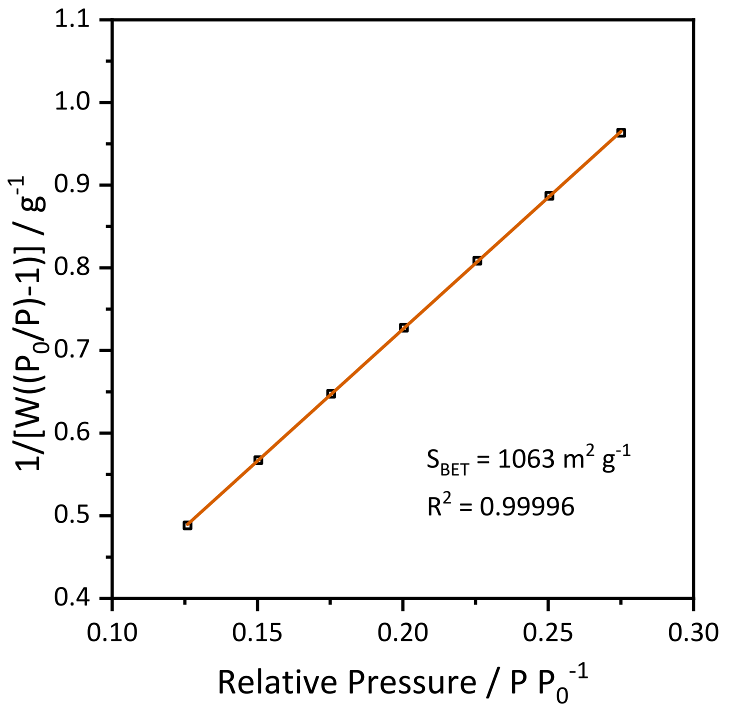

Figure S19. Multi-point BET surface area fit of **PP-TAB** derived from N_2 sorption isotherm.

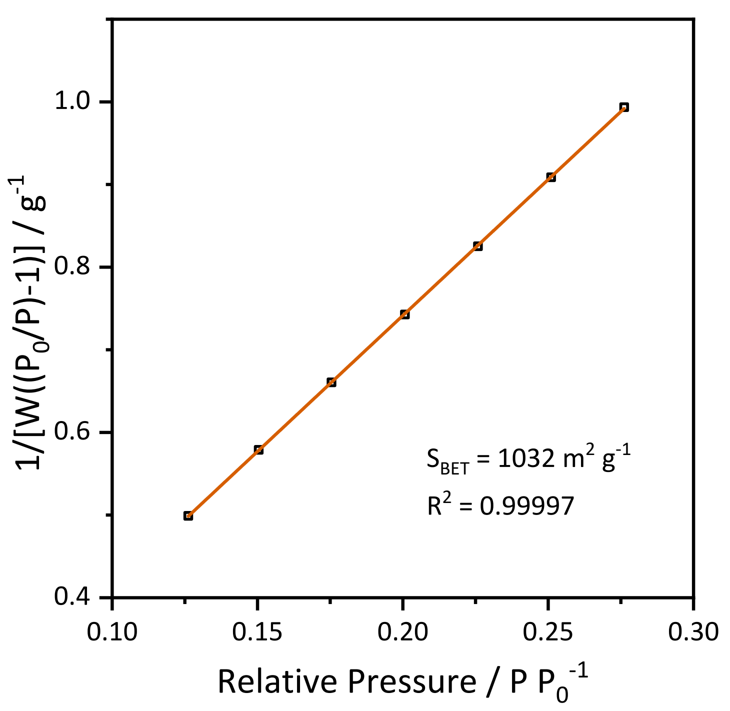

Figure S20. Multi-point BET surface area fit of **PP-TAPB** derived from N_2 sorption isotherm.

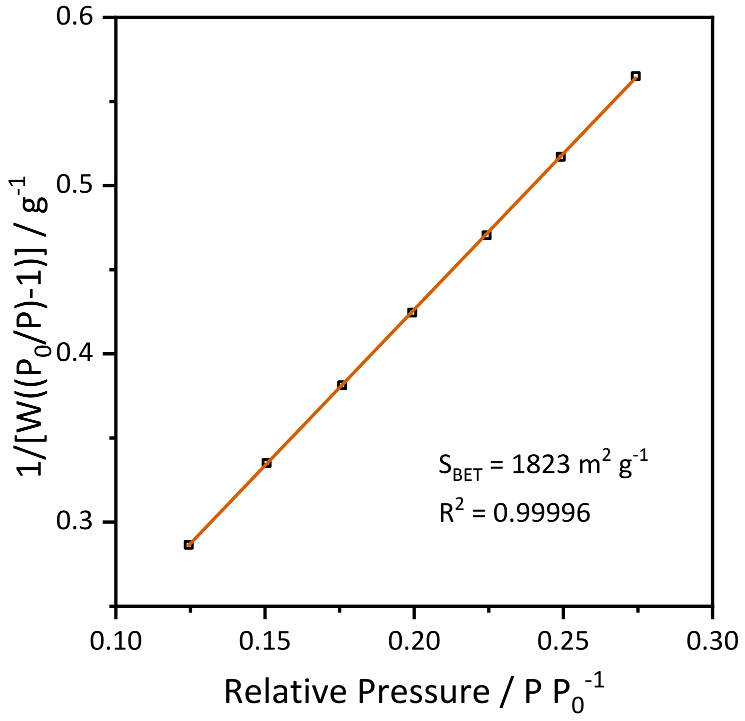

Figure S21. Multi-point BET surface area fit of **mPP-TAB** derived from N_2 sorption isotherm.

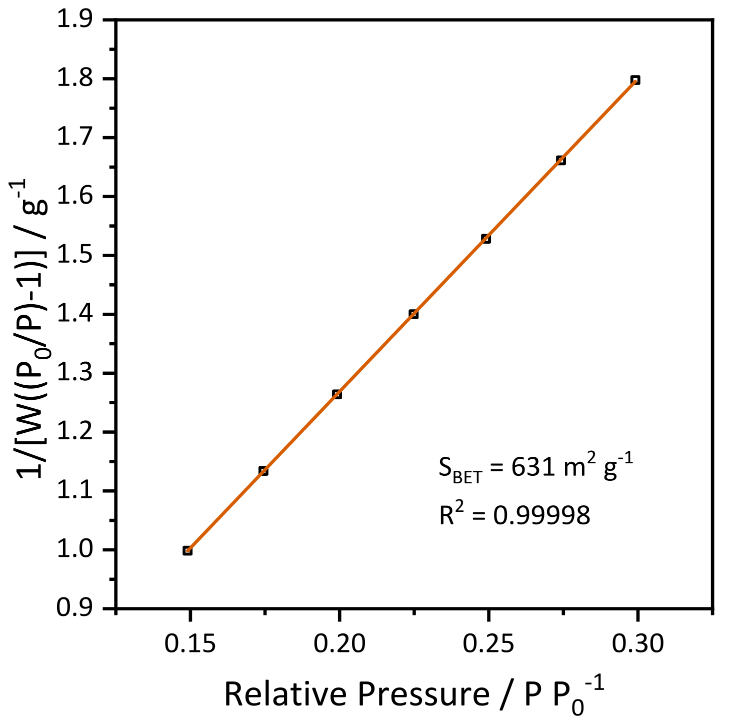

Figure S22. Multi-point BET surface area fit of **mPP-TAPB** derived from N_2 sorption isotherm.

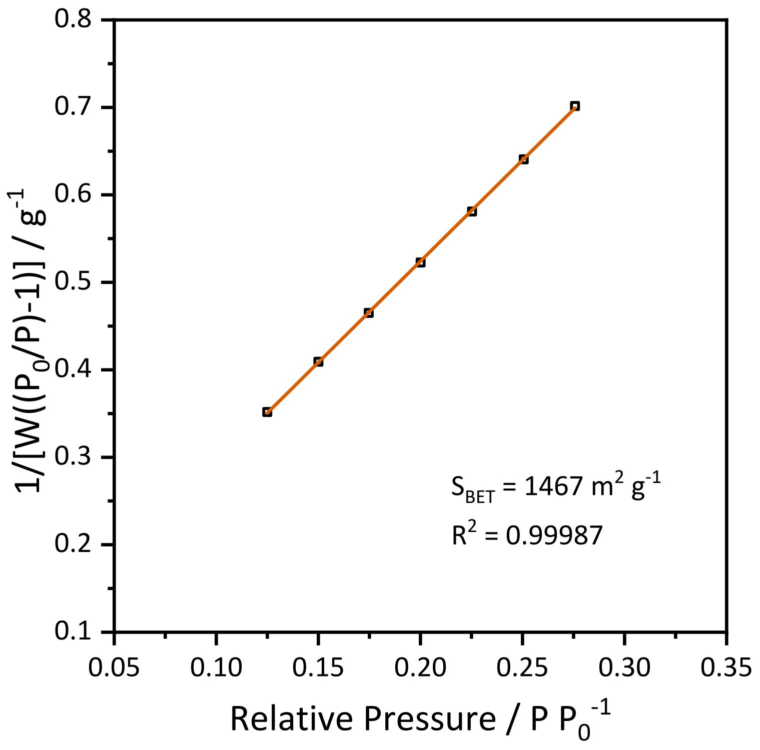

Figure S23. Multi-point BET surface area fit of **dPP-TAB** derived from N_2 sorption isotherm.

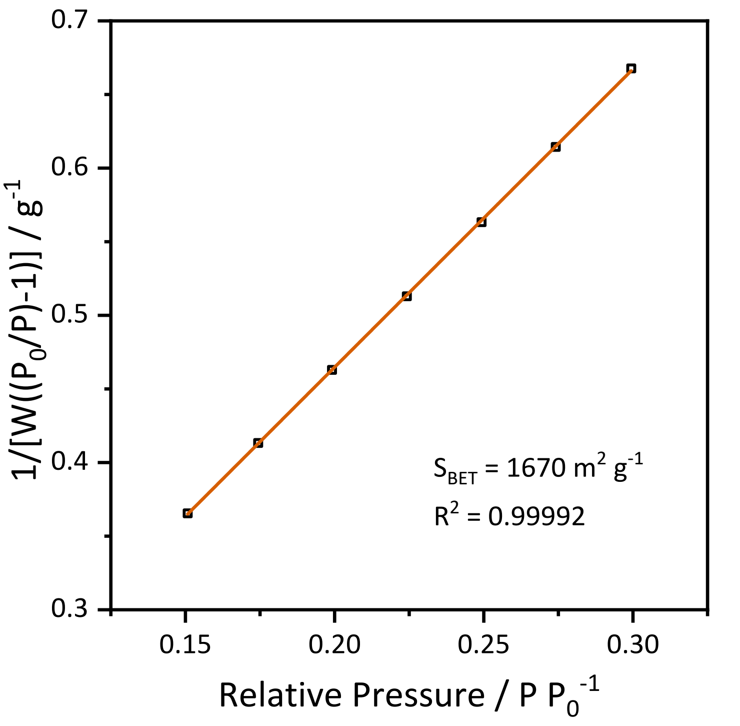

Figure S24. Multi-point BET surface area fit of **dPP-TAPB** derived from N_2 sorption isotherm.

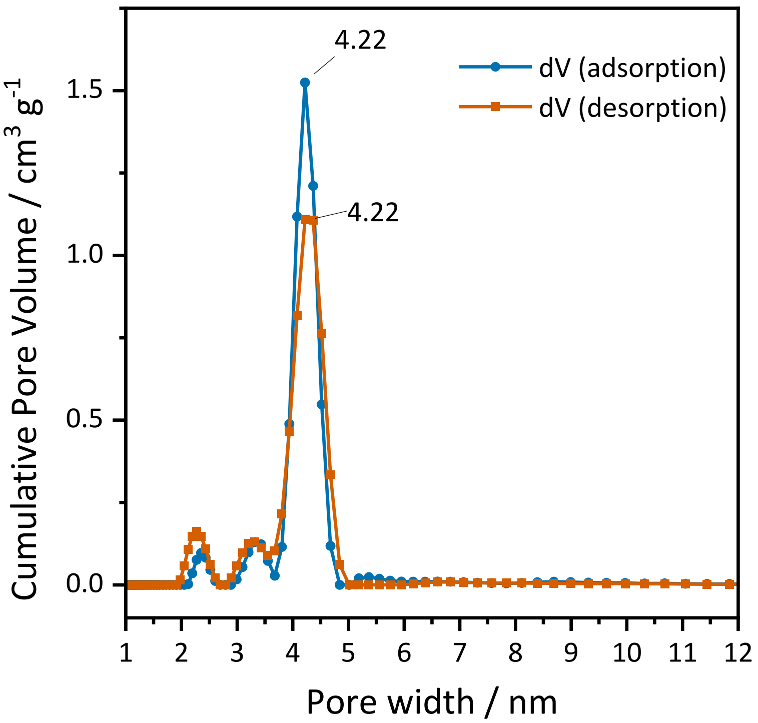

Figure S25. Comparison of PSD derived from the adsorption branch (blue) and the desorption branch (orange) for **PP-TAB**.

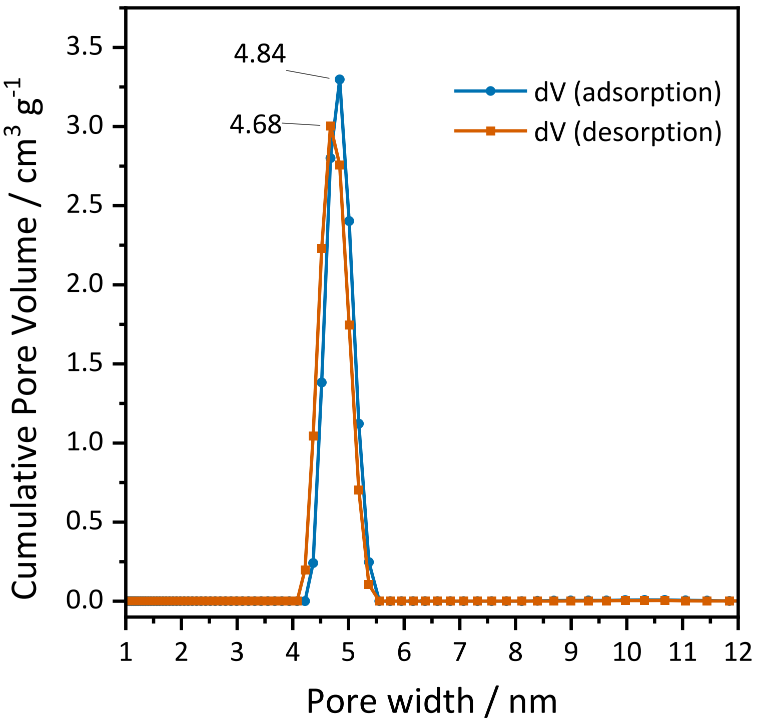

Figure S26. Comparison of PSD derived from the adsorption branch (blue) and the desorption branch (orange) for **PP-TAPB**.

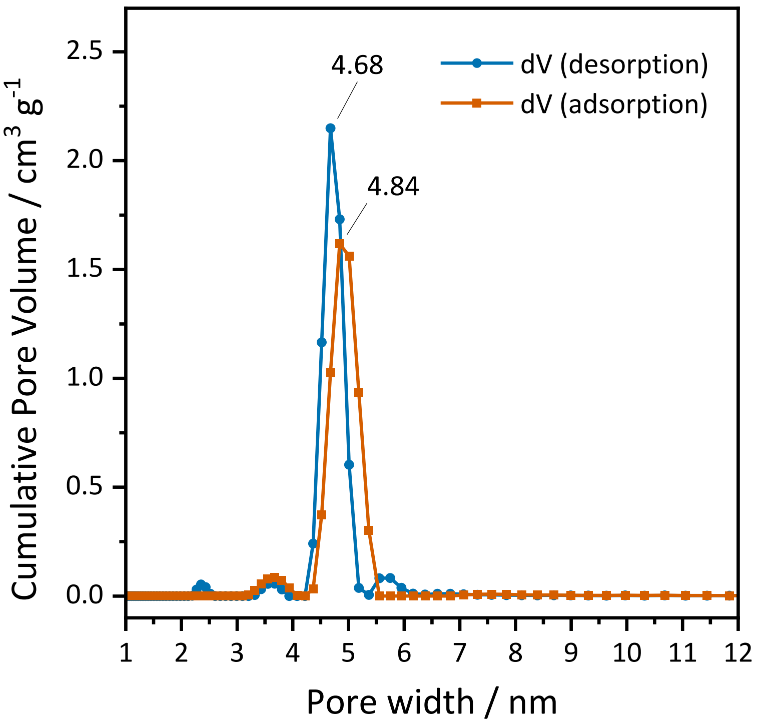

Figure S27. Comparison of PSD derived from the adsorption branch (blue) and the desorption branch (orange) for **mPP-TAB**.

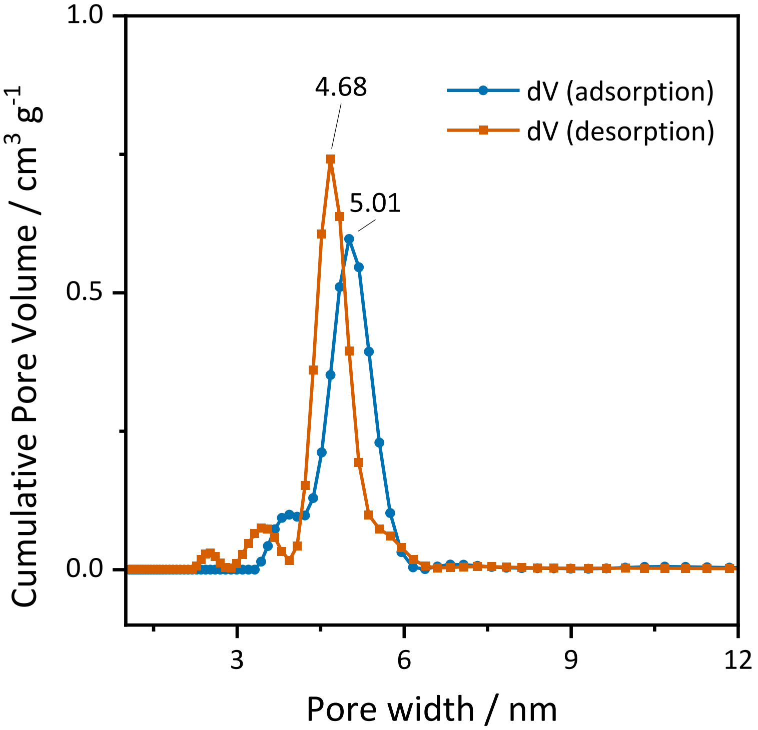

Figure S28. Comparison of PSD derived from the adsorption branch (blue) and the desorption branch (orange) for **mPP-TAPB**.

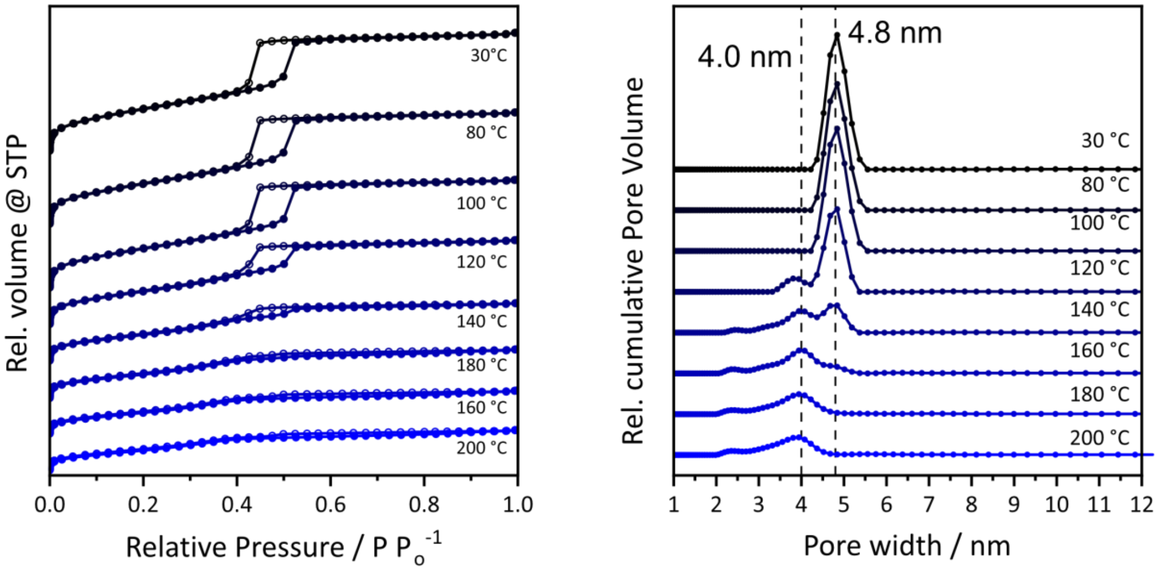

Figure S29. Evolution of isotherms (left) and pore size distribution (right) of mPP-TAB.

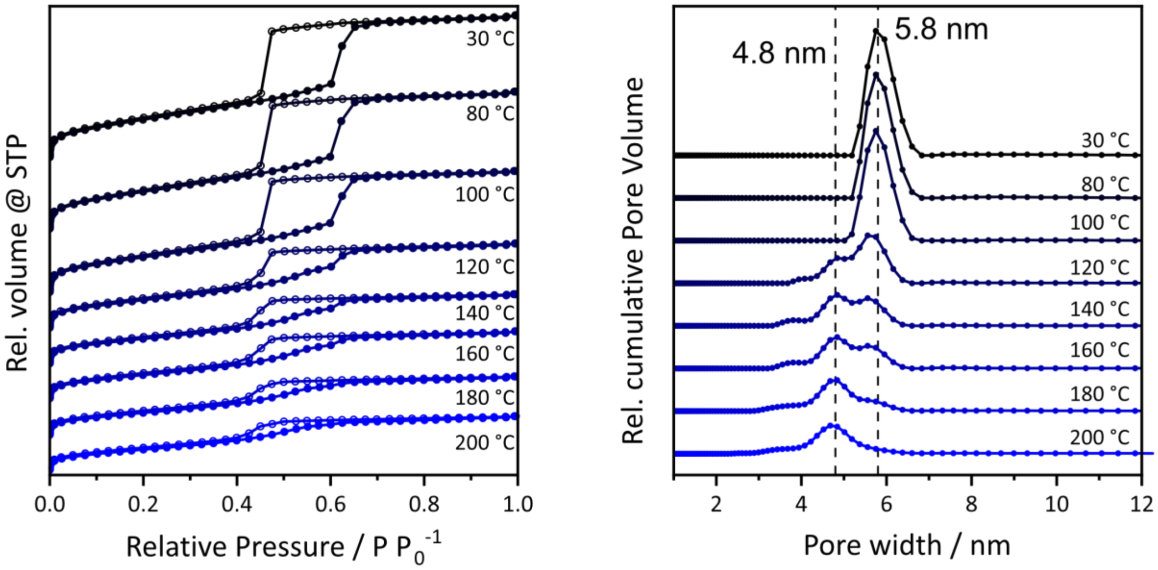

Figure S30. Evolution of isotherms (left) and pore size distribution (right) of dPP-TAPB.

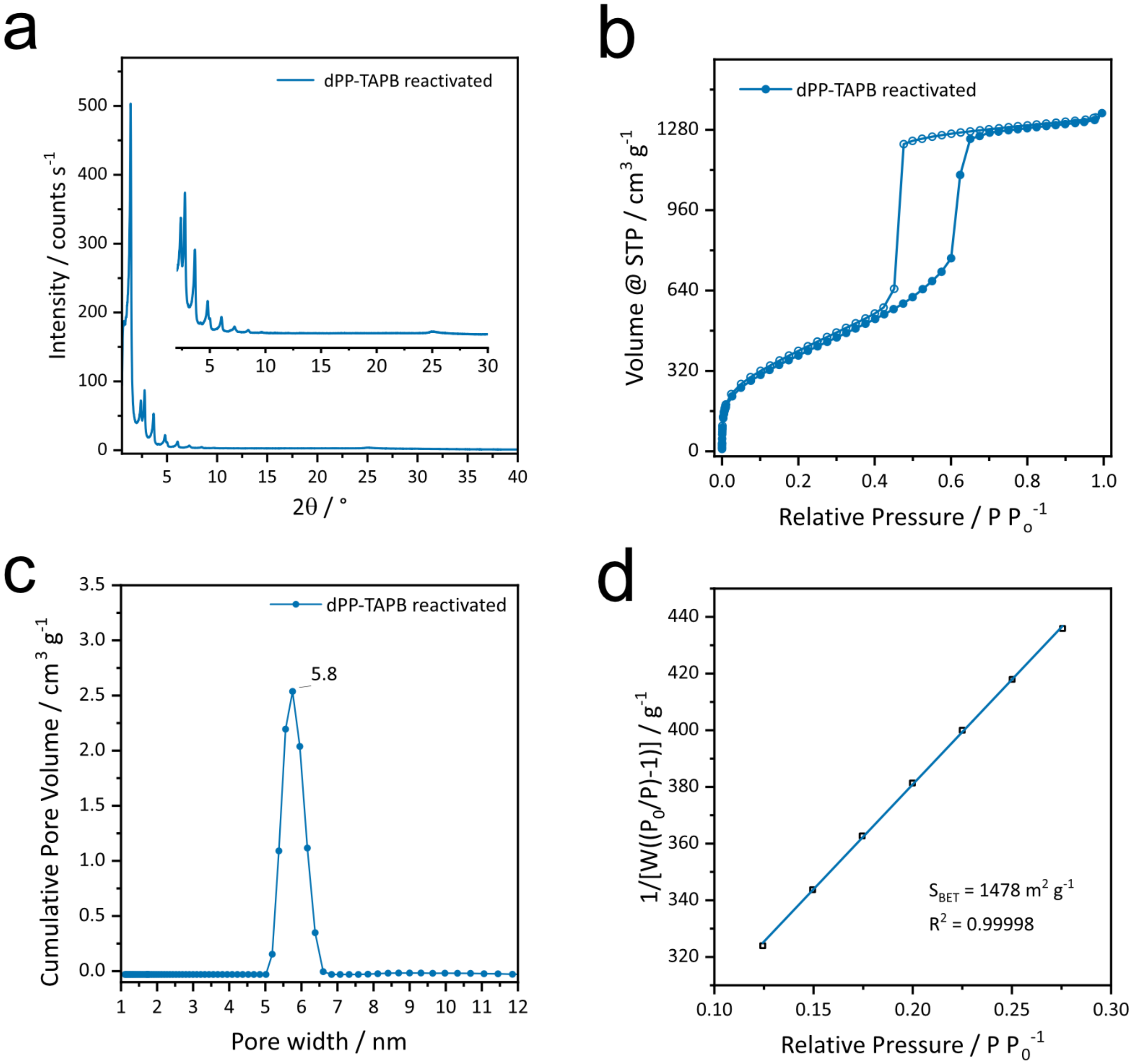

Figure S31. XRPD (Cu-K$_{\alpha 1}$) (**a**), N_2 Isotherms at 77 K (**b**), PSD derived from the adsorption branch (**c**), and multi-point BET surface area fit (**d**) of reactivated samples after heat treatment of **dPP-TAPB.**

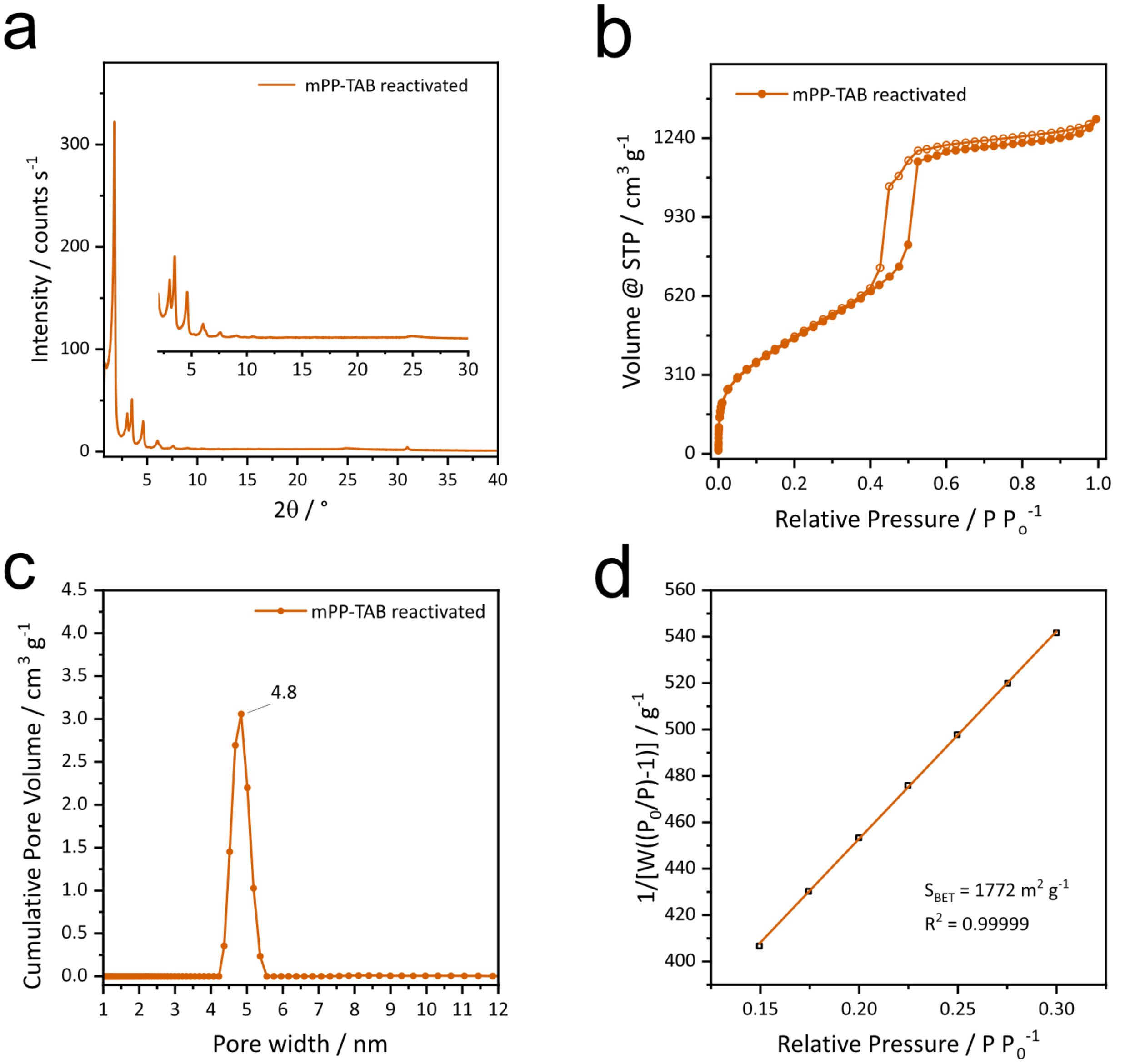

Figure S32. XRPD (Cu-K$_{\alpha1}$) (**a**), N_2 Isotherms at 77 K (**b**), PSD derived from the adsorption branch (**c**), and multi-point BET surface area fit (**d**) of reactivated samples after heat treatment of **mPP-TAB**.

S6 Solid State NMR

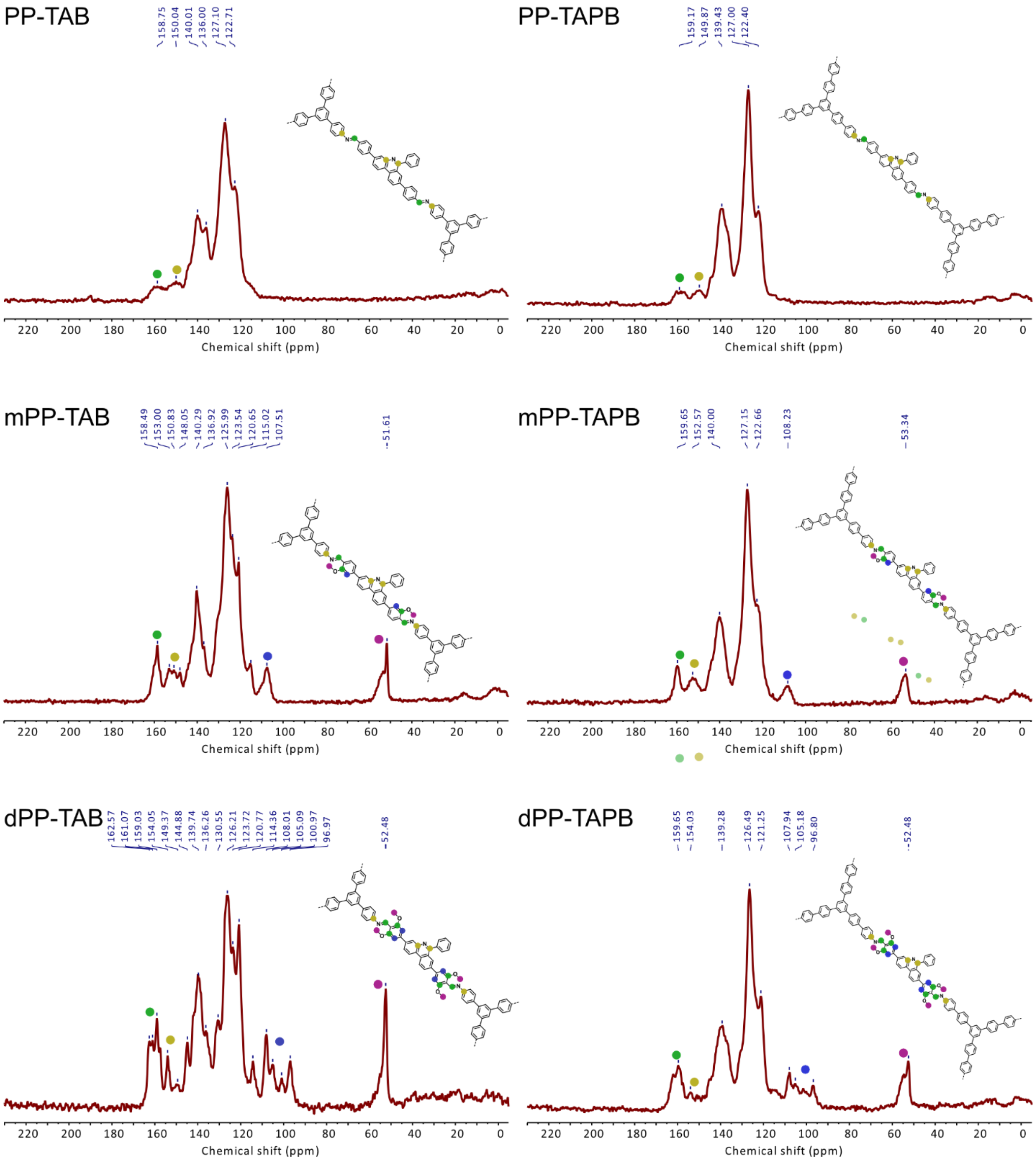

Figure S33. ^{13}C MAS ssNMR of **PP-TAB**, **PP-TAPB**, **mPP-TAB**, **mPP-TAPB**, **dPP-TAB** and **dPP-TAPB**.

S7 Thermogravimetric Analysis

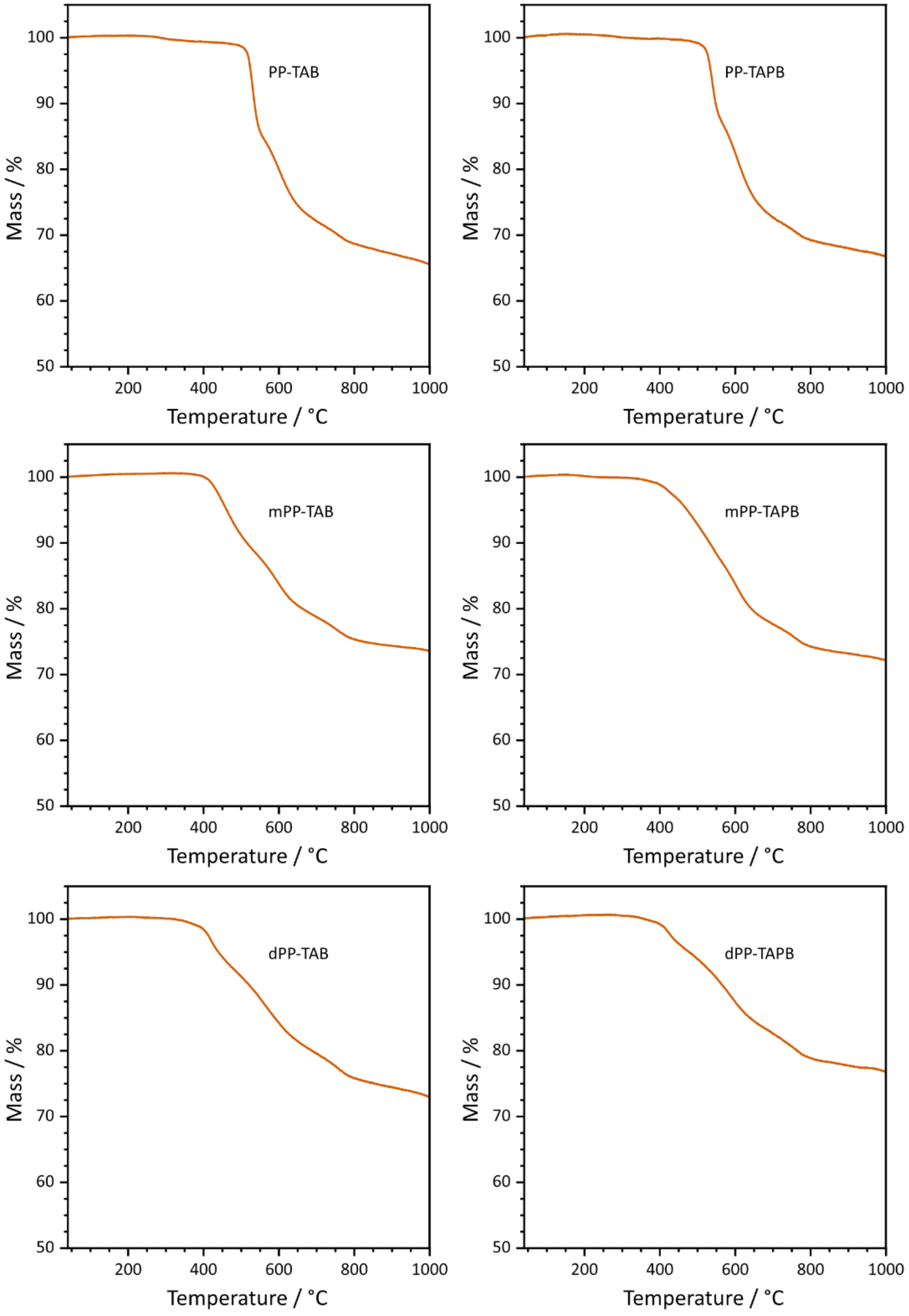

Figure S34. Thermogravietric analysis of **PP-TAB**, **PP-TAPB**, **mPP-TAB**, **mPP-TAPB**, **dPP-TAB** and **dPP-TAPB**.

S8 Liquid State NMR

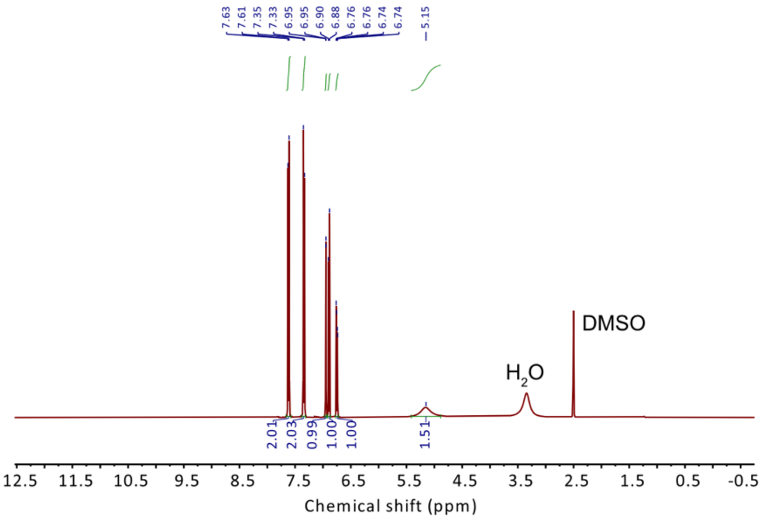

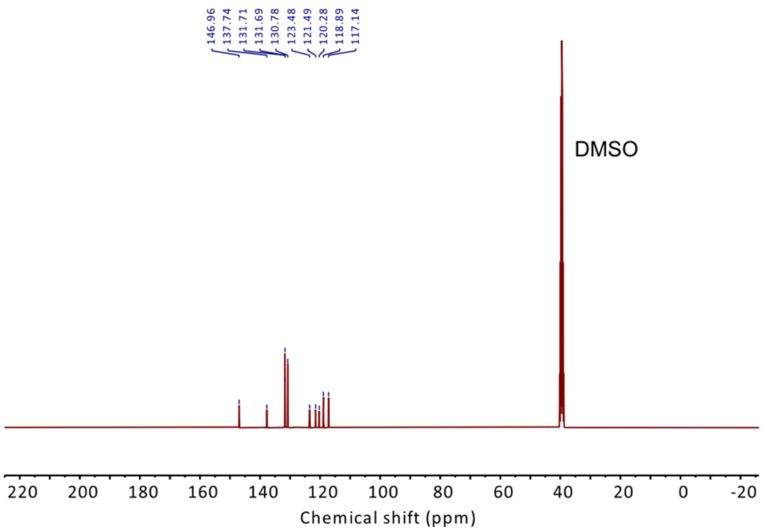

Figure S35. ^{1}H NMR (top) and ^{13}C NMR (bottom) of **4,4'-dibromo-[1,1'-biphenyl]-2-amine (2).**

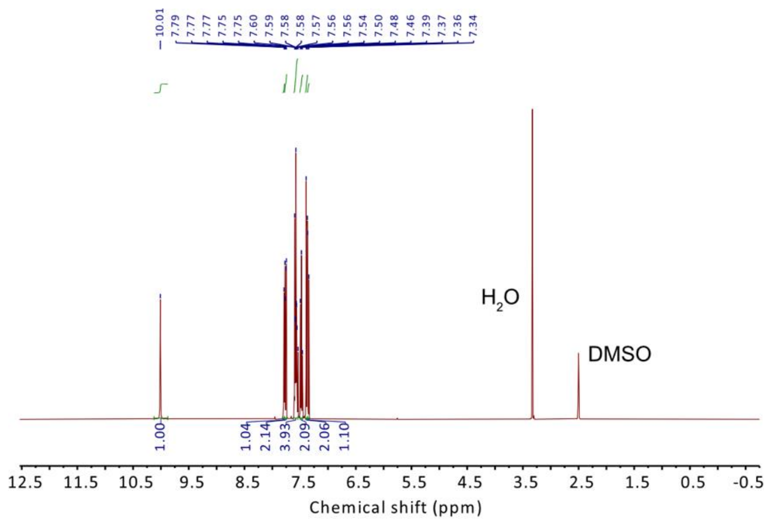

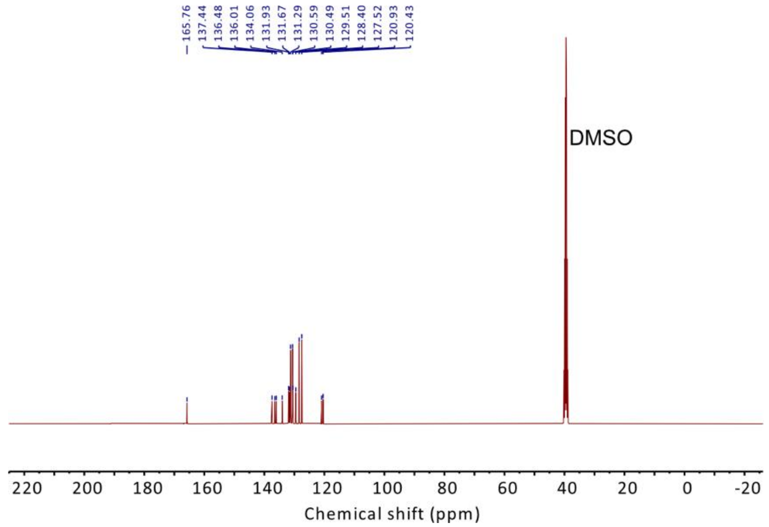

Figure S36. ^{1}H NMR (top) and ^{13}C NMR (bottom) of ***N*-(4,4'-dibromo-[1,1'-biphenyl]-2-yl)benzamide (3).**

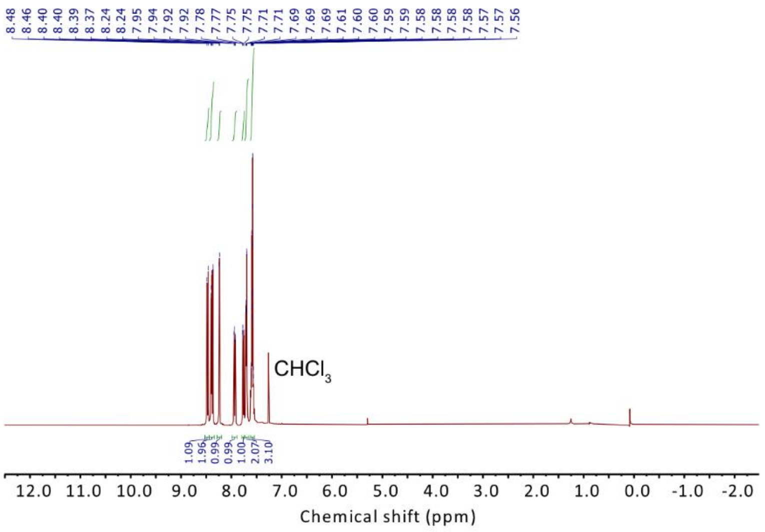

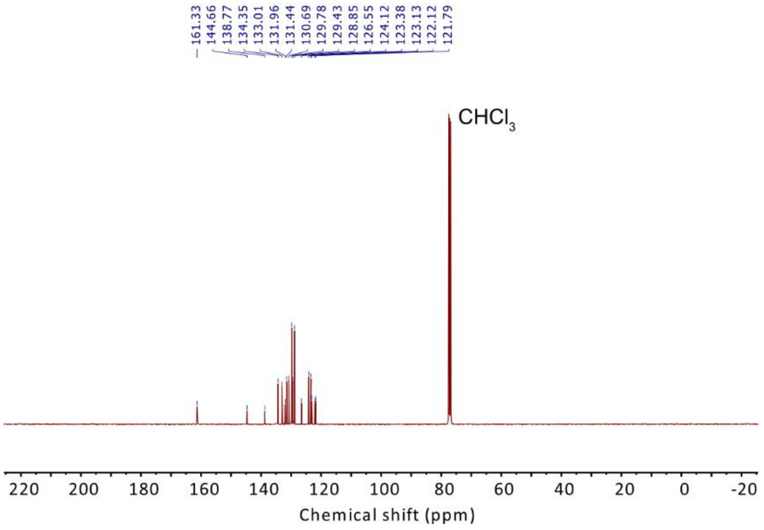

Figure S37. ^{1}H NMR (top) and ^{13}C NMR (bottom) of **3,8-dibromo-6-phenylphenanthridine (4).**

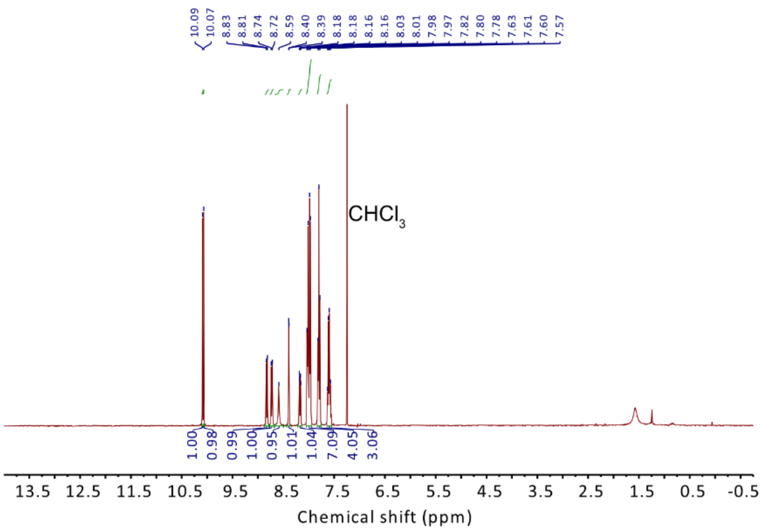

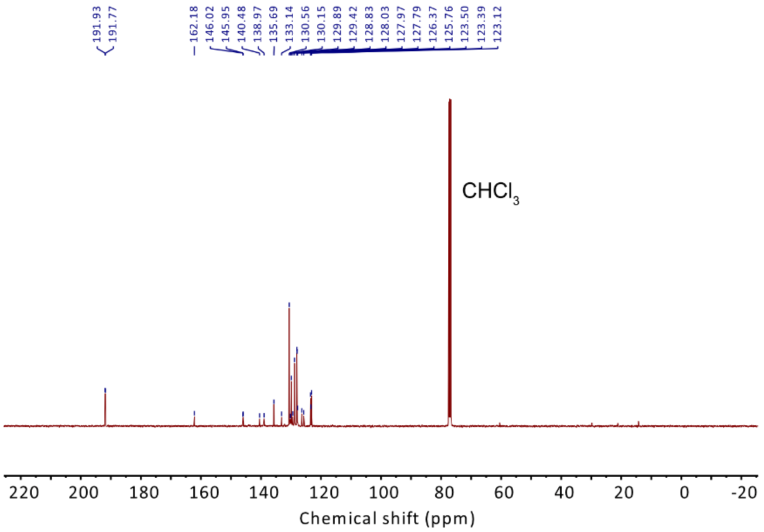

Figure S38. ^{1}H NMR (top) and ^{13}C NMR (bottom) of **4,4'-(6-phenylphenanthridine-3,8-diyl)dibenzaldehyde (PP).**

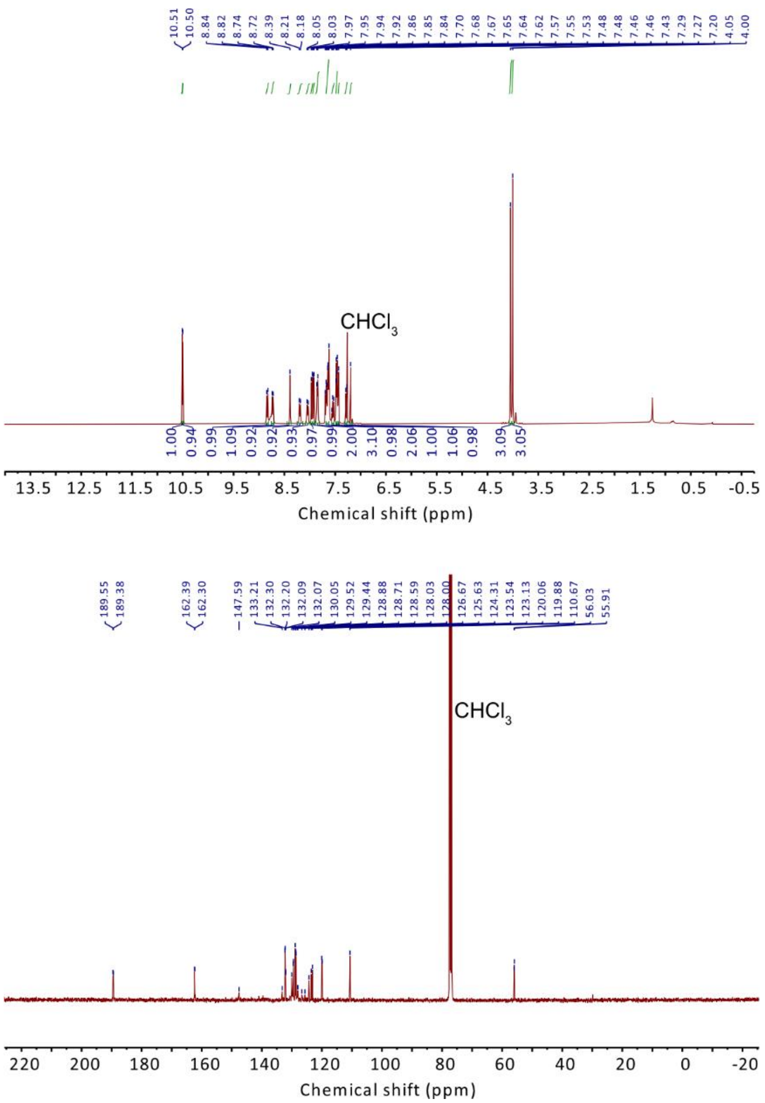

Figure S39. ^{1}H NMR (top) and ^{13}C NMR (bottom) of **4,4'-(6-phenylphenanthridine-3,8-diyl)bis(2-methoxybenzaldehyde) (mPP).**

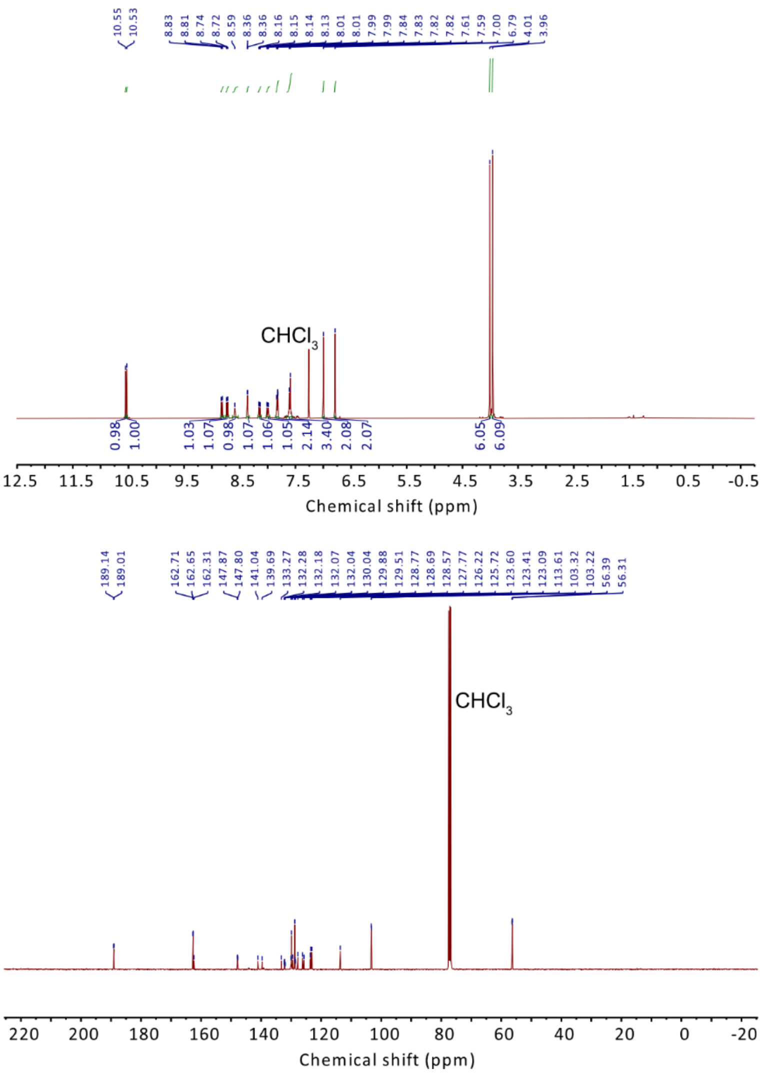

Figure S40. ^{1}H NMR (top) and ^{13}C NMR (bottom) of **4,4'-(6-phenylphenanthridine-3,8-diyl)bis(2,6-dimethoxybenzaldeyde) (dPP).**

S9. Geometry-Based Pore Size Calculation

For the experimentally obtained structures we performed a geometry-based pore size calculation. We constructed extended systems based on the obtained unit cells. This was done by in-plane displacement of layers, adjacent in stacking direction, of r_{disp} = 1.55 Å in a random direction. As the narrowest points are located in the plane of the investigated layers, we determined the maximum sphere radius from these planes for each layer as schematically shown in Figure S41, including up to 5 adjacent layers for taking care of the 3D nature of the resulting sphere. In order to obtain a geometric estimate of the pore sizes, we calculated the minimum distances of atoms r_{min} from the estimated pore center K_0. To account for the 3D nature of the problem, we then calculated these distances on a grid of points K_{shift} located around the estimated pore center. Afterwards we took the largest obtained value for the minimum distances η_{max} as representative for the maximum pore size corresponding to the investigated layer (L_2 in the picture). Additionally, we assumed the closest approachable distance as the van-der-Waals radius of the edge molecules. To determine the total pore size of our constructed arrangements, we averaged over the biggest spheres corresponding to each used layer L_i.

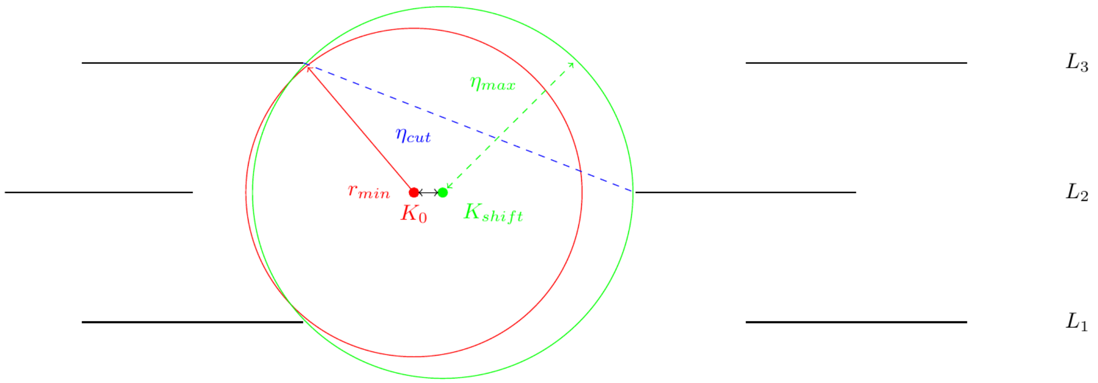

Scheme S4. Schematic drawing for geometry-based pore size calculation.

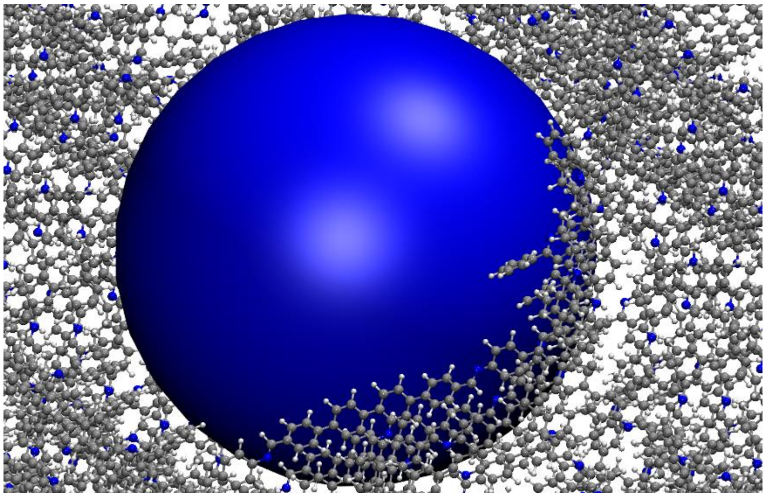

Figure S41. Sphere for layer 5 in a PP-TAB arrangement illustrating the utilized method.

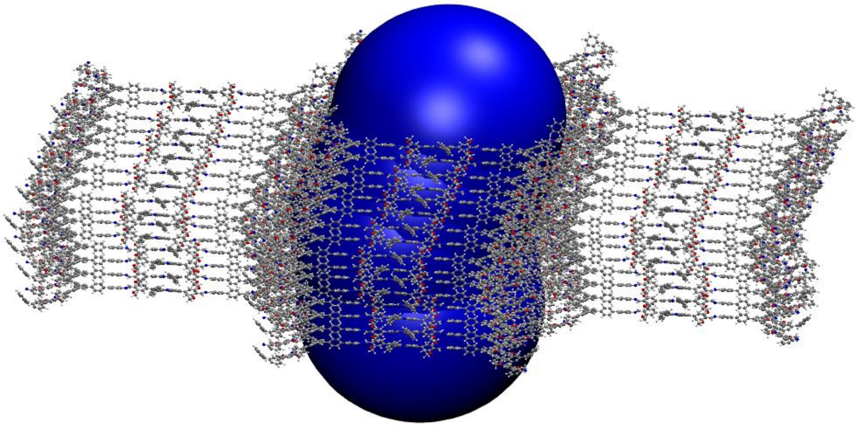

Figure S42. Distribution of spheres for 16 layers in a dPP-TAB arrangement with a small random offset between each layer to simulate the nearly eclipsed stacking.

Table S3: predicted values for geometry-based pore size calculation

	**Sphere Radius / Å**	**Pore Diameter / nm**
PP-TAB (staggered)	20.3A	4.05 nm
dPP-TAB (nearly eclipsed)	23.1A	4.62 nm
PP-TAPB (staggered)	22.8A	4.56 nm
dPP-TAPB (nearly eclipsed)	29.6A	5.92 nm

S10. Technical Details for the Theoretical Investigations

In the following we present the utilized setups for the computational investigations. Gas phase DFT geometry optimizations were performed using the TURBOMOLE program package for energy calculations and DL-find interfaced via CHEMSHELL for the geometry optimization.
Periodic DFT calculations were performed using the GPW approach as implemented in the CP2K program package. If not stated otherwise, total electronic energy calculations in the periodic case were performed using a PW cutoff of 300 Rydberg, GTH-PBE pseudo-potentials for the 2 1s core electrons, the PBE exchange and correlation functional as well as the TZV2P-GTH basis set.
All cell optimizations were performed using the conjugated gradient (CG) method as implemented in CP2K until the total forces acting on the molecules were below $4.5 \cdot 10^{-4}$ Hartree/Bohr with the relative change in geometry being below $3.0 \cdot 10^{-3}$ Bohr and a pressure tolerance of below 100 bar with respect to the environment was reached. If not stated otherwise, we used the default values for the convergence criteria.

Analysis of possible component orientations

For the component analysis of isolated molecular fragments building up the investigated COF system, geometry optimizations using TURBOMOLE were performed using the PBE functional with Grimme's D3 dispersion correction and Ahlrich's def2-TZVPP basis-set. We choose the PBE functional for comparison with periodic calculations described below using the Gaussian and plane waves approach as implemented in CP2K.

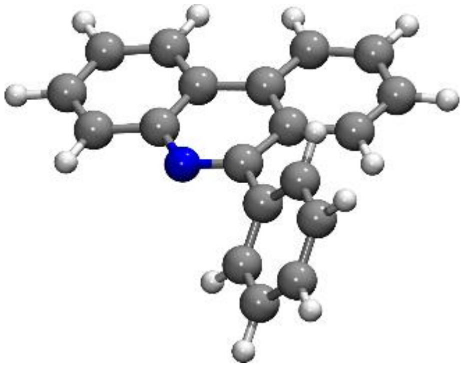

Figure S43. Phenylphenatridine component.

To determine the interaction energies, combinations of two fragments were optimized and compared to the total electronic energies of the optimized isolated molecules. Interaction energies for molecules a, b were calculated as:

$E_{\text{int}} = E_{\text{combination}} - E_{\text{a}} - E_{\text{b}}$

Initially, two molecules were placed close to each other, approximately 3.5 Å apart, for initializing the optimization procedure. For the orientations of two condensed triphenyl groups with respect to each other, the nomenclature follows the Scheme S4.

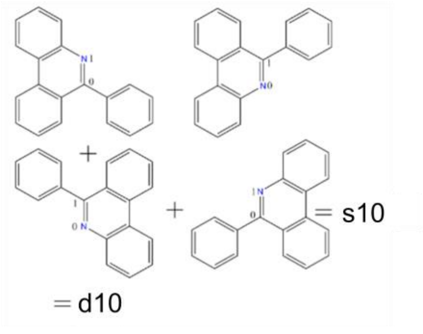

Scheme S5. Nomenclature scheme for phenylphenanthridine stacking options. The letters S and D indicate the side of the linker's attachment with respect to each other, whereas the numbers refer to the linker's attachment position at the middle ring. Additionally, we investigated the possibilities of T-shaped phenyl linker's arrangement as shown in Figure S40. T-shaped phenyls are indicated with the addition –T in the nomenclature.

Table S4. Energies of component's orientations as displayed is Scheme S4.

**Orientation**	***E*~int~ in (kcal/mol)**
s10-T	-13.304
s11-T	-13.590
d10	-12.198
d11	-12.482
s10	-14.826
s11	-14.079

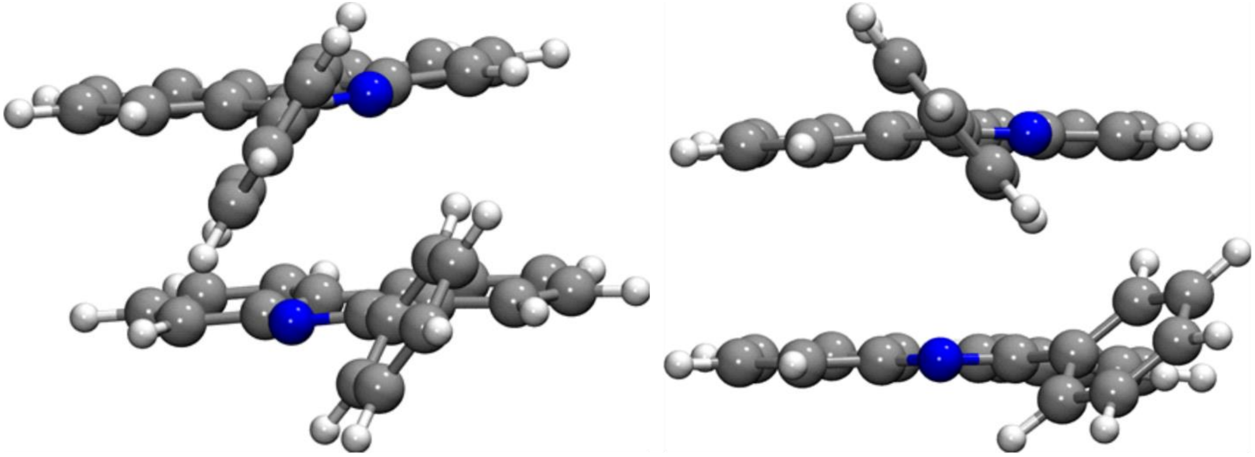

Figure S44. Exemplary structures for the S10 (left) and S10-T (right) orientations after the optimization.

Investigation of possible imine-linker orientations

The possible arrangements of the imine linkers with and without methoxy groups were investigated using the same procedure as for the phenyl linkers. The nomenclature was chosen to reflect the relative arrangement of the imine linker group, where the abbreviations alt and para refer to alternating and parallel oriented imines, respectively.

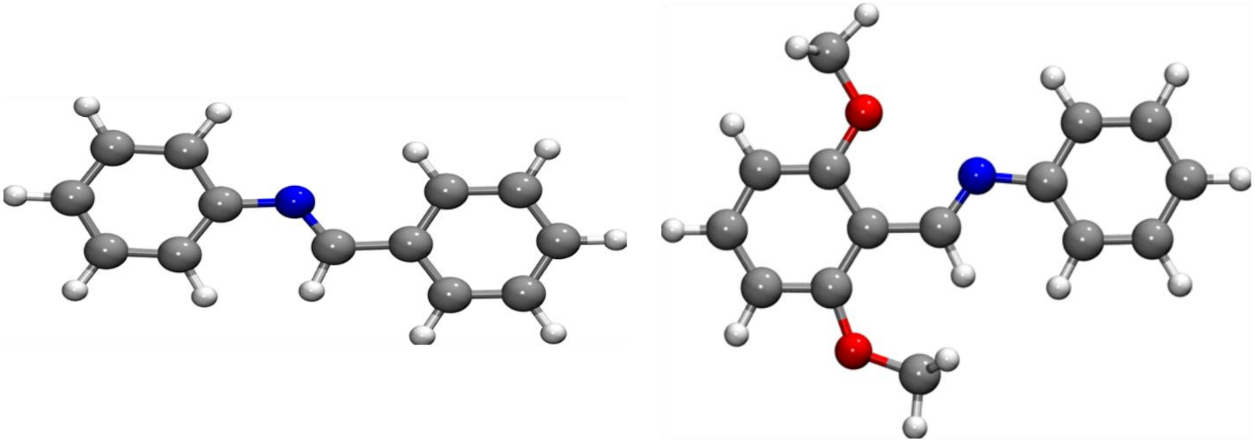

Figure S45. Components used to investigate possible imine linker orientations.

Table S5. Energies of component orientations as displayed is Figure S45.

Orientation	E_{int} in (kcal/mol)
Para	-7.68
Alt	-11.20
Methoxy-para	-9.24
Methoxy-alt	-11.94

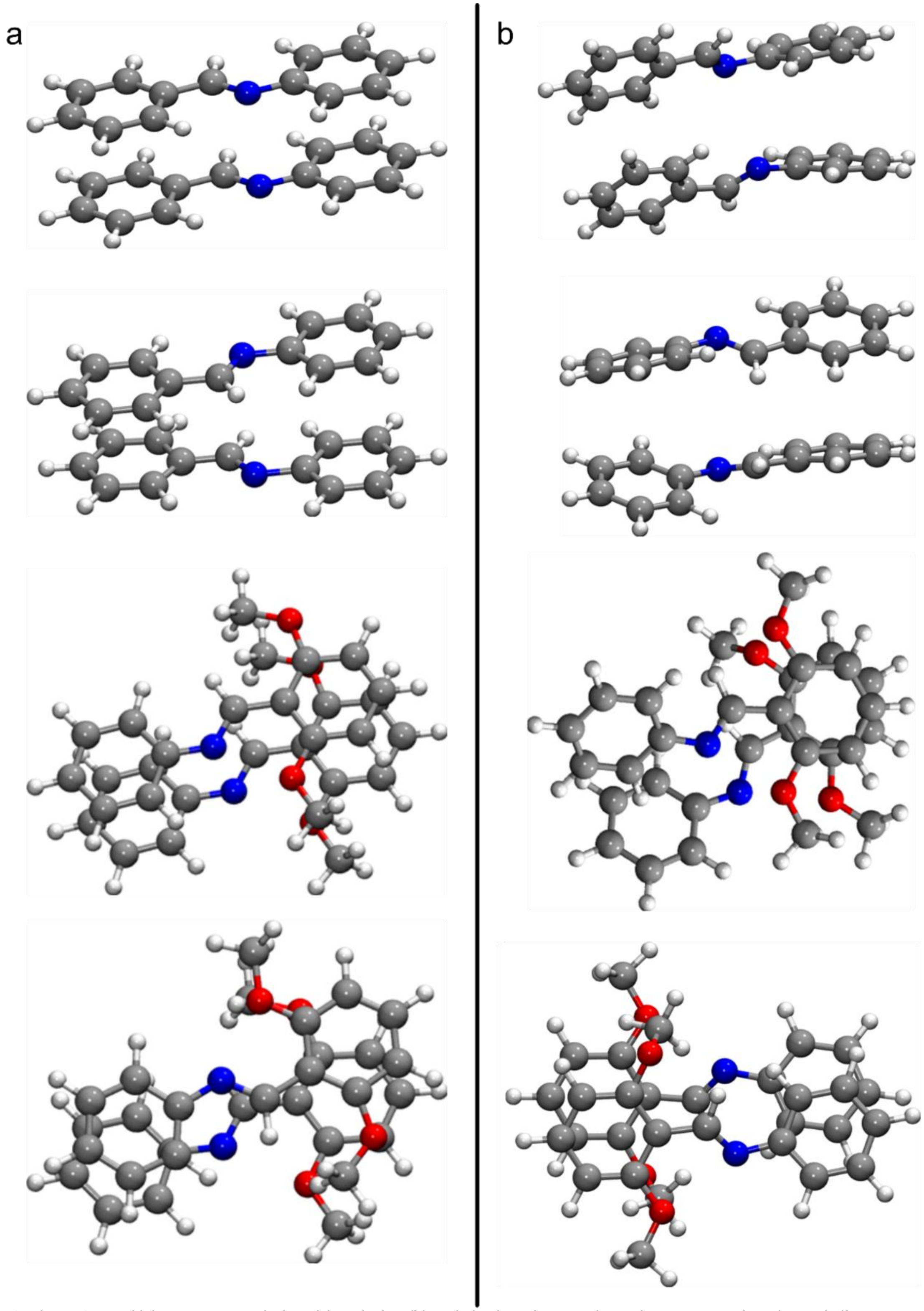

Figure S46. Figure S47. Initial arrangements before (**a**) and after (**b**) optimization of Para, Alt, Methoxy-para, and Methoxy-alt (from top to bottom), displaying the resulting arrangements after the optimization, showing that the molecules are rotated with respect to each other and, thus, not aligned as would be expected in the crystal structure.

From the obtained interaction energies, it can be seen that alternatingly oriented imine groups are energetically favorable. The methoxy versions of the linkers seem to be generally more stable and show smaller energy differences between alternatingly

and parallel-oriented imine groups. It was observed that the obtained optimized structures were rotated strongly from the initial plane, indicating that the underlying effects might not occur at the same level of magnitude in the crystalline structures as rotation is restricted there.

Calculation of electrostatic potential maps

For isolated components, electrostatic potential maps were calculated using the Gaussian08[5] program package. Geometries were optimized by means of DFT on the DGTZVP-PBE-D3 level of theory. For the optimized geometries reference densities and electrostatic potentials were visualized using Gauss-view the graphical interface to Gaussian. In the presented pictures, the electrostatic potential is mapped onto the isosurface of the density at an iso-value of 0.04 e/Bohr^3 with iso-values for the potential ranging from -0.02 to 0.4. The colors of the electrostatic potential maps indicate the energy of a positive probe charge with tones from blue to red indicating the transition from repulsion to attraction. Additional in-plane potential lines with normal vector (0,0,1) were included to show the influence of the electrostatic potential in close vicinity to the highlighted surface, yellow-colored regions correspond to repulsive red to attractive forces.

.

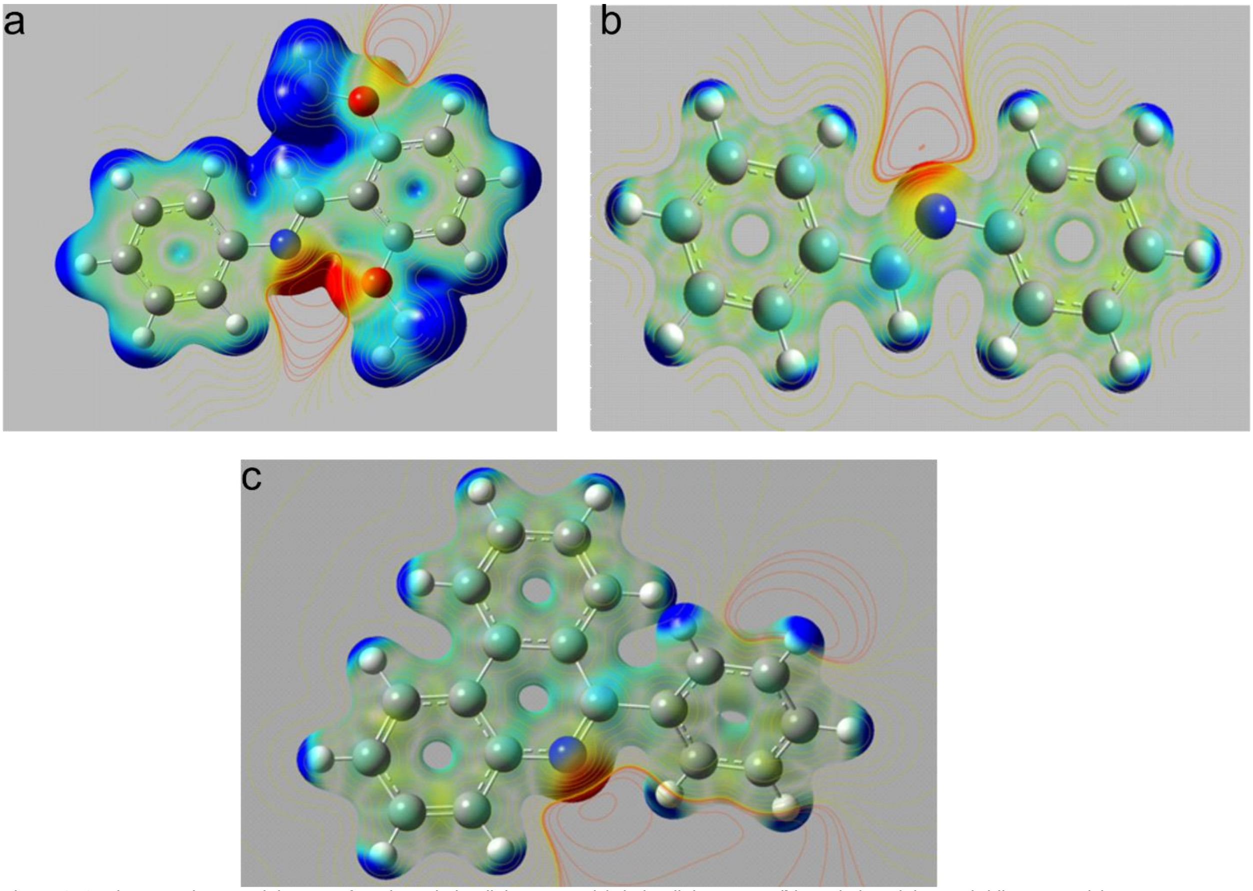

Figure S48. Electrostatic potential maps of methoxy-imine-linker-group (**a**), imine-linker-group (**b**), and phenylphenanthridine group (**c**).

Computation of periodic components (DFT/PBE-TZV2P-GTH)

In order to minimize in-plane rotational movement and allow for an estimation of stabilizing effects due to inter-layer interactions, we restrained the components by introducing PBCs and connecting the sides of the imine-linkers by additional phenyl groups to mimic the effects of the adjacent components in the full COF-system. We performed GPW-DFT cell optimizations on the TZV2P-GTH/PBE-D3 level of theory. The nomenclature in this section and the following is extended by referring to the components without methoxy groups as “simple”.

Energy differences are calculated as:

$$\Delta E = E_{alt} - E_{para}$$

Simple: ΔE = 12.74 kcal/mol
Methoxy: ΔE = -1.13 kcal/mol

The arrangement for alternatingly oriented imines is favorable for the simple blocks by a rather large amount 12.7 kcal/mol, however it becomes obvious that with additional methoxy groups, the difference between the two imine orientations is with ΔE = -1.13 kcal/mol rather small.

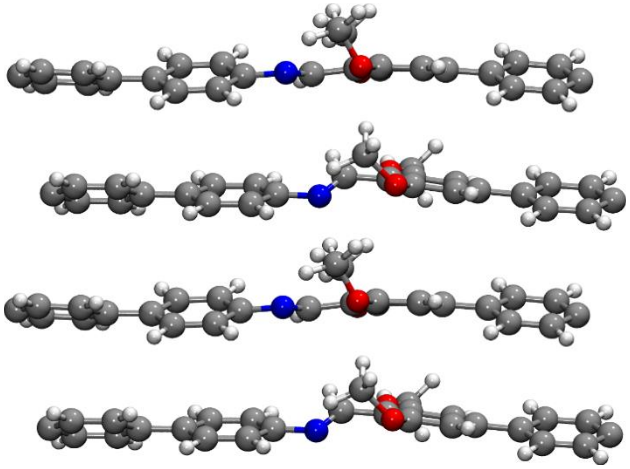

Figure S49. Exemplary periodic component of methoxy-imine-linker mimicking the effects of the adjacent components in the full COF-system.

Computation of periodic combinations of blocks (DFT/PBE-TZV2P-GTH)

In order to gain an overview on the influence of possible orientations of a whole wall of the COF pores we constructed combinations of blocks considering imine arrangements as well as phenyl linker orientations. Blocks were constructed for the simple-PP structure as well as the modified versions with one and two additional methoxy groups at the imine linkers. In order to consider the additional possibilities of methoxy group attachments to the imine sites we further distinguished in this case between parallel and anti-parallel arranged methoxy groups as can be seen in Fig.1-methoxy-cases. Calculations were performed using periodic boundary conditions with an orthorhombic unit cell. The initial cell vectors were chosen to be a=33, b=25.5, c=7.5 Angstroms, where b was chosen ad-hoc as a large value to minimize interactions between the arrangements in this direction (the dispersion cutoff was chosen to be 12 Angstrom). In order to guarantee the maximum flexibility for the components, the structure was connected via a C-C triple bond over the periodic extension. By doing so the rotation of components along the axis of alignment is possible while the in-plane rotation out of alignment is still suppressed, as can be expected for the full pore system. For every arrangement we performed cell-optimizations with simultaneous geometry optimization utilizing the BFGS method as implemented in CP2K. The total electronic energies were obtained by using the GPW scheme for the SCF calculations on the TZV2P-GTH/PBE-D3 level using the corresponding GTH-PBE pseudo-potentials. The obtained energies are always compared to the reference value that was lowest in energy and ,thus, are given in relative differences ΔE.

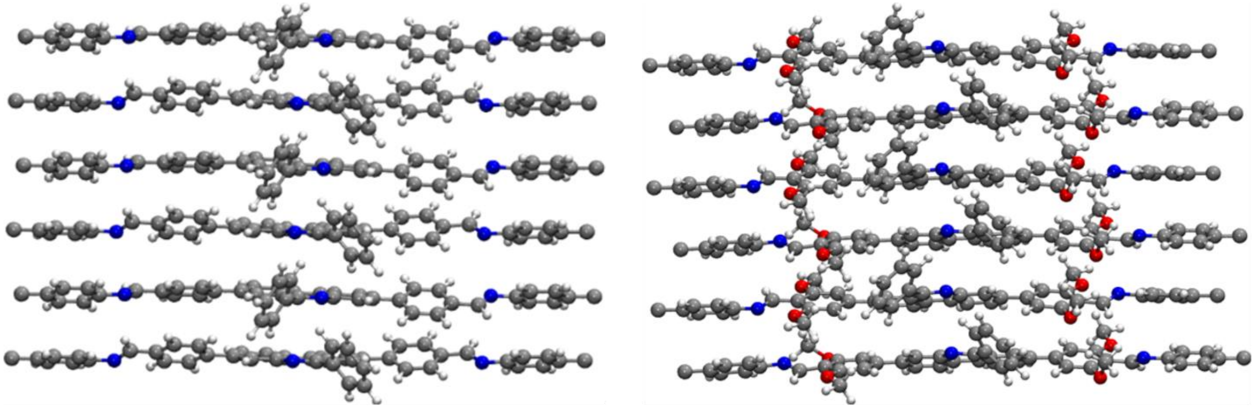

Figure S50. Exemplary segments of periodic combination of building blocks used for investigating imine arrangements as well as phenyl linker orientations for **PP-TAB** (left) and **dPP-TAB** (right).

Table S6. Results of cell-optimizations with simultaneous geometry optimization using DFT/PBE-TZV2P-GTH for **PP-TAB** mimicking periodic segments.

**Component-arrangement**	ΔE in kcal/mol
simple-alt-s10	0
simple-alt-s11	0.03
simple-mixed-s10	5.48
simple-mixed-s11	5.68
simple-para-s11	0.6
simple-para-s10	4.71
simple-alt-d10	1.94
simple-alt-d11	2.03
simple-mixed-d10	0.51
simple-mixed-d11	0.57
simple-para-d11	3.37
simple-para-d10	2.18

Table S7. Results of cell-optimizations with simultaneous geometry optimization using DFT/PBE-TZV2P-GTH for **dPP-TAB** mimicking periodic segments

**Component-arrangement**	ΔE in kcal/mol
methoxy-alt-s10	0
methoxy-alt-s11	21.71
methoxy-mixed-s10	1.04
methoxy-mixed-s11	1.83
methoxy-para-s11	4.42

methoxy-para-s10	0.5
methoxy-para-d11	18.04
methoxy-para-d10	17.16
methoxy-alt-d10	13.01
methoxy-alt-d11	34.83
methoxy-mixed-d10	15.3
methoxy-mixed-d11	17.55

For the additional analysis for component arrangements with one methoxy group additional distinguishing para and anti methoxy group arrangements. The nomenclature is thus further extended by an abbreviation -para and -anti after the identifier for the phenyl linker arrangement. In Figure S48, the additional differentiation is shown at the example of the para-S10 orientation.

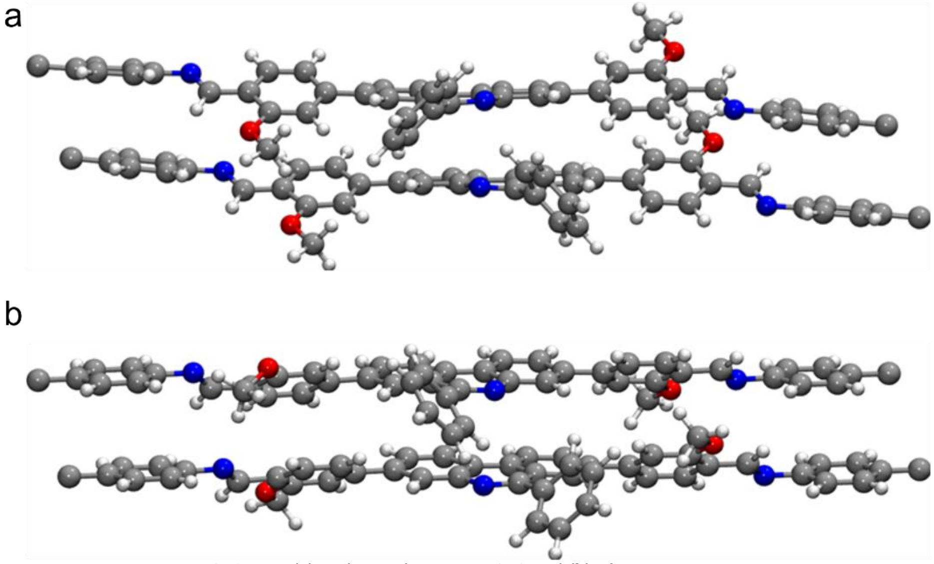

Figure S51. Component arrangements para-S10-para (**a**) and 1-methoxy-para-S10-anti (**b**) of **mPP-TAB**.

Table S8. Results of cell-optimizations with simultaneous geometry optimization using DFT/PBE-TZV2P-GTH for **dPP-TAB** mimicking periodic segments.

**Component-arrangement**	ΔE in kcal/mol
1-methoxy-para-s10-para	0
1-methoxy-para-d11-anti	12.98
1-methoxy-alt-d11-para	13.51
1-methoxy-alt-d11-anti	25.6
1-methoxy-alt-s10-anti	23.24
1-methoxy-alt-s10-para	10.26
1-methoxy-alt-s11-anti	24.58

1-methoxy-alt-s11-para	8.78
1-methoxy-para-s11-anti	14.47
1-methoxy-para-s11-para	3.17
1-methoxy-para-s10-anti	19.09
1-methoxy-para-d11-para	13.17
1-methoxy-alt-d10-anti	24.00
1-methoxy-alt-d10-para	11.80
1-methoxy-para-d10-anti	10.02
1-methoxy-para-d10-para	14.12

The results for the differentiation between para- and anti-arrangement for the methoxy groups show, that the para arrangement is overall more stable by a significant amount leading to the conclusion that this arrangement is the more probable of the two, regardless of the other components orientation. Furthermore it suggests that interactions between two methoxy groups are stabilizing.

Comparison of relative energies of isolated components (GFN-xTB)

Relative stability of components were analyzed by means of GFN-xTB after comparing the total electronic energies obtained by in vacuo geometry optimizations. The enumeration follows the scheme presented in Scheme S4. The relative interaction energies were compared to the isolated molecules and are given in Kcal/mol in relation to the isolated molecules.

Table S9. Relative energies of isolated phenylphenanthridine components.

**Component-arrangement**	ΔE in kcal/mol
d10-displaced	-14.684
d11-displaced	-13.425
s10-90grad	-13.667
s11-90grad	-14.156
d10	-12.093
d11	-12.905
s10	-17.600
s11	-15.636

Investigation of periodic combinations of blocks (GFN-xTB)

In order to investigate the influence of the adjacent components on the possible stacking behavior, we performed cell optimization with simultaneous geometry optimization using GFN-xTB as implemented in the CP2K program package. As we wanted to minimize the possibility of the components to shift and rotate out of plane as it was observed to happen in the gas

phase calculations, we utilized periodic boundary conditions. The initial cell was chosen to be an orthorhombic lattice with cell vectors of length a=33, b=25.5, c=7.5 Angstrom, where b was chosen ad-hoc as a large value to minimize interactions between the arrangements in this direction (the dispersion cutoff was chosen to be 12 Angstrom). The enumeration used for the orientations of the phenyl linkers is the same as in section S12. To judge the influence of methoxy groups upon the organization, components were constructed with one methoxy group and 2 groups per imine-linker alongside the bare structures. The energies were then calculated and the different arrangements of units were compared concerning their total energies, where relative energies to the structure lowest in energy are given in Table S9. It has to be noted here, that the unit cells belonging to each structure after optimization can be found along the obtained geometries in the attached PDB-files, but are left out as we were mainly interested in the stability of the overall components.

Table S10. Results of cell-optimizations with simultaneous geometry optimization using GFN-xTB for **PP-TAB** mimicking periodic segments.

Component-arrangement	Relative Energy shift (kcal/mol)
simple-para-s10	1.14
simple-alt-s10	3.26
simple-alt-s11	6.49
simple-mixed-s10	5.09
simple-mixed-s11	8.21
simple-para-s11	1.14
simple-alt-d10	7.89
simple-alt-d11	0.91
simple-mixed-d10	0.55
simple-mixed-d11	2.83
simple-para-d11	2.42
simple-para-d10	0.00

Table S11. Results of cell-optimizations with simultaneous geometry optimization using GFN-xTB for **mPP-TAB** mimicking periodic segments.

Component-arrangement	Relative Energy shift (kcal/mol)
1-methoxy-para-s10-para	0.00
1-methoxy-alt-d10-anti	17.07
1-methoxy-alt-d10-para	10.26
1-methoxy-para-d10-anti	14.11
1-methoxy-para-d10-para	9.78
1-methoxy-para-d11-para	1.66
1-methoxy-para-d11-anti	8.57
1-methoxy-alt-d11-para	7.89

1-methoxy-alt-d11-anti	18.30
1-methoxy-alt-s10-anti	11.06
1-methoxy-alt-s10-para	7.82
1-methoxy-alt-s11-anti	14.15
1-methoxy-alt-s11-para	10.44
1-methoxy-para-s11-anti	9.61
1-methoxy-para-s11-para	2.87
1-methoxy-para-s10-anti	7.19

Table S12. Results of cell-optimizations with simultaneous geometry optimization using GFN-xTB for **dPP-TAB** mimicking periodic segments.

**Component-arrangement**	**Relative Energy shift (kcal/mol)**
methoxy-para-s11	0.00
methoxy-alt-s10	1.85
methoxy-alt-s11	9.77
methoxy-mixed-s10	1.03
methoxy-mixed-s11	6.91
methoxy-para-s10	3.80
methoxy-alt-d10	6.51
methoxy-alt-d11	69.46
methoxy-mixed-d10	10.49
methoxy-mixed-d11	6.07
methoxy-para-d11	1.94
methoxy-para-d10	8.05

Calculation of a reference pathway from $A\overline{A} \rightarrow A\overline{B}$ for comparing (GPW-DFT) and (GFN-xTB)

In order to justify the use of GFN-xTB as a tool to analyze the potential energy surface of COFs under displacements a pathway from AA’ stacking to AB stacking was calculated with equidistant shifting vectors along the direct line of displacement with cell optimization and simultaneous geometry optimization performed at every of the 20 reference structures. The resulting path consisting of total energy values was then re-investigated using GPW-DFT single-point calculations using CP2K on the TZV2P-GTH/PBE-D3-level for comparison. The relative energies in reference to the initial structure, shown in Figure S52, are showing that DFT and GFN-xTB energy calculations show qualitatively similar tendencies for relative energy shifts, thus, we used GFN-xTB for the further analysis as it is by far the computationally cheaper method. The displacement from $A\overline{A}$ to $A\overline{B}$ stacking was split into 20 steps, with the fraction of the displacement vector acting as x coordinate.

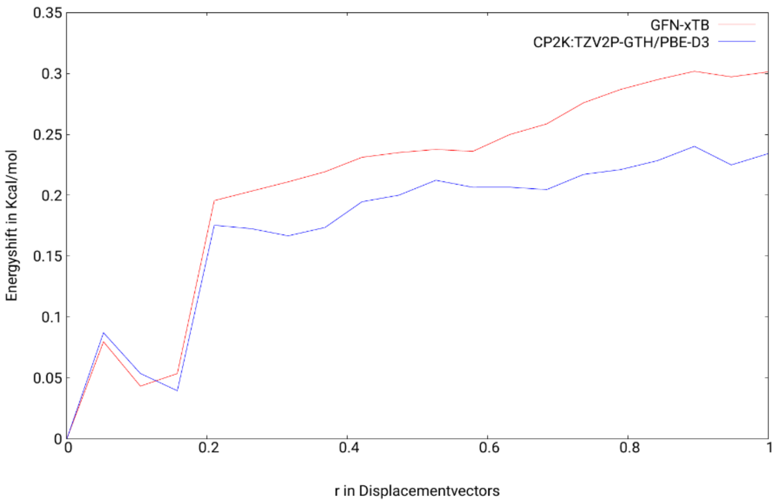

Figure S52. Reference pathway from A$\bar{A}$ → A$\bar{B}$ for comparing (GPW-DFT) and (GFN-xTB).

Calculation of cell-parameters for the full COF Systems (GFN-xTB)

Cell parameters for different components arrangements determined by cell optimization with simultaneous geometry optimization using GFN-xTB as implemented in CP2K are shown in Table. S12. The optimizations were performed using the conjugated gradients (CG) method and conducted until the total forces acting on the molecules were below $4.5 \cdot 10^{-4}$ Hartree/Bohr with the relative change in geometry being below $3.0 \cdot 10^{-3}$ Bohr and a pressure tolerance of below 100 bar with respect to the environment was reached. All optimizations were performed with fixed hexagonal-symmetry.

Table S13. Results cell parameter calculations determined by cell optimization with simultaneous geometry optimization using GFN-xTB for **PP-TAB** and **dPP-TAB**.

**COF arrangement**	***a* = *b* / Å**	***c* / Å**
PP-TAB-para-s10	58.524	7.26
PP-TAB-para-s11	58.577	7.31
PP-TAB-alt-s10	58.233	7.13
PP-TAB-alt-s11	58.145	7.21
dPP-TAB-alt-s10	58.198	7.25
dPP-TAB-alt-s11	58.242	7.32
dPP-TAB-para-s10	58.779	7.47
dPP-TAB-para-s11	58.608	7.37

Calculation of stacking types for the phenylphenanthridine-COFs and investigation of the role of methoxy Groups for total arrangements using various DFT-functionals

The influence of ordered and disordered methoxy-groups was investigated along with cell parameters. Relative energy differences between the structures were compared for the PBE and BP86[6,7] DFT functionals respectively, using Grimmes

DFT-D2 and DFT-D3 dispersion corrections. Further we compared to the semi empirical tight binding method GFN-xTB as implemented in CP2K. The energies were obtained after performing a cell optimization with simultaneous geometry optimization utilizing the method of conjugated gradients (CG) with constrained hexagonal symmetry.

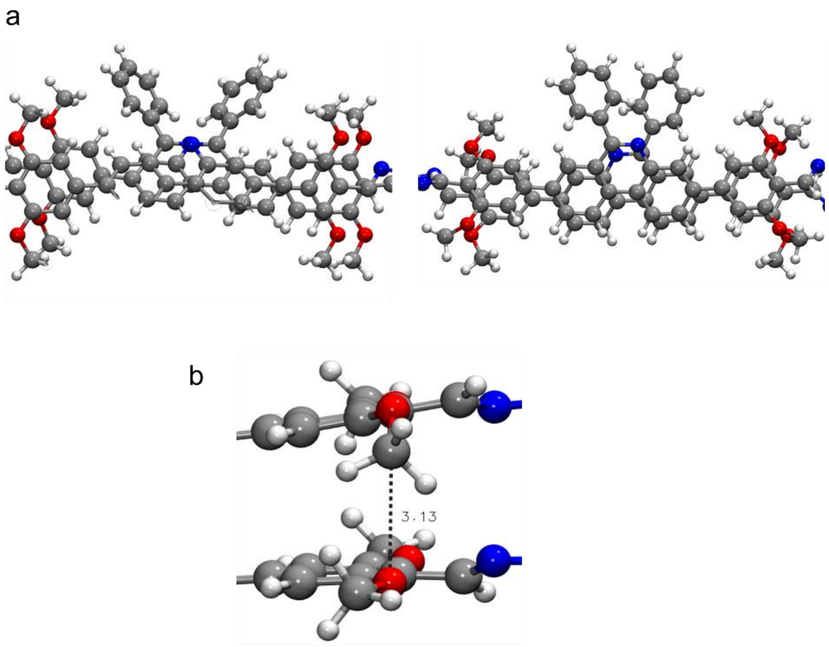

Figure S53. Influence of ordered and disordered methoxy-groups. (**a**) Excerpts of ordered (left) and disordered methoxy groups (right). (**b**) Approach of CH_3 group towards methoxy group of next layer observed after structure optimization.

We calculated the relative energy difference between unordered E_u and ordered E_o structures as:

$$\Delta E = E_o - E_u$$

The results are shown in Table S13. Cell parameters for ordered initial structures are shown in Table S13.
Cell parameters for disordered initial structures are shown in Table S14.

Table S14. Relative difference between the obtained energies of the optimized structures for the different methods.

**Utilized level of theory**	**Energy difference ΔE (Kcal/mol)**
TZV2P-GTH/PBE-D2	28.24
TZV2P-GTH/PBE-D3	31.38
TZV2P-GTH/BP-D2	11.90
TZV2P-GTH/GFN-xTB	3.14

Table S15. Cell parameters for ordered initial structures obtained after optimizing structures for the different methods.

**Utilized level of theory**	***a* = *b* / Å**	***c* / Å**
TZV2P-GTH/PBE-D2	58.125	7.720
TZV2P-GTH/PBE-D3	58.211	8.002

TZV2P-GTH/BP-D2	57.974	7.446
TZV2P-GTH/GFN-xTB	57.817	7.343

Table S16. Cell parameters for disordered initial structures obtained after optimizing structures for the different methods.

**Utilized level of theory**	***a = b* / Å**	***c* / Å**
TZV2P-GTH/PBE-D2	58.370	7.795
TZV2P-GTH/PBE-D3	58.460	8.034
TZV2P-GTH/BP-D2	58.252	7.503
TZV2P-GTH/GFN-xTB	58.222	7.232

Calculation of displacement maps using a plane-wave expansion (GFN-xTB)

To gain insight in the overall stability properties of the investigated COFs, we displaced two layers against each other and performed geometry optimizations with simultaneous cell optimization at each point. For the energy calculations we used GFN-xTB as implemented in CP2K. The cell optimizations were performed under the constraint of fixed hexagonal symmetry. For the starting point for the displacement, the centers of mass of both layers were placed at the origin of the elementary unit cell. We then displaced the layers in multiples of the cell vectors with the data points chosen as can be seen in the corresponding tables. The choice of sample points was motivated by assuming the COF-structures to be of D_6-symmetry (full hexagonal) in two dimensions and sample the lower triangle of the unit cell, the asymmetric unit, as shown in Figure S49, using a total of up to 16 data points. We then expanded the obtained energy surface in a plane-wave basis set, optimizing the coefficients c_n in a least square manner utilizing the singular value decomposition.

$$E(x, y) = \sum_n c_n \phi_n(x, y)$$

With plane wave basis functions corresponding to the assumed hexagonal symmetry as shown previously, where n is the index of the basis function and x, y are the spatial positions. As we used a polar representation, **r** is the two-dimensional position vector and φ is the angle between the components of the wave vector $\mathbf{k}_n = n_1 \mathbf{b}_1 + n_2 \mathbf{b}_2$ with $n_1 + n_2 < n$.

$$\phi_n(\phi) = \sum_i^6 \cos\left(\boldsymbol{k}_{n,i} \boldsymbol{r}\right)$$

The wave vectors $k_{n,i}$ build the parts for the PW-basis function *n* with hexagonal symmetry, and are given in terms of the wave vectors absolute value $k_n = |\mathbf{k}_n|$ as:

$$\boldsymbol{k}_{n,i} = k_n \cdot \begin{pmatrix} \cos(\alpha \cdot (\phi + \frac{\pi}{3}(i-1))) \\ \sin(\alpha \cdot (\phi + \frac{\pi}{3}(i-1))) \end{pmatrix}$$

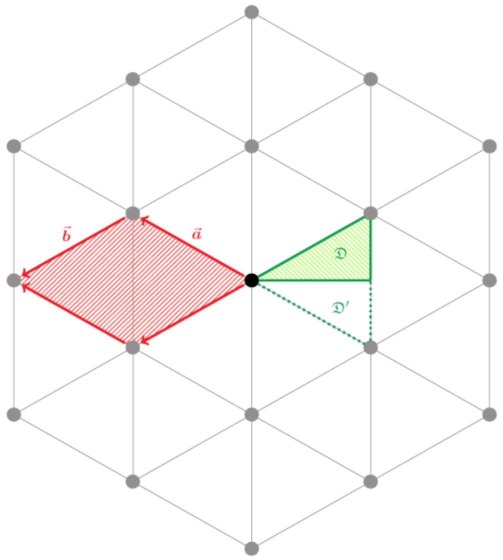

Figure S54. Schematic representation of the utilized symmetry assumptions entering in the plane wave fit for the potential energy surface. The treatment of the hexagonal unit cell shown in red with cell vectors a and b was further simplified by using the properties of the D6 symmetry, which leaves only the green triangle which spans a quarter of the original area of the unit cell as sampling area. The remaining part of the unit cell that is constructed by mirroring the interpolated triangle D is indicated by D' which then spans the half of the utilized unit cell indicated in red, and thus leads to sufficient information to construct the full unit cell.

In the following the grid points used for the interpolations are listed with their respective in plane positions given in multiples of the cell vectors **a** and **b** as well as the calculated total electronic energy in kcal/mol.

Table S17. Grid points for the basis set expansion (**PP-TAB**).

a	***b***	**Total Energy (kcal/mol)**
0	0	-779.247
0.1	0.1	-779.109
0.1	0	-779.226
0.2	0.2	-779.031
0.2	0	-779.194
0.277	0.111	-779.164
0.3	0.1	-779.186
0.3	0.2	-779.285
0.3	0	-779.267
0.4	0.1	-779.094
0.4	0.2	-779.209
0.4	0	-779.093
0.5	0	-779.076
0.3	0.3	-779.597
0.333	0.333	-779.375

Table S18. Grid points for the basis set expansion (**mPP-TAB**).

a	***b***	**Total Energy (kcal/mol)**
0	0	-874.051
0.1	0.1	-873.948

0.1	0	-874.040
0.2	0.1	-873.893
0.2	0.2	-873.962
0.2	0	-873.928
0.277	0.111	-873.907
0.3	0.1	-874.379
0.3	0.2	-873.997
0.3	0.3	-874.618
0.3	0	-874.040
0.333	0.333	-874.683
0.4	0.1	-873.911
0.4	0.2	-874.204
0.4	0	-873.896
0.5	0	-873.896

Table S19. Grid points for the basis set expansion (**dPP-TAB**).

a	***b***	**Total Energy (kcal/mol)**
0	0	-968.840
0.1	0.1	-968.818
0.1	0	-968.837
0.2	0.1	-968.681
0.2	0.2	-968.667
0.2	0	-968.707
0.277	0.111	-968.678
0.3	0.1	-968.781
0.3	0.2	-968.689
0.3	0.3	-968.534
0.3	0	-968.705
0.333	0.333	-968.507
0.4	0.1	-968.647
0.4	0.2	-968.674
0.4	0	-968.693
0.5	0	-968.735

Investigations of arrangements for the full unit cells (Layer Curving)

For COF arrangements with layers shifted from the initial A$\bar{A}$ stacking, we could observe an increase of layer curving depending on the distance from the eclipsed mode. The curving is visible in the cell parameters a, b by a shortening of their respective length after optimization. An exemplary structure with the upper layer shifted by 0.2*a and 0.1*b for the Methoxy-1-COF is shown in Figure S55, where the curving leads to an in-plane cell vector length of 56.709 Angstrom compared to 58.872 Angstrom in the eclipsed case, which is observed to be almost planar.

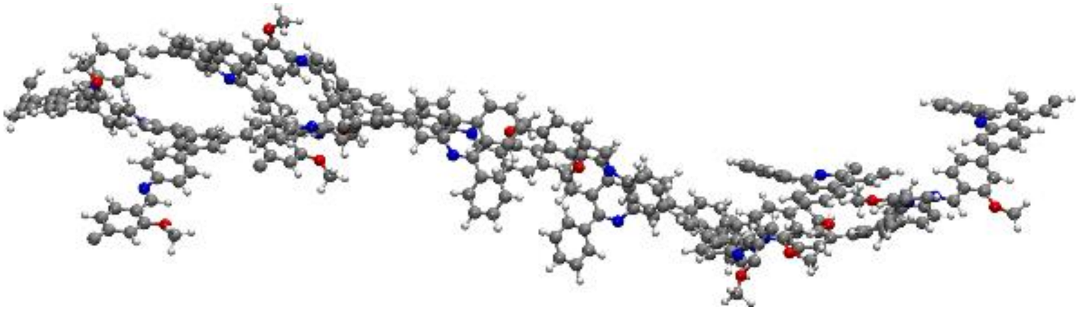

Figure S55. Curving in the arrangement for an optimized structure with initial upper layer displacement of (0.2,0.1).

S11 SEM and TEM

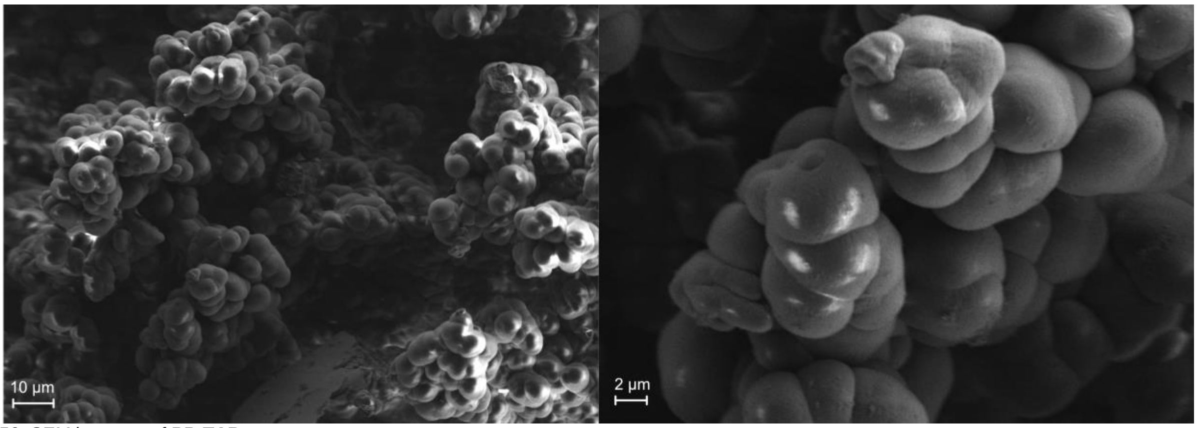

Figure S56. SEM imagess of **PP-TAB**.

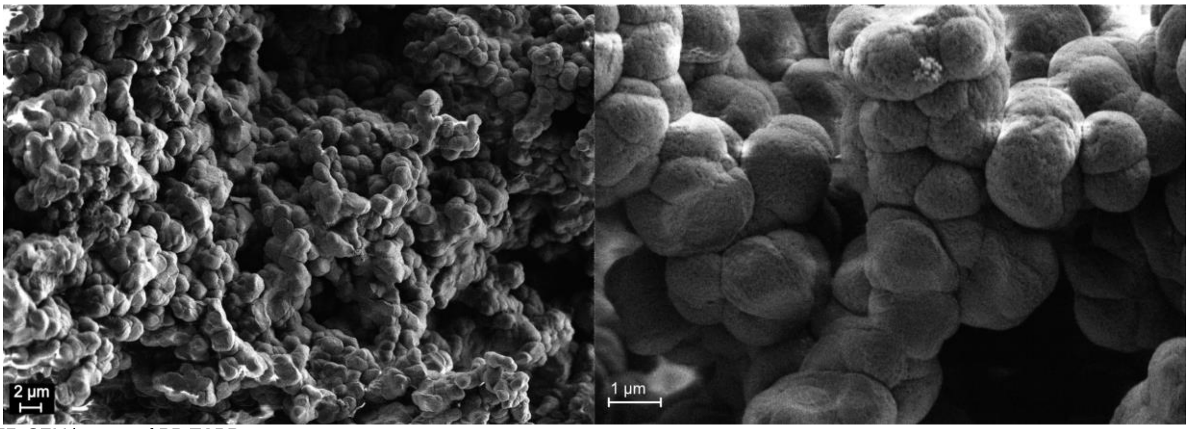

Figure S57. SEM images of **PP-TAPB**.

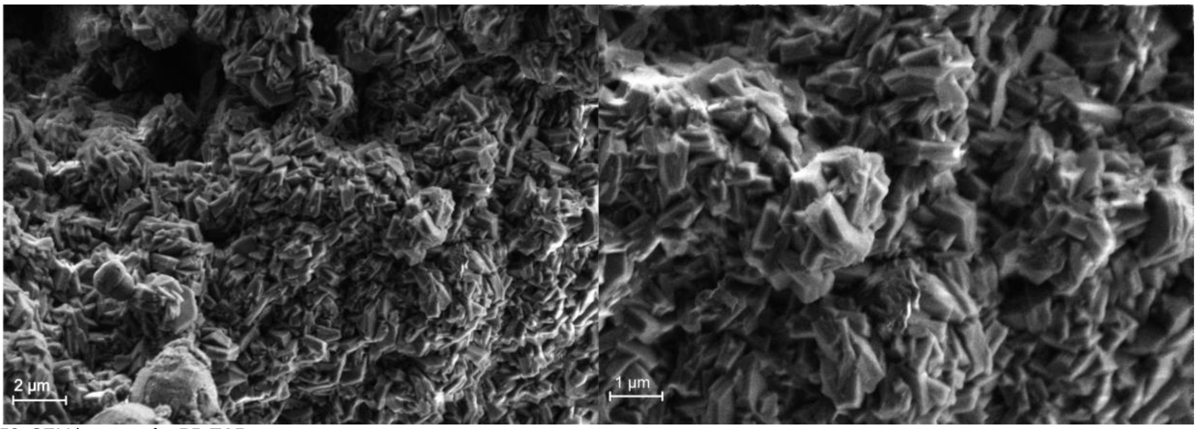

Figure S58. SEM images of **mPP-TAB**.

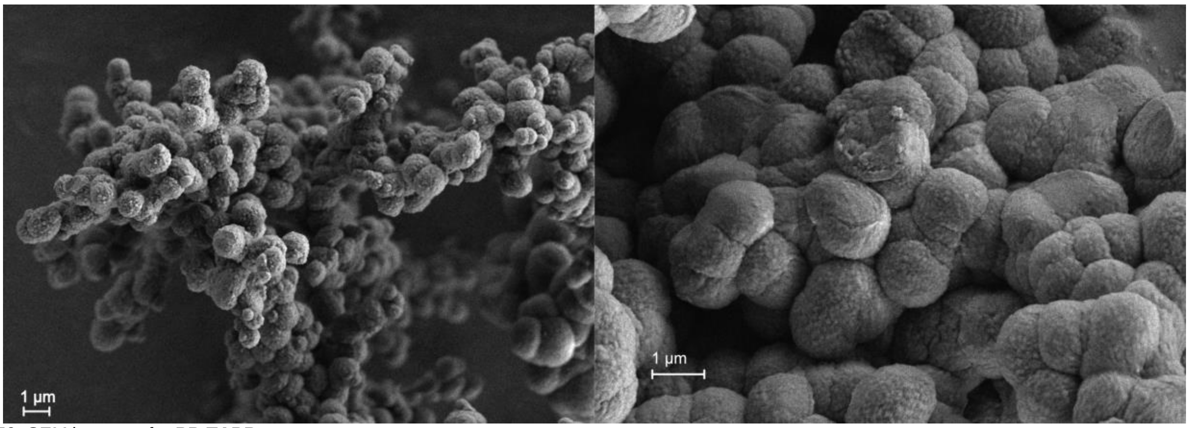

Figure S59. SEM images of **mPP-TAPB**.

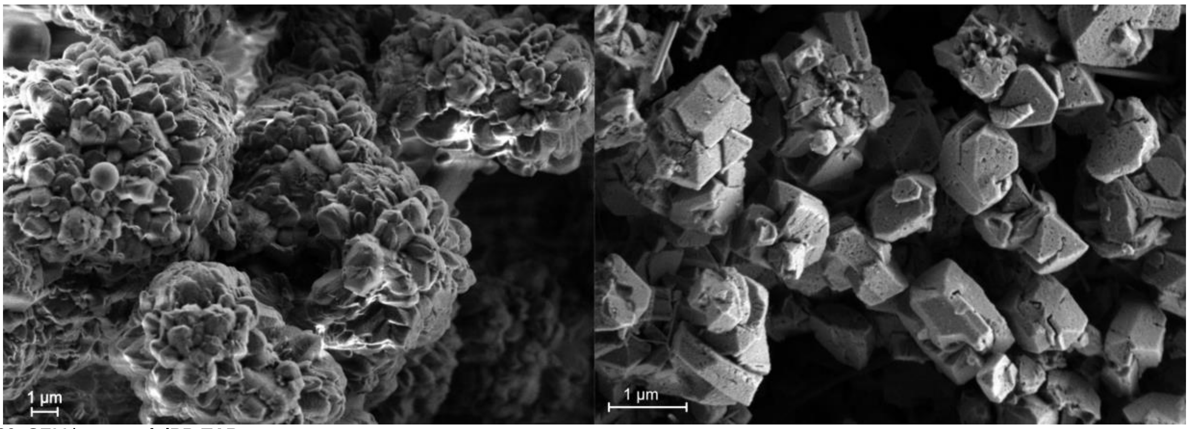

Figure S60. SEM images of **dPP-TAB**.

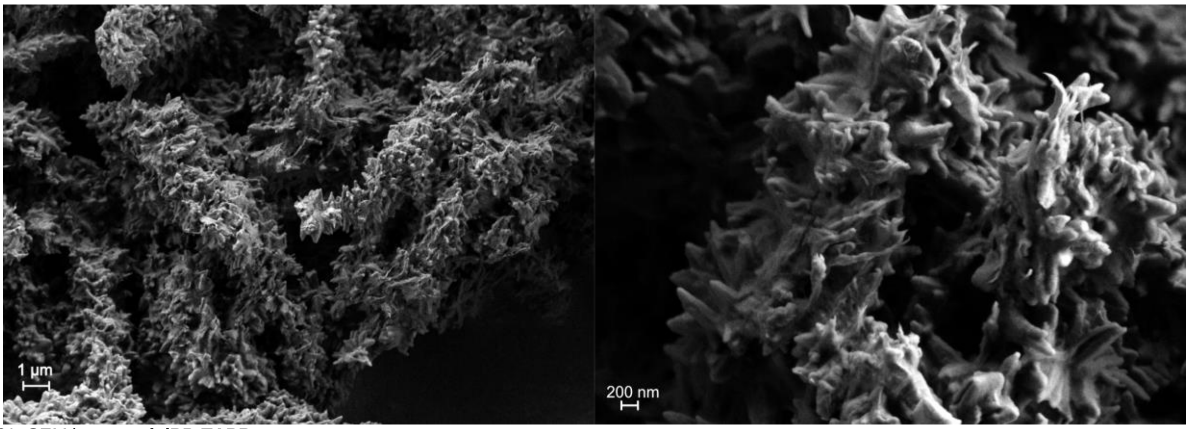

Figure S61. SEM images of **dPP-TAPB**.

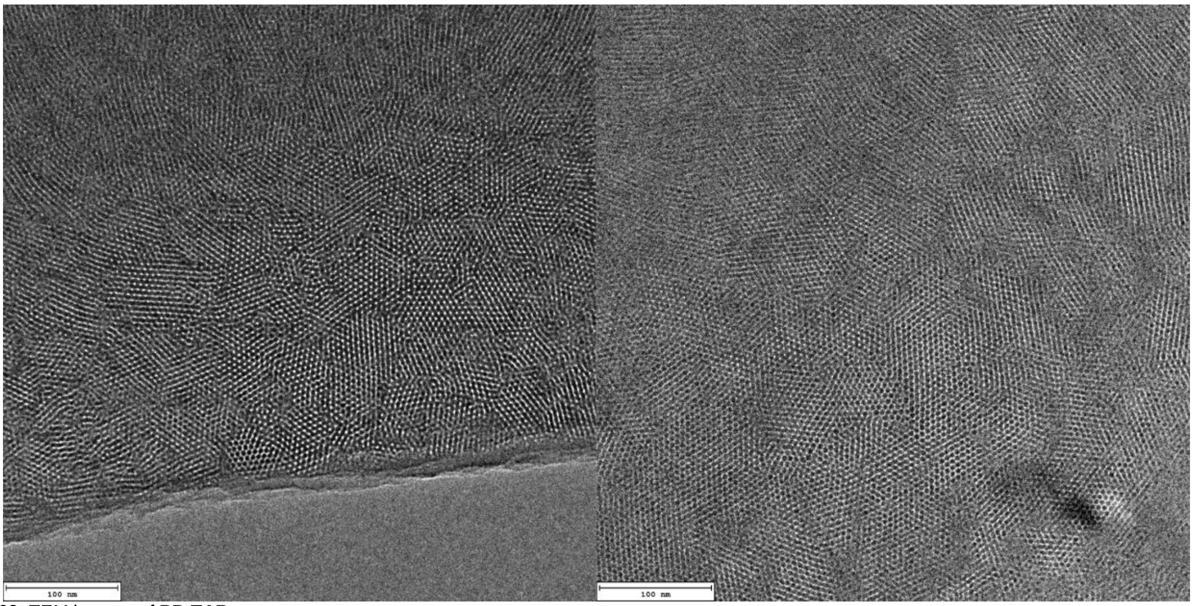

Figure S62. TEM images of **PP-TAB**.

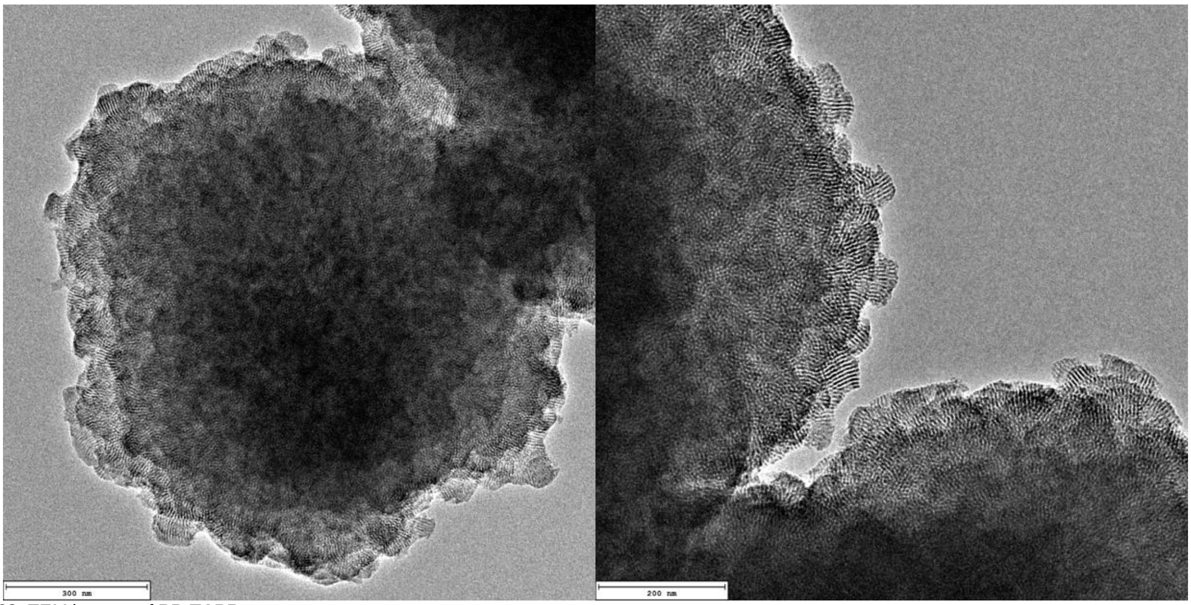

Figure S63. TEM images of **PP-TAPB**.

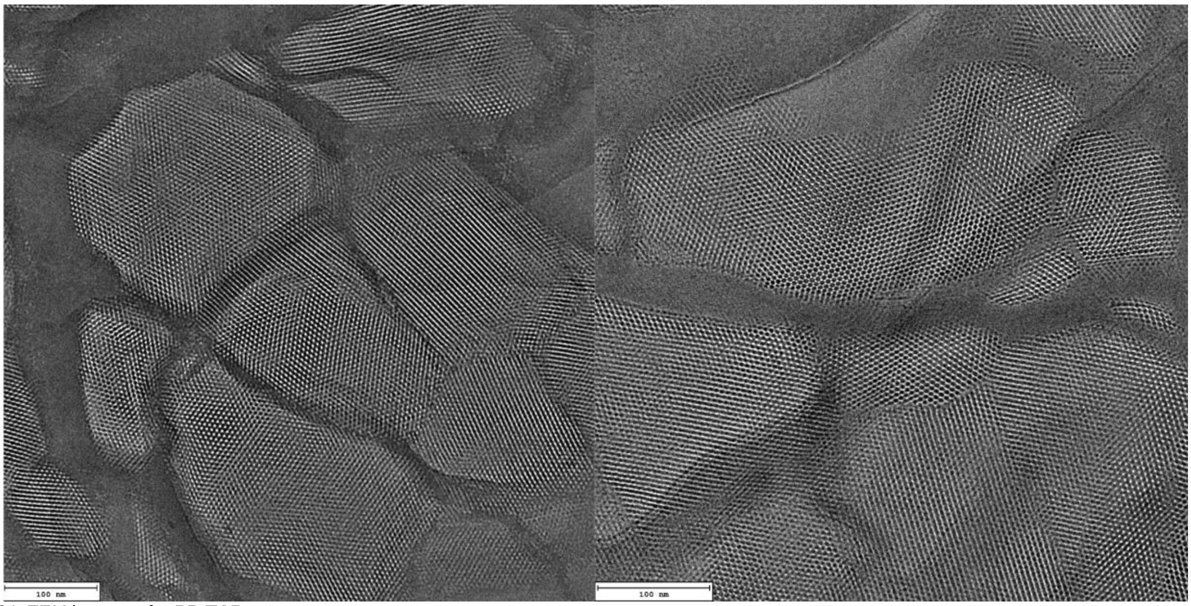

Figure S64. TEM images of **mPP-TAB**.

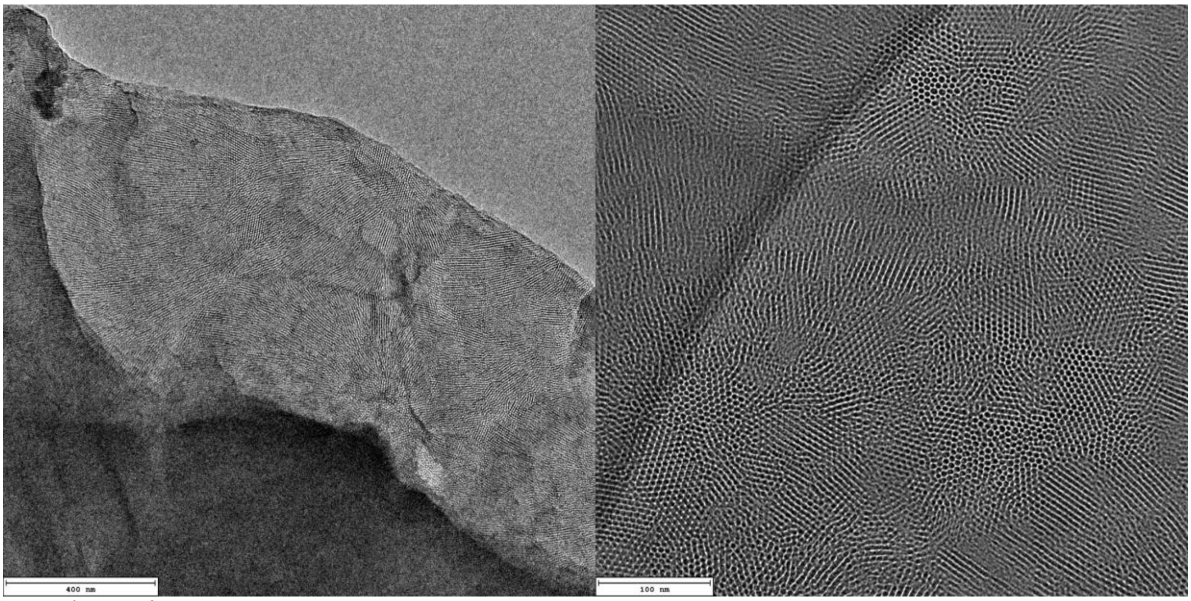

Figure S65. TEM images of **mPP-TAPB**.

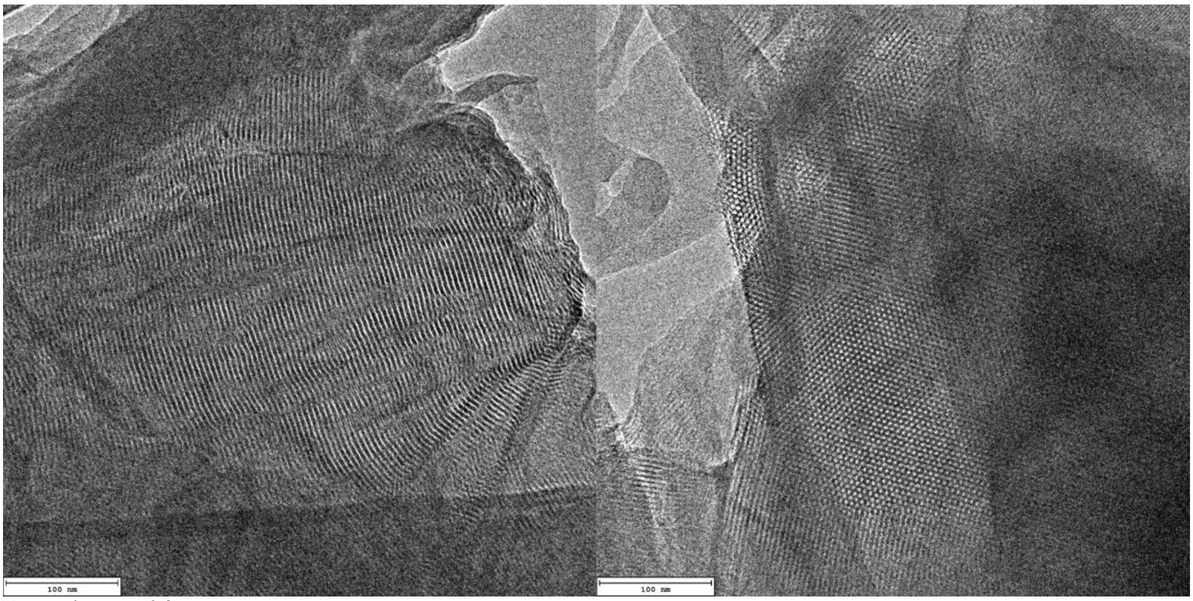

Figure S66. TEM images of **dPP-TAB**.

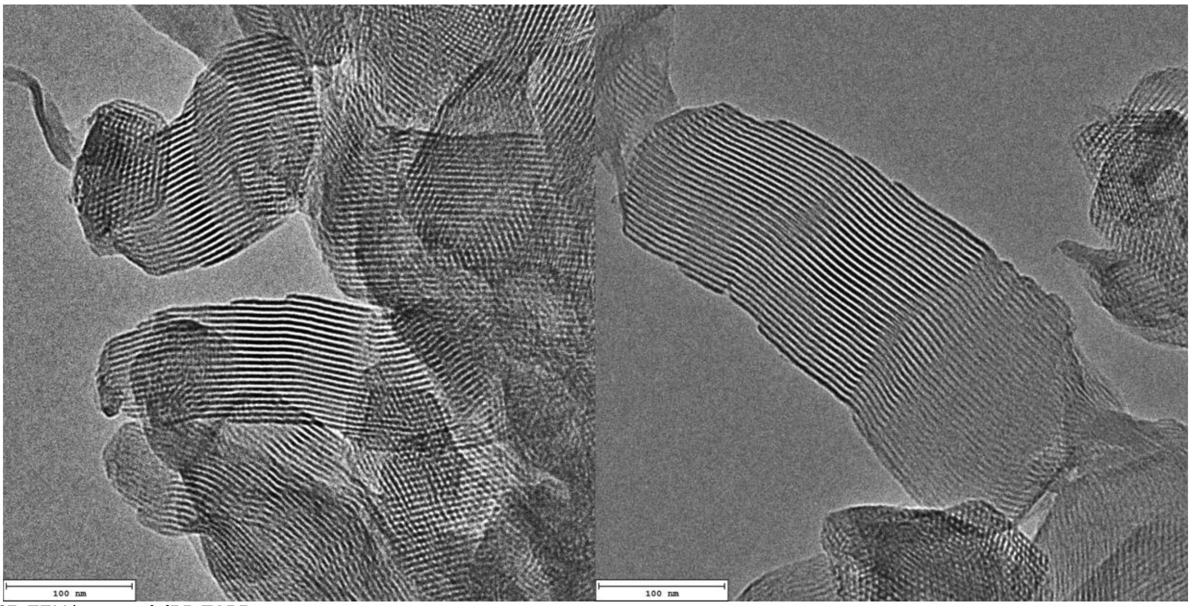

Figure S67. TEM images of **dPP-TAPB**.

References

[1] Q. Fang, Z. Zhuang, S. Gu, R. B. Kaspar, J. Zheng, J. Wang, S. Qiu, Y. Yan, *Nat. Commun.* **2014**, *5*, 4503.

[2] G. R. Fulmer, A. J. M. Miller, N. H. Sherden, H. E. Gottlieb, A. Nudelman, B. M. Stoltz, J. E. Bercaw, K. I. Goldberg, *Organometallics* **2010**, *29*, 2176–2179.

[3] Y. Chen, F. Li, Z. Bo, *Macromolecules* **2010**, *43*, 1349–1355.

[4] M. Movassaghi, M. D. Hill, *Org. Lett.* **2008**, *10*, 3485–3488.

[5] D. J. F. M. J. Frisch, G. W. Trucks, H. B. Schlegel, G. E. Scuseria, M. A. Robb, J. R. Cheeseman, G. Scalmani, V. Barone, G. A. Petersson, H. Nakatsuji, X. Li, M. Caricato, A. V. Marenich, J. Bloino, B. G. Janesko, R. Gomperts, B. Mennucci, H. P. Hratchian, J. V., **2016**, Gaussian 16, Revision C.01.

[6] A. D. Becke, *Phys. Rev. A* **1988**, *38*, 3098–3100.

[7] J. P. Perdew, A. Zunger, *Phys. Rev. B* **1981**, *23*, 5048–5079.
